# Cultural adaptation of internet- and mobile-based interventions for mental disorders: a systematic review

**DOI:** 10.1038/s41746-021-00498-1

**Published:** 2021-08-25

**Authors:** Kerstin Spanhel, Sumeyye Balci, Felicitas Feldhahn, Juergen Bengel, Harald Baumeister, Lasse B. Sander

**Affiliations:** 1 Department of Rehabilitation Psychology and Psychotherapy, Institute of Psychology, University of Freiburg, Freiburg, Germany; 2 Department of Clinical Psychology and Psychotherapy, Institute of Psychology and Education, Ulm University, Ulm, Germany

**Keywords:** Psychiatric disorders, Therapeutics, Developing world, Culture, Psychology

## Abstract

Providing accessible and effective healthcare solutions for people living in low- and middle-income countries, migrants, and indigenous people is central to reduce the global mental health treatment gap. Internet- and mobile-based interventions (IMI) are considered scalable psychological interventions to reduce the burden of mental disorders and are culturally adapted for implementation in these target groups. In October 2020, the databases PsycInfo, MEDLINE, Embase, Cochrane Central Register of Controlled Trials, and Web of Science were systematically searched for studies that culturally adapted IMI for mental disorders. Among 9438 screened records, we identified 55 eligible articles. We extracted 17 content, methodological, and procedural components of culturally adapting IMI, aiming to consider specific situations and perspectives of the target populations. Adherence and effectiveness of the adapted IMI seemed similar to the original IMI; yet, no included study conducted a direct comparison. The presented taxonomy of cultural adaptation of IMI for mental disorders provides a basis for future studies investigating the relevance and necessity of their cultural adaptation.

PROSPERO registration number: CRD42019142320.

## Introduction

The World Health Organisation has called to take action in order to address global health inequalities^1^ as one of the major global health challenges^2–5^. Particularly when it comes to mental health, the treatment gap in low- and middle-income countries (LMIC) is estimated to be far higher than that in high-income countries: Whereas ~76–85% of people with a serious mental disorder living in LMIC do not get treated, ~35–50% of people living in high-income countries do not get treated^1,6,7^. Similarly, migrants and refugees make less use of mental health services^8–10^, although they face many stressors before, during, and after migration^11^ and show an increased prevalence of mental disorders^12–14^. Such a mental health treatment gap can also be observed in indigenous people, caused by lower mental health^15,16^ and lower use of health services^17^. In order to improve global health equality, existing barriers of these populations need to be addressed on a structural and individual level.

Existing structural barriers, such as a lack of mental health services and difficulties in accessing services^18–21^, could be reduced by low intensity interventions^22–25^. Low intensity interventions are interventions aiming to be easy to access by a high number of people, due to a resource-saving and flexible delivery (e.g., self-help interventions, interventions delivered by non-professionals)^26^. Internet- and mobile-based interventions (IMI) as one kind of low intensity intervention offer anonymity, temporal and local independency, easy accessibility, and scalability^27–30^ and are thus suggested to hold potential in overcoming structural healthcare barriers^31,32^. Despite reports of lower treatment completion rates^33^, IMI have been proven effective in preventing and treating mental disorders^34–38^. However, they were mostly developed for and evaluated in majority populations from high-income countries, and were found to be less effective for people with a differing cultural or ethnic background^39^. When it comes to the use of healthcare, populations living in LMIC, as well as migrants, refugees, or indigenous people face a range of individual barriers, such as language and cultural barriers (e.g., understanding of disease and treatment processes) or poor knowledge on the healthcare system^17–21,40^. These individual barriers may be reduced by considering cultural aspects in the intervention development, i.e., including knowledge on potential cultural differences^41^. However, newly developing interventions for each population requires significant effort. A more resource-saving possibility could be to use the high number of already existing and evaluated psychological treatments and to culturally adapt them for the new target groups^42,43^.

A cultural adaptation should be conducted in a structured, systematic way by following adaptation guidelines^44,45^. Various research groups established such guidelines, focussing (a) on specific treatment components that should be culturally adapted^46–51^, or (b) on the procedure that should be followed to gradually adapt the treatment^45,52–54^. The Ecological Validity Framework by Bernal et al.^47^ was the first model describing in detail what to culturally adapt. The authors recommended the consideration of eight intervention components, including language (translation, differences in regional or subcultural groups), persons (patient–therapist relationship, roles), metaphors (symbols, sayings), content (values, customs, traditions), concepts (theoretical model of the treatment), goals (agreement of therapist and patient), methods (procedures for achieving the treatment goals), and context (broader social, economic, and political contexts). This model was widely reused and can be seen as a base for later frameworks. For example, Resnicow et al.^50^ differentiated the components to adapt into surface characteristics, such as language, locations, and persons, and deep structure characteristics of treatment, which comprise the inclusion of cultural, social, environmental, and historical factors to consider the understanding of disease and its treatment of the respective clients, such as cultural values (e.g., family) and specific stressors (e.g., racism). Regarding frameworks on the procedure of culturally adapting treatments, Barrera and Castro summarised various suggestions of other frameworks in their Heuristic Framework for the Cultural Adaptations of Interventions^54^. They describe the procedural components of how to culturally adapt by (1) gathering information by literature searches or qualitative research, (2) developing a preliminary adaptation based on this information, (3) testing the preliminary adaptation in case or pilot studies, and (4) refining the adaptation based on the findings of case or pilot studies.

Considering both the suggested content and procedural components of cultural adaptation intends to facilitate access to psychological treatments for people with a cultural background differing from that of the original target group^44^. Correspondingly, findings of meta-analyses aiming to investigate the relevance and necessity of cultural adaptation suggest that, in populations that the intervention was originally not developed for, culturally adapted treatments are more effective as compared to non-adapted versions of the treatment^55,56^. The higher the extent of cultural adaptation, the higher seems their effectiveness^57,58^. Based on this revealed relevance of culturally adapting face-to-face treatments, it may also be of relevance to culturally adapt IMI to reach out to people with a cultural background differing from the original target group^25,59,60^. Various research groups culturally adapted IMI to fit new target groups and showed their effectiveness^61–63^. Furthermore, a meta-analysis indicated that an enhanced cultural adaptation of an IMI was associated with its increased effectiveness in the respective target group^59^. However, studies directly comparing the effectiveness or acceptance of culturally adapted versus non-/less-adapted IMI are lacking^59,64–66^, which would allow for drawing conclusions on the added value of cultural adaptation. A precondition for such comparison trials and for examining the relevance of adapting specific components following a specific procedure would be a systematic and well-documented adaptation^67,68^. Yet, components and procedures considered in the cultural adaptation of IMI are often only poorly reported^69–73^. Thus, research on the cultural adaptation of IMI is still in its early stages^66^, and there is a particular lack of knowledge on specific aspects of IMI that might need cultural adaptation in addition to the components adapted in face-to-face treatments. In this context, following aspects of evaluation frameworks of IMI could provide an orientation, such as methodological components to enhance the user engagement with the IMI, its ease of use, or design and aesthetics^74–76^.

Thus, this review aims to

1. systematically identify and summarise content, methodological, and procedural components considered in the previous cultural adaptations of IMI for mental disorders;
2. summarise results on the adherence and effectiveness of culturally adapted IMI.

By providing a taxonomy on the cultural adaptation of IMI, this review may inform researchers on systematically developing scalable interventions for underserved people.

## Results

### Study selection

**Fig. 1:**
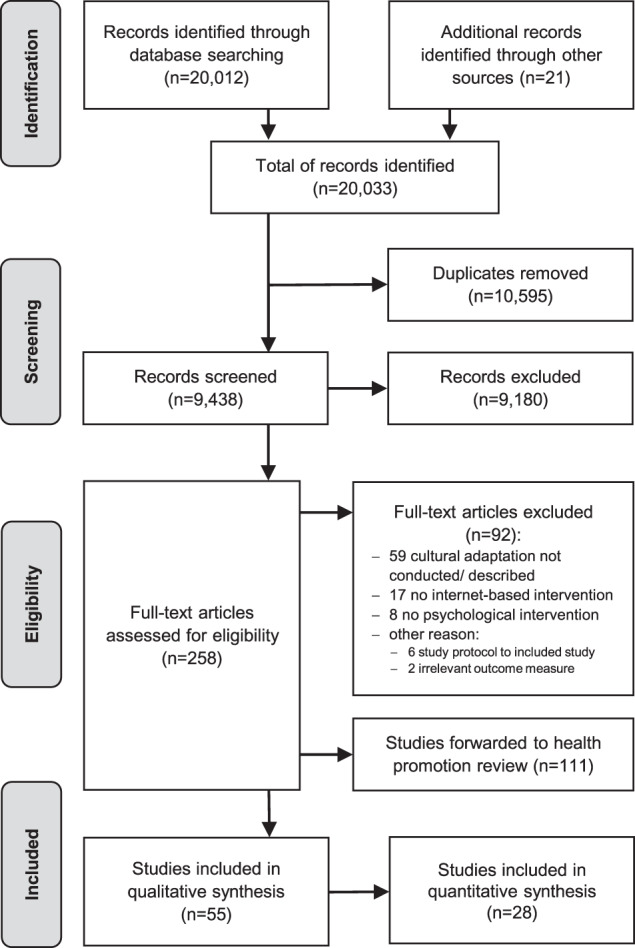
PRISMA study flow chart. Process of study identification, screening, eligibility verification, and inclusion decision (adapted from Liberati et al.^115^).

A total of 20,012 articles were retrieved from the electronic databases; 21 articles were added by hand searching relevant articles. After removing duplicates, 9438 titles and abstracts were screened, leading to 258 records that were included in the full-text screening. In 24% of the decisions, the inclusion of a study was discussed between the reviewers. Finally, 55 studies were found eligible for the present qualitative synthesis, of which 28 provided quantitative data (see Fig. 1).

### Study characteristics

All 55 included articles were published between 2012 and 2020, with 38 (69.1%) published in 2018–2020. Eight of them were study protocols, three of which additionally described conducted qualitative studies. Across the studies conducted in the included articles, a total of 3789 participants took part, plus a sample of 23,235 participants of a study analysing existing data of a patients cohort^77^. The studies were conducted or planned to be conducted in various countries: 26 in North America and Europe, 16 in Asia, 5 in Australia, 4 in the Middle East, and 2 in South or Central America. Similarly, the ethnicities of the participants were diverse across the studies: 34.6% of the studies included Asian participants, 26.9% Arabic, 15.4% European, 11.5% Northern American (including 7.7% American Indian/Alaskan Native), and 11.5% participants with other ethnic backgrounds. The included articles reported on the cultural adaptation of 42 different interventions. For 23 interventions, detailed information on their cultural adaptation had been published; for 19 interventions, less detailed information was available in published articles. Partly, more details were provided by the authors upon request (*n* = 9)^77–85^. Sixteen interventions described in the articles addressed depression, also in combination with anxiety, ten addressed stress or traumatisation, four substance abuse disorders, and the remaining interventions addressed other mental disorders or a combination of the named ones.

An overview of the study characteristics can be found in Table 1. Details on the interventions used in the studies are illustrated in Supplementary Table 3.

**Table 1:** Characteristics of the included articles.

First author (year)	Data assessment	Step of cultural adaptation (CA)^a^	Type of assessments	Type of sample	Sample size	Age *M*(SD)	% female	Level of education	Study conduction	% quality score^b^
Abi Ramia (2018)^69^	Qualitative	Inform CA	Interviews, focus groups	Health professionals, target group (community members, immigrants)	84	–	59.4	–	Lebanon	83.3
Abuwalla (2017)^123^	Qualitative	Inform CA	Questionnaires, narrative review	Health professionals	4	–	50.0	University	USA	50.0
Arjadi (2018)^70^	Qualitative	Inform CA	Interviews, questionnaires	Health professionals, lay counsellors, community members, patients	54	–	–	Secondary, university	Indonesia	73.8
Arjadi (2018)^124^	Quantitative	Evaluate CA (RCT with 2, 4, 6, 8, 10, 12, 24 w)	Questionnaires	Target group (community members)	313	24.5 (5.1)	80.8	All levels	Indonesia	78.6
Bolinski (2018)^78^	Planned: quantitative	Planned: evaluate CA (RCT with FU: 1, 2, 6, 12 m)	Planned: Interviews, questionnaires	Target group (students)	–	–	–	Secondary, university	The Netherlands	87.9
Brooks (2013)^87^	Qualitative	Inform CA; evaluate CA (pilot study with FU: 4, 6, 9, 12 m)	Interviews, discussions, focus groups, website analytics, questionnaires	Health professionals, community members, target group (indigenous patients)	–	–	–	–	USA	35.7
Burchert (2018)^111^	Qualitative	Inform CA	Interviews, focus groups	Target group (refugees)	128	33.3 (11.0)	50.0	All levels	Germany, Sweden, Egypt	85.7
Campbell (2015)^92^	Qualitative, quantitative	Evaluate CA (pilot study with FU: 9 w)	Interviews, questionnaires	Target group (indigenous people)	40	37.5 (10.9)	47.5	All levels	USA	77.1
Chen (2020)^81^	Quantitative	Evaluate CA (controlled trial with FU: 8 w)	Questionnaires	Target group (community members)	255	25.6 (5.6)	75.7	All levels	China	66.7
Choi (2012)^125^	Quantitative	Evaluate CA (RCT with FU: 3 m)	Interviews, questionnaires	Target group (immigrants)	55	39.0 (11.7)	80.0	Secondary, university	Australia	88.1
DaPonte (2018)^126^	Quantitative	Evaluate CA (pilot study with FU: 3 m)	Interviews, questionnaires	Target group (community members)	31	42.6 (–)	77.4	Secondary, university	Canada	69.1
Eylem (2021)^127^	Quantitative	Evaluate CA (pilot RCT with FU: 2, 4, 6, 12 w)	Interviews, questionnaires	Target group (immigrants)	18	33.5 (8.4)	72.2	All levels	The Netherlands	83.3
Garabiles (2019)^128^	Qualitative	Inform CA	Interviews, discussions, focus groups, think aloud	Target group (labour migrants)	31	33.9 (8.7)	57.1	All levels	China	78.6
Gorman (2013)^129^	Qualitative	Inform CA	Interviews, focus groups	Health professionals, target group (indigenous people)	21	–	100.0	–	USA	81.0
Harper Shehadeh (2020)^89^	Quantitative	Evaluate CA (pilot study with FU: 8 w)	Interviews, questionnaires, website analytics	Target group (community members, immigrants)	129	27.7 (–)	77.5	All levels	Lebanon	79.2
Hiratsuka (2019)^130^	Quantitative	Evaluate CA (pilot study with FU: 6,12 w)	Questionnaires, website analytics	Target group (indigenous people)	24	49 (14)	71	All levels	USA	71.4
Imamura (2019)^80^	Planned: quantitative	Planned: Evaluate CA (RCT with FU: 3, 7 m)	Questionnaires	Target group (workers)	–	–	–	–	Vietnam	87.9
Ip (2016)^93^	Quantitative	Evaluate CA (RCT with FU: 4, 8, 12 m)	Questionnaires	Target group (adolescents)	257	14.6 (0.8)	68.1	Secondary	Hong Kong, China	76.2
Juniar (2019)^71^	Qualitative, planned: quantitative	Inform CA; planned: evaluate CA (pilot study with FU: 10 w)	Interviews, focus groups; planned: questionnaires, website analytics	Focus group: target group (students), interviews: health professionals	29 (focus groups: 25)	26.7 (3.2)	48.0	University	The Netherlands	85.7
Kaal (2020)^82^	Planned: quantitative	Planned: Evaluate CA (RCT with FU: 2, 6 m)	Questionnaires	Target group (community members)	–	–	–	–	Estonia	75.8
Kanuri (2020)^96^	Qualitative, quantitative	Inform CA; evaluate CA (pilot study with FU: 4 w)	Questionnaires, focus groups	Target group (students)	15	19.1 (1.3)	13.3	Secondary	India	75.0
Kayrouz (2015)^97^	Quantitative	Evaluate CA (pilot study with FU: 2, 3 m)	Questionnaires	Target group (immigrants)	11	33.6 (9.0)	72.7	Secondary, university	Australia	88.1
Kayrouz (2016)^98^	Quantitative	Evaluate CA (pilot study with FU: 2, 3 m)	Questionnaires	Target group (immigrants)	13	37.1 (12.5)	38.5	Secondary, university	Australia	83.3
Kayrouz (2016)^99^	Quantitative	Evaluate CA (pilot study with FU: 2, 3 m)	Questionnaires	Target group (immigrants)	36	36.2 (12.1)	58.3	Secondary, university	Australia	85.7
Knaevelsrud (2015)^61^	Quantitative	Evaluate CA (RCT with FU: 1, 3 m)	Questionnaires	Target group (community members)	159	28.1 (7.4)	71.7	Secondary, university	Germany	76.2
Lal (2020)^131^	Qualitative	Inform CA	Focus groups, questionnaires	Health professionals, target group (patients)	26	Range: 19–56	65.4	All levels	Canada	66.7
Lin (2020)^106^	Quantitative	Evaluate CA (RCT with FU: 8 w)	Questionnaires	Target group (community members)	80	23.6 (3.5)	66.3	–	China	81.0
Lindegaard (2020)^63^	Quantitative	Evaluate CA (pilot RCT with FU: 3, 8 w)	Interviews, questionnaires	Target group (immigrants, refugees)	59	37.5 (11.4)	42.4	All levels	Sweden	95.2
Luo (2021)^132^	Quantitative	Evaluate CA (RCT with FU: 6 w, 6 m)	Questionnaires	Target group (community members)	372	17.4 (1.4)	100.0	–	China	83.3
Muroff (2017)^133^	Quantitative	Inform CA; evaluate CA (study with FU: 6 m)	Usage data	Target group (immigrated patients)	79	41.1 (8.8)	11.4	All levels	USA	50.0
Muroff (2019)^105^	Quantitative	Evaluate CA (study with FU: 6 m)	Interviews, questionnaires	Target group (immigrated patients)	79	41.1 (8.8)	11.4	All levels	USA	76.2
Nygren (2018)^134^	Qualitative, quantitative	Inform CA; evaluate CA (pilot study)	Focus groups, questionnaires	Pilot study: target group (immigrants/refugees), focus groups: health professionals, target group	114 (pilot study: 105)	Range: 20–60	–	Secondary, university	Sweden	70.8
Nygren (2019)^135^	Quantitative	Evaluate CA (RCT with FU: 2, 11 m)	Questionnaires	Target group (immigrants)	50	33.9 (8.2)	46.0	All levels	Sweden	85.7
Okujava (2019)^84^	Quantitative	Evaluate CA (pilot study with FU: ~10 w)	Questionnaires	Target group (patients)	52	33.5 (–)	65.4	–	Georgia	42.8
Paris (2018)^100^	Quantitative	Evaluate CA (RCT with FU: 2, 3, 6, 12 m)	Questionnaires	Target group (immigrated patients)	92	42.9 (11.5)	32.6	All levels	USA	71.4
Patel (2017)^136^	Qualitative	Inform CA; evaluate CA (pilot with FU: 2 m)	Questionnaires	Panel: health professionals; pilot study: target group (students)	23 (pilot study: 20)	21 (–)	70.0	University	China	64.3
Pinto-Bruno (2019)^137^	Planned: quantitative	Planned: Evaluate CA (RCT with FU: 3, 6 m)	Questionnaires	Target group (unpaid care givers)	–	–	–	–	The Netherlands	81.8
Rahmadiana (2019)^79^	Qualitative, planned: quantitative	Inform CA; planned: evaluate CA (study with FU: 7 w)	Focus groups, questionnaires	Target group (students)	70	–	–	University	Indonesia	94.4
Salamanca-Sanabria (2019)^73^	Qualitative	Inform CA	Questionnaires	Health professionals, target group (students)	12	–	–	University	Colombia	75.0
Salamanca-Sanabria (2020)^138^	Quantitative	Evaluate CA (RCT with FU: 7 w, 3 m)	Questionnaires	Target group (students)	214	22.2 (4.7)	71.5	Secondary, university	Colombia	88.1
Saulsberry (2013)^139^	Qualitative	Inform CA	Interviews, focus groups, questionnaires	Focus groups: target group (immigrated adolescents), interviews: parents of target group	17	Range: 15–18	–	–	USA	73.8
Shala (2020)^90^	Qualitative	Inform CA	Interviews, focus groups	Health professionals, target group (immigrants)	42	35 (–)	66.7	All levels	Germany, Switzerland	78.6
Silva (in prep)^140^	Quantitative	Inform CA (RCT with FU: 2, 3, 6, 12 m)	Questionnaires	Target group (immigrated patients)	92	42.9 (11.5)	32.6	All levels	USA	74.4
Silva (2020)^101^	Quantitative	Evaluate CA (RCT with FU: 2, 6 m)	Questionnaires	Target group (immigrated patients)	83 (sub-sample)	43.0 (11.5)	33.7	All levels	USA	61.9
Sit (2020)^141^	Qualitative	Inform CA	Interviews, focus groups	Health professionals, target group (young adults)	41 plus 6 experts	19.6 (1.3)	58.5	Secondary	China	71.4
Sobowale (2013)^142^	Qualitative	Inform CA	Interviews, focus groups, questionnaires	Focus group: target group (students); interviews: health professionals, other experts; public health campaign: target group, associated people	16	Range: 18–21	–	Secondary, university	Hong Kong, China	81.0
Spanhel (2019)^91^	Qualitative	Inform CA	Interviews, think aloud	Health professionals, target group (refugees)	12	41.8 (13.2)	41.7	–	Germany	78.6
Teles (2020)^143^	Qualitative	Inform CA	Questionnaires	Health professionals	4	–	–	–	Portugal	64.3
Titov (2018)^77^	Quantitative	Evaluate CA (Observational cohort study)	Questionnaires	Target group (indigenous patients)	23,235	35.1 (13.4)	77.2	All levels	Australia	66.7
Ünlü Ince (2013)^94^	Quantitative	Evaluate CA (RCT with FU: 1, 4 m)	Questionnaires	Target group (immigrants)	96	35.2 (9.3)	61.5	All levels	The Netherlands	83.3
Vöhringer (2020)^144^	Quantitative	Evaluate CA (single study)	Questionnaires	Target group (community members)	386	26.0 (6.0)	69.4	All levels	Germany	81.0
Wagner (2012)^95^	Quantitative	Evaluate CA (pilot study with FU: 5 w)	Questionnaires	Target group (community members)	15	29.3 (7.1)	86.7	All levels	Iraq	71.4
Wang (2013)^88^	Quantitative	Evaluate CA (RCT with FU: 1, 2, 3 m)	Interviews, questionnaires	Target group (community members)	183	Range: 18–70	78.1	All levels	China	88.1
Wasil (2020)^83^	Qualitative; planned: quantitative	Inform CA; planned: evaluate CA (RCT with FU)	Focus groups, questionnaires	School staff, target group (adolescents)	21	–	–	All levels	India	47.6
Yokomitsu (2020)^85^	Planned: quantitative	Planned: evaluate CA (RCT with FU: 8, 12 w)	Questionnaires	Target group (students)	–	–	–	–	Japan	75.8

*M* mean, *SD* standard deviation, *RCT* randomised controlled trial, *FU* follow-up assessment, *m* months, *w* weeks.

^a^Grouped according to the study design: qualitative studies aimed to collect information on needed cultural adaptations; quantitative studies aimed to evaluate culturally adapted interventions.

^b^Overall percentage of quality assessment analysis with the Quality Assessment Tool for Reviewing Studies with Diverse Designs^120^.

### Quality assessment

The initial interrater reliability between the two independent raters was *κ* = 0.62, which can be considered a substantial agreement^86^. Overall, the quality of the included articles revealed an average quality score of 75.4%. The assessed quality score ranged from 35.7 (ref. ^87^) to 95.2% (ref. ^63^). Nearly all articles provided an explicit theoretical framework (93.9% of the maximum score), named the aims of their conducted or planned study (95.8%), and described their research setting (93.9%) and their procedure for data collection (90.3%) in a satisfactory way. Often, there was only poor evidence of the sample size (40.1%), and users were rarely involved in the study design (42.4%).

Table 1 presents the individual quality score of every article and Supplementary Table 4 contains the comprehensive ratings of the two independent reviewers.

### Components of cultural adaptation

**Fig. 2:**
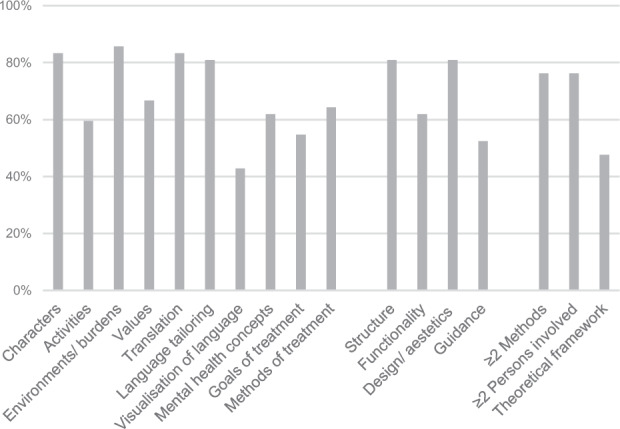
Taxonomy of cultural adaption of internet- and mobile-based interventions (IMI). Percentages of the 42 included IMI that fulfilled the respective content, methodological, and procedural components of the taxonomy.

Various components of which the authors reported the cultural adaptation were identified across the included articles and grouped in distinguishable aspects of culturally adapting IMI. Using the frameworks of previous literature as a base^47,54,74–76^, the classification had been continuously refined in the course of data synthesis, resulting in ten content, four methodological, and three procedural components that provided qualitative information on the cultural adaptation of IMI for mental disorders. Generally, the consideration of these components was used by the authors to enhance the fit of the IMI to the context, lives, and burdens of the new target group. Across all interventions averaged consideration of content (68.8%) versus methodological (69.0%) versus procedural (66.7%) components was comparable with 68.5% of all components being considered. The 17 components of culturally adapting IMI that were identified across the articles are illustrated in Table 2, including specific adaptation examples of the articles. A more detailed illustration of the conducted adaptations of each IMI can be found in Supplementary Table 5. An overview of the 17 components illustrating the respective extent of consideration across the articles is provided in Fig. 2.

**Table 2:** Taxonomy of the 17 identified components of cultural adaptation of internet- and mobile-based interventions for mental disorders.

Core components	Specific components^a^	Example^a^
*Content components*		
1. Illustrated characters		
	Appearances/names of characters	Change of names of characters to popular Chinese names (e.g., Lindsay to Xiaoli)^132^
	Content/stories/background of characters	Added character of a young woman who migrated two years ago and can’t find her way in the Netherlands^123^
	Role of the characters	Change of the narrator characteristics: no lecturing expert but an abuela sitting in her living room^100,101,140^
2. Illustrated activities		
	Daily life	Rather social activities than individual activities^135^
	Gender-specific behaviour	Gender separation in daily life^97–99^
	Religious/traditional activities	Praying instead of walking with dog, having a cocktail^71^
	Coping strategies	Added culturally relevant resources^105,133^
3. Illustrated environment/burdens		
	Mental health	Low medical infrastructure due to war^61,95,144^
	Countries, politics	Eventually adding information about good habits when living in Sweden, e.g., eating^63,134^
	Education	Low socio-economic status, low level of parents education^139^
	Burdens	High level of pressure for academic excellence as stressor for adolescents, lack of siblings could lead to decreased social skills, loneliness^136^
4. Illustrated values/traditions		
	Handling relationships/sexuality/marriage	Changing/excluding parts related to unmarried cohabitation^79^
	Value/importance of family/community	Include families and friends in the treatment goals to take into account possible consequences in the family and community that might be more relevant for participants than their own individual suffering^90^
	Value/importance of religion/respect/spirituality	Include a trustful relationship with God^97–99^
	Other values	Longing for their country^77^
5. Language translation		
	Translating intervention	English to Chinese^88^
	Translating to dialect	Rephrasing in Canadian English^131^
	Providing various language options to choose	Offer more language options (supply native language support for all important services)^111^
6. Language tailoring		
	Simplify text: shortening text passages, simplifying sentences	Less technical phrasing, modify wording for easier readability^87^
	Use of concrete terms or informal language	The polite German form (i.e., ‘Sie’) has been exchanged for the colloquial form (i.e., ‘Du’)^78^
	Milder descriptions of mental health concepts	Describing psychological problems in terms of idioms of distress^94^
7. Visualisation of language		
	Use of metaphors	Metaphors that appeal better and depict distress and recovery^69,89^
	Use of verbal expressions (sayings, quotes)	Cultural specific terms like ‘ciclovía’ (bike rides on sundays)^73,138^
	Use of symbols	The Local name selected for the programme—Selge—has two meanings: clear and sober^82^
8. Difference in concepts of mental health and its treatment		
	Poor knowledge	Rather somatic symptoms (e.g., fatigue, decreased energy, weight changes)^123^
	Stigmatisation of mental health problems	Framing the goal of the intervention as a stress management tool or academic performance tool instead of mental health intervention, in order to reduce the stigma^141^
	Distrust in treatment/confidentiality	Increase trust in the app: lessen worries about data protection or doubts whether the programme would actually help^111^
	Related to religion/supernatural powers	Fatalistic assumption that suffering is given by fate or by God and has to be endured^90^
9. Goals of treatment		
	Increase understanding/acceptance of mental disorders	Addressing myths about depression and its treatment^125^
	Increase understanding on treatment possibilities	Informing on PTSD and its management in stressful environments^87^
	Enhance coping strategies	Improvement of parent-adolescent communication to enhance protective factor of family^139^
10. Methods of treatment		
	Comprehensive psycho-education	Include educational videos by members of the American Indian/Alaska Native community^130^
	Information/links to other helpful addresses	Adapt Resources (information materials, services available, technologies, e.g., links to webpages or informative manuals)^143^
	Emphasis on positive outcomes/recovery	Deemphasise focus on mental health, e.g., help the user to have a good sleep instead of to help the user cope with sleeping problems^128^
*Methodological components*		
11. Structure		
	Shorten intervention	Content was restructured to 20 (instead of 40) short sessions^96^
	Eliminate repeating content	Less repetitions^93,142^
	Changes in texts	Include storytelling instead of ‘academic’ presentation^92^
	Simplify introduction	Clear and precise instructions^70,124^
	Add optional intervention elements	Including traditional and spiritual healing as optional content^87^
12. Functionality		
	Provide more explanations	Less scientific explanations and illustrations for exercises^91^
	Simplify navigation	Adapt the visual display of information to make it easy to navigate (e.g., illustrate contents in tables, flowcharts)^131^
	Access with low internet quality	Mobile app in addition to website^79^
13. Design and aesthetics		
	Adjust illustrations	Illustrate a logo designed by natives^129^
	User interface	Colour schemes were made brighter to be more reminiscent of the Japanese anime aesthetic^85^
14. Guidance		
	Person used as guide	Guidance by a Chinese therapist^81,106^
	Format of guidance (tailored feedback)	Participants can ask for personal contact in addition to automatic feedback^137^
	Amount of guidance	Choice of time of contact^69,89^
*Procedural components*		
15. Methods used to obtain information		
	Personal interaction (focus groups, interviews, discussions, think aloud)	Gain feedback in with qualitative data for the process evaluation and further implementation of the programme^80^
	Surveys/questionnaires	Assessed acceptance and effectiveness^126^
	Pilot/feasibility studies	Feasibility study with Columbian college students^73,138^
16. Persons involved		
	Target group and associated people	Georgian patients^84^
	Professionals working with the target group	Indian high school teachers and administrators, Indian college students, Indian researchers^83^
	Professionals not working with the target group	Professional translators^126^ or professional illustrators^141^
17. Theoretical framework		
	Guideline for cultural adaptation of face-to-face treatment	Adaptation in Eylem et al.^127^ based on the Ecological validity and cultural sensitivity framework^47^

^a^The specific components and examples represent an overview of the composition of the core components. A more detailed illustration is given in Supplementary Table 5.

Regarding content components, (1) an important element to adapt were the characters illustrated in the IMI (adapted in 35 of 42 interventions). (2) Alongside the characters, activities were added, removed, or tailored to make them relevant to the target group (25/42 IMI). (3) Furthermore, environments, settings, and, associated with this, specific burdens illustrated in the IMI were adapted (36/42 IMI). (4) Related to these aspects, researchers changed the content to accommodate culturally relevant values and traditions (28/42 IMI). Beside the adaptation of the daily life illustrated in the IMI, language adaptation was considered important: (5) numerous studies translated the entire intervention into the main language or the regional dialect of the target group (35/42 IMI). In addition, language and texts were (6) tailored to the target group (34/42 IMI), and (7) visualised with appropriate quotes, symbols, and metaphors (18/42 IMI). (8) When culturally adapting their IMI, researchers also considered potential differences in the target groups’ concepts of mental health and its treatment (26/42 IMI). Correspondingly, (9) the aims (23/42 IMI) and (10) the used treatment methods in the IMI (27/42 IMI) were changed occasionally.

Regarding methodological components of cultural adaptation, (11) the general structure of IMI was adapted (34/42 IMI). (12) Also, researchers adapted their IMI to enhance its functionality and ease of use for the specific target group (26/42 IMI), e.g., by including interactive elements or simplifying the navigation. (13) Moreover, the design and the aesthetics were adjusted (34/42 IMI). (14) As another aspect of culturally adapting IMI, researchers have changed the format or amount of guidance (22/42 IMI).

With regard to the procedure of cultural adaptation, (15) researchers used a wide range of methods to obtain information about necessary cultural adaptations of their IMI and about the adequacy of the conducted adaptations (32/42 IMI used at least two different methods). (16) During the cultural adaptation process, different people were involved, including healthcare professionals or the target group itself (32/42 IMI involved at least two groups of people). (17) Many authors also based their adaptation process on theoretical frameworks for cultural adaptations of face-to-face therapies (20/42 IMI).

### Extent of cultural adaptation

The consideration of each of the 17 components of cultural adaptation of IMI was evaluated across the articles to illustrate the extent of cultural adaptation for each intervention. The points were added up (0–17 points), revealing an average extent of cultural adaptation of *M* = 11.6 (SD = 3.7), with the smallest extent being three (17.7%)^88^ and the highest being 17 points (100%)^69,89–91^ for the respective interventions. A list of articles considering the respective components is provided in Table 3.

**Table 3:** Overview of the components used in the included articles during the cultural adaptation of their internet-and mobile-based based interventions.

	Content components										Methodological components				Procedural components				
	Illustrated				Language			Treatment										Points/study	%/study
First author (year)	Char	Act	Env	Val	Tran	Tail	Vis	Con	Goal	Meth	Stru	Fun	Des	Guid	Inf	Per	Fram		
Abi Ramia (2018)^69^/																			
Harper Shehadeh (2020)^89^	✓	✓	✓	✓	✓	✓	✓	✓	✓	✓	✓	✓	✓	✓	✓	✓	✓	17	100.0
Abuwalla (2017)^123^	✓	✓	✓	✓	✓		✓	✓	✓	✓	✓	✓	✓	✓	✓		✓	15	88.2
Arjadi (2018)^62,70^	✓	✓		✓	✓	✓		✓	✓	✓	✓	✓	✓	✓	✓	✓	✓	15	88.2
Bolinski (2018)^78^	✓		✓		✓	✓	✓				✓		✓					7	41.2
Brooks (2013)^87^			✓	✓		✓		✓	✓	✓	✓	✓		✓	✓	✓	✓	12	70.6
Burchert (2018)^111^	✓		✓		✓	✓		✓	✓	✓	✓	✓	✓	✓	✓			12	70.6
Campbell (2015)^92^	✓	✓	✓			✓	✓	✓		✓	✓		✓		✓			10	58.8
Chen (2020)^81^/																			
Lin (2020)^106^		✓	✓	✓	✓	✓	✓						✓	✓	✓	✓		10	58.8
Choi (2012)^125^	✓	✓		✓	✓	✓		✓	✓				✓			✓		9	52.9
DaPonte (2018)^126^	✓		✓		✓									✓				4	23.5
Eylem (2021)^127^	✓		✓	✓	✓	✓	✓	✓	✓	✓	✓	✓	✓	✓	✓	✓	✓	16	94.1
Garabiles (2019)^128^	✓	✓	✓	✓	✓	✓	✓	✓	✓	✓	✓	✓	✓		✓	✓	✓	16	94.1
Gorman (2013)^129^	✓	✓	✓	✓		✓		✓	✓	✓	✓	✓	✓	✓	✓	✓		14	82.4
Hiratsuka (2019)^130^	✓	✓	✓	✓		✓		✓	✓	✓	✓	✓	✓		✓	✓		13	76.5
Imamura (2019)^80^	✓		✓		✓					✓	✓	✓	✓		✓	✓		9	52.9
Ip (2016)^93^/																			
Sobowale (2013)^142^		✓	✓	✓	✓	✓		✓	✓	✓	✓	✓	✓	✓	✓	✓	✓	15	88.2
Juniar (2019)^71^	✓	✓	✓		✓	✓	✓	✓			✓	✓	✓	✓	✓	✓	✓	14	82.4
Kaal (2020)^82^	✓				✓	✓	✓			✓	✓		✓		✓	✓		9	52.9
Kanuri (2020)^96^	✓		✓			✓			✓		✓	✓			✓	✓		8	47.1
Kayrouz (2015/2016)^97–99^	✓	✓	✓	✓	✓	✓		✓	✓				✓		✓	✓		11	64.7
Knaevelsrud (2015)^61^/																			
Vöhringer (2020)^144^/																			
Wagner (2012)^95^			✓	✓	✓		✓	✓	✓					✓				7	41.2
Lal (2020)^131^		✓	✓		✓	✓				✓	✓	✓	✓		✓	✓	✓	11	64.7
Lindegaard (2020)^63^/																			
Nygren (2018)^134^	✓		✓	✓	✓	✓		✓	✓	✓	✓	✓	✓	✓	✓	✓	✓	15	88.2
Luo (2021)^132^	✓		✓		✓	✓				✓			✓	✓	✓	✓		9	52.9
Muroff (2017/2019)^105,133^	✓	✓	✓	✓	✓	✓	✓	✓	✓	✓	✓	✓				✓		13	76.5
Nygren (2019)^135^	✓	✓	✓	✓	✓			✓		✓	✓	✓	✓	✓				11	64.7
Okujava (2019)^84^	✓		✓	✓	✓	✓	✓			✓		✓	✓	✓	✓	✓		12	70.6
Paris (2018)^100^/																			
Silva (in prep)^140^/																			
Silva (2020)^101^	✓		✓	✓	✓	✓	✓	✓	✓		✓	✓	✓		✓	✓	✓	14	82.4
Patel (2016)^136^	✓		✓	✓	✓	✓			✓		✓	✓	✓		✓	✓	✓	12	70.6
Pinto-Bruno (2019)^137^	✓	✓			✓									✓			✓	5	29.4
Rahmadiana (2019)^79^	✓	✓	✓	✓	✓	✓		✓		✓	✓	✓	✓		✓	✓	✓	14	82.4
Salamanca-Sanabria (2019/2020)^73,138^	✓	✓	✓	✓	✓	✓	✓	✓	✓	✓	✓		✓	✓	✓	✓	✓	16	94.1
Saulsberry (2013)^139^	✓	✓	✓	✓	✓	✓		✓	✓		✓	✓	✓	✓	✓	✓	✓	15	88.2
Shala (2020)^90^	✓	✓	✓	✓	✓	✓	✓	✓	✓	✓	✓	✓	✓	✓	✓	✓	✓	17	100.0
Sit (2020)^141^	✓	✓	✓	✓	✓	✓	✓	✓		✓	✓		✓		✓	✓	✓	14	82.4
Spanhel (2019)^91^	✓	✓	✓	✓	✓	✓	✓	✓	✓	✓	✓	✓	✓	✓	✓	✓	✓	17	100.0
Teles (2020)^143^	✓	✓	✓	✓	✓	✓	✓			✓	✓	✓	✓	✓	✓	✓	✓	15	88.2
Titov (2018)^77^		✓	✓	✓					✓		✓				✓	✓		7	41.2
Ünlü Ince (2013)^94^	✓		✓		✓	✓		✓			✓					✓		7	41.2
Wang (2013)^88^					✓						✓		✓					3	17.7
Wasil (2020)^83^	✓	✓	✓	✓		✓				✓	✓		✓		✓	✓		10	58.8
Yokomitsu (2020)^85^	✓				✓	✓				✓	✓	✓	✓					7	41.2
Total																		11.6	68.2

*Char* illustrated characters, *Act* illustrated activities, *Env* illustrated places/environment/burdens, *Val* illustrated values/traditions, *Tran* language translation, *Tail* language tailoring, *Vis* visualisation of language, *Con* mental health (treatment) concepts, *Goal* goals of treatment, *Meth* methods of treatment, *Stru* structure, *Fun* functionality, *Des* design/aesthetics, *Guid* guidance, *Inf* ≥ 2 methods to obtain information, *Per* ≥ 2 persons involved in CA, *Fram* theoretical framework for CA.

### Adherence and effectiveness of the culturally adapted interventions

Characteristics of those articles providing quantitative data, and their adherence and effectiveness outcomes are illustrated in Table 4, including the measures of the respective non-adapted interventions, if available.

**Table 4:** Adherence and effectiveness outcomes of the articles included in the quantitative analyses.

First author (year)	Study type	*N* (IG)	*N* (CG)	ITT-analysis	Pre-/post-period	Control group	Target mental disorder	Primary outcome	Effect size (Hedges’ *g*) adapted IMI original IMI^a^	% completers adapted IMI original IMI^a^	% completed modules adapted IMI original IMI^a^
Arjadi (2018)^62^	RCT	159	154	Yes	10 w	Psycho-education	Depressive symptoms	PHQ-9	0.39	57.9% completed ≥5/8 modules	62.5%
									–	–	–
Campbell (2015)^92^	Mixed method study	40	–	–	9 w	–	Substance abuse	–	–	55% completed ≥17/32 modules	58.1%
									–	–	–
Chen (2020)^81^	Controlled study	183	72	Yes	8 w	Self-guided (waitlist)	Social anxiety symptoms	SIAS	Guided-waitlist: 1.03^145^^b^	–	51.3%
									0.84^146^	51.6%^146^	77.4%^146^
Choi (2012)^125^	RCT	25	30	Yes	9 w	Waitlist	Depressive symptoms	BDI	0.91	64%	82.7%
									0.63^147^	74.1%^147^	88.8%^147^
								PHQ-9	0.49		
									0.86^147^		
DaPonte (2018)^126^	Single group study	31	–	Yes	8 w	–	Depressive + anxiety symptoms	PHQ-9	2.92^c^	74.2%	85.8%
									0.58^148^	91.9%^148^	91.9%^148^
								GAD-7	3.29^c^		
									0.52^148^		
Eylem (2021)^127^	RCT	10	8	No	6 w	Psycho-education, waitlist	Suicidal ideation	BSS	0.17	80%	93.3%
									0.28^149^^c^	21.6%^149^	–
Harper Shehadeh (2020)^89^	Mixed method study	26	–	No	9 w	–	Depressive symptoms	PHQ-8	1.56^c^	24.8%	–
									–	–	–
Hiratsuka (2019)^130^	Single group study	24	–	No	12 w	–	Post-traumatic stress symptoms	–	–	33.3% used website frequently	36.5%
								PCL-L	0.14^150^	82.3%^150^	–
Ip (2016)^93^	RCT	130	127	Yes	8 m	Psycho-education	Depressive symptoms	CES-D	0.22	10.1%	33.5%
									0.74^151^^c^	96.3%^151^	–
Kanuri (2020)^96^	Single group study	15	–	No	4 w	–	Anxiety symptoms	–	–	0%	31.8%
									–	–	–
Kayrouz (2015)^97^	Single group study	11	–	Yes	8 w	–	Depressive + anxiety symptoms	PHQ-9	1.74^c^	91%	92.8%
									0.58^148^	91.9%^148^	91.9%^148^
								GAD-7	1.08^c^		
									0.52^148^		
Kayrouz (2016)^98^	Single group study	13	–	Yes	8 w	–	Depressive + anxiety symptoms	PHQ-9	1.39^c^	77%	87.6%
									0.58^148^	91.9%^148^	91.9%^148^
								GAD-7	1.18^c^		
									0.52^148^		
Kayrouz (2016)^99^	Single group study	36	–	Yes	8 w	–	Depressive + anxiety symptoms	PHQ-9	1.20^c^	32.5%	52%
									0.58^148^	91.9%^148^	91.9%^148^
								GAD-7	1.15^c^		
									0.52^148^		
Knaevelsrud (2015)^61^	RCT	79	80	Yes	5 w	Waitlist	Post-traumatic stress symptoms	PDS	0.92	59.5%	–
								IES	1.28/1.39^152^	63.9%^152^	–
Lin (2020)^106^	RCT	55 (subgroups: 27; 28)	25 (subgroups: 14; 11)	Yes	2 m	Waitlist	Social anxiety symptoms	SIAS	2.7	47.3%	61.1%
									0.84^146^	51.6%^146^	77.4%^146^
Lindegaard (2020)^63^	RCT	30	29	Yes	8 w	Waitlist	Depressive symptoms	PHQ-9	0.85	6.7%	31.9%
									–	–	–
Luo (2021)^132^	RCT	191	181	Yes	6 w	Education brochure	Eating disorder	BDS	0.40	81.7%	92.3%
									0.76^153^	89.5%^153^	95.6%^153^
Muroff (2019)^105^	Comparison study	58	21	No	6 m	–	Drug + alcohol use	ASI-6	–	73.4% remained active for 4 m	87.9%
									–	78.2% remained active for 4 m^154^	–
Nygren (2019)^135^	RCT	25	25	Yes	8 w	Waitlist	Depressive symptoms	BDI-II	1.27	36%	62.9%
									0.96^155^	65%^155^	74.0%^155^
Okujava (2019)^84^	Single group study	52	–	No	~10 w	Treatment as usual	Insomnia	ISI	–	48.1%	–
								PSQI	1.06^156^	72.9%^156^	~94.2%^156^
Paris (2018)^100^	RCT	43	49	Yes	8 w	Treatment as usual	Substance use	Days of primary substance use/week	*d* = 0.15^d^	56%	75.7%
									*d* = 0.71^157^^e^	50.0%^157^	–
Salamanca-Sanabria (2020)^138^	RCT	107	107	No	7 w	Waitlist	Depressive symptoms	PHQ-9	0.91	9.3%	37.7%
									0.64^158^	38%^158^	60.6%^158^
Silva (2020)^101^^f^	RCT	39	44	Yes	8 w	Treatment as usual	Substance use	DSM-5 alcohol use disorder criteria	*d* = 1.12^g^	–	–
									–	50.0%^157^	–
Titov (2018)^77^	observational cohort study	780	22,455	No	–	Non-indigenous	General psychological distress	K-10	1.57^c^	70.0%	–
									1.42^c^ (non-indigenous)	71.9% (non-indigenous)	–
Ünlü Ince (2013)^94^	RCT	49	47	Yes	6 w	Waitlist	Depressive symptoms	CES-D	0.37	20%	~20.4%
									0.50^159^	55.1%^159^	70.0%^159^
Vöhringer (2020)^144^	Single group study	386	–	No	–	–	Post-traumatic stress symptoms	–	–	62.7% completed ≥4/6 modules	–
								IES	1.28/1.39^152^	63.9%^152^	–
Wagner (2012)^95^	Single group study	15	–	No	5 w	–	Post-traumatic stress symptoms	PDS	1.57^c^	100%	100% (only completers were included)
								IES	1.28/1.39^152^	63.9%^152^	–
Wang (2013)^88^	RCT	Urban: 46 Rural: 49	Urban: 44 Rural: 44	Yes	1 m	Waitlist	Post-traumatic stress symptoms	PDS	Urban: 0.79 Rural: 0.54	–	–
									0.43^160^	–	–

*IG* intervention group, *CG* control group, *ITT* intention-to-treat, *IMI* internet- and mobile-based intervention, *% completers* percentage of completers of the respective intervention, *% completed modules* percentage of the completed modules, *RCT* randomised controlled trial, *w* weeks, *m* months, *PHQ* Patient Health Questionnaire^102^, *BDI* Beck’s Depression Inventory^161^, *GAD* Generalised Anxiety Disorder^103^, *BSS* Beck’s Scale for Suicide Ideation^162^, *PCL-C* PTSD Checklist—Civilian Version^163^, *CES-D* Centre for Epidemiologic Studies Depression Scale^164^, *PDS* Post-traumatic Diagnostic Scale^104^, *IES* Impact of Event Scale^165^, *SIAS* Social Interaction Anxiety Scale^166^*, BDS* Body Dissatisfaction Scale^167^, *ASI* Addiction Severity Index^168^, *ISI* Insomnia Severity Index^169^, *PSQI* Pittsburgh Sleep Quality Index^170^, *K-10* Kessler Ten-Item Scale^171^.

^a^If available, comparable outcomes of the original IMI are illustrated.

^b^Original effectiveness analyses are presented in Kishimoto et al.^145^, which was not included in data extraction because of not being an original article.

^c^Comparison of pre- and post-treatment assessments.

^d^Calculated using the *t* value due to a lack of reported means and standard deviations.

^e^Value reported in the original article, due to a lack of reported means and standard deviations.

^f^A sub-sample of the Paris et al.^100^ study was re-analysed.

^g^Calculated using the *χ*^2^-value due to a lack of reported means and standard deviations.

The interventions varied widely in their length (range: 1 (ref. ^83^)–32 (ref. ^92^) modules) and duration (range: 1 day^83^–8 months^93^). The average percentage of completed sessions of the culturally adapted IMI was 63.7% (CI: 52.3–75.1%, *n* = 21 interventions), ranging from 20.4% (ref. ^94^) to 100% (ref. ^95^). The average percentage of participants who completed all sessions of the adapted IMI was 50.6% (CI: 36.8–64.3%, *n* = 21 interventions), ranging from 0% (ref. ^96^) to 100% (ref. ^95^).

**Fig. 3:**
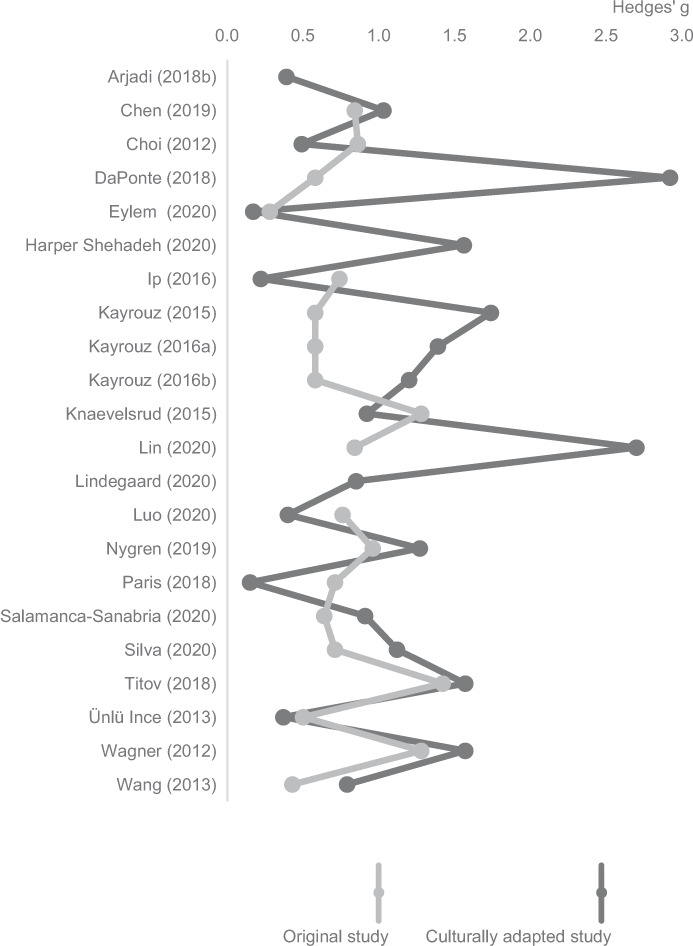
Effect sizes (Hedges’ *g*) of the culturally adapted versus original internet- and mobile-based interventions. Twenty-two included articles provided relevant information on the effect of their intervention. If more than one primary outcome was reported, the effect size of the Patient Health Questionnaire^102^ is illustrated. Both single group studies and randomised controlled trials are included. Full information on the respective effect sizes can be found in Table 4.

Eight single group studies reported on the effectiveness of their IMI comparing mental disorder symptoms of the participants between pre- and post-treatment assessments, three of which assessed the effectiveness of the same intervention^97–99^. Fourteen (randomised) controlled trials (Chen et al.^81^ stopped randomisation) reported on the effectiveness of their IMI comparing mental disorder symptoms of the participants who had access to an adapted IMI to those who had no access (control group receiving delayed access, no access, or psychoeducational information). Outcomes of one randomised controlled trial were published in two articles^100,101^. Primary outcomes varied between the studies, with the Patient Health Questionnaire^102^ being the scale most used (*n* = 9), followed by the General Anxiety Disorder Scale^103^ (*n* = 4) and the Post-traumatic Disorder Scale^104^ (*n* = 3). Similarly, the time between pre- and post-assessments differed between the studies, ranging from 4 weeks^96^ to 6 months^105^. Effect sizes of randomised controlled trials ranged from Hedges' *g* = 0.15 (ref. ^100^) to 2.70 (ref. ^106^; see Fig. 3).

### Post hoc analyses

Correlation analyses did not reveal any significant links between the intervention characteristics (amount of modules, provided guidance) and the extent of (specific components of) cultural adaptation, *r*(40) ≤ 0.12, *p* ≥ 0.330. The extent of culturally adapting content components significantly differed between the target groups of the IMI (people living in LMIC, in high-income countries, immigrants, indigenous people), *F*(3,38) = 6.09, *p* = 0.002, with pairwise comparisons suggesting a lower extent of content cultural adaptation for people from high-income countries, as compared to migrants and people living in LMIC. No link was revealed between the amount of modules, guidance, or extent of cultural adaptation and the effectiveness (*r*(13) ≤ 0.20, *p* ≥ 0.487) or adherence of the IMI (*r*(9) ≤ −0.35, *p* ≥ 0.287). Nor did the effectiveness (*F*(2,12) = 0.39, *p* = 0.683) or adherence (*F*(2,8) = 0.10, *p* = 0.909) of the IMI significantly differ between the target groups of the IMI. Detailed results of the post hoc analyses can be found in Supplementary Table 6.

## Discussion

This is a systematic review synthesising the components and procedures used in the cultural adaptation of IMI for mental disorders. The proposed taxonomy comprises 17 components, covering the key domains of content (e.g., illustrated daily life and mental health concepts), methodological (e.g., structure and design), and procedural components (e.g., used methods and involved persons).

The 55 articles included in this review explored, conducted, and reported on the cultural adaptation of 42 IMI that were originally used for a culturally differing target group. Cultural adaptations were done to illustrate relatable situations (e.g., persons, activities), to fit the characteristics of the communities (e.g., collectivism, values, traditions), to consider specific risk factors for low mental health (e.g., burdens due to migration, war, discrimination, and low socio-economic status), and to address health-related problems (e.g., low mental health literacy and corresponding distrust, risky behaviour, or limited access to treatment). Twenty-eight studies, including 14 (randomised) controlled trials, evaluated the culturally adapted IMI in terms of adherence and/or effectiveness for the new target group. Adherence rates and effect sizes of randomised controlled trials seem to be comparable to both those found in the studies on the respective original IMI (see Table 4), and those found in studies investigating the adherence and effectiveness of IMI in general^36,107^. However, enrolment and adherence rates of IMI are generally low^108–110^. Above that, we could not identify any study on a direct comparison of a culturally adapted with a non-adapted IMI. Hence, we believe it is premature to conclude that cultural adaptation is a prerequisite to make IMI work in people living in LMIC, migrants, or indigenous people.

Our taxonomy of cultural adaptation of IMI may provide a base for future researchers to systematically adapt IMI by considering the 17 identified components. The included ten content components overlap with components that are culturally adapted in face-to-face psychological treatments^47^. Thus, both surface (e.g., the adaptation of characters, activities, and language) and deep (e.g., the adaptation of burdens, values, mental health concepts, and goals and methods of treatment) structure adaptations were shown to be important^50^. Also, the three procedural components seem to reflect the procedure proposed in the adaptation of face-to-face psychological treatments^54^. Accordingly, the study investigators used multiple methods to obtain information from various groups of people (e.g., mental health experts, target group) on necessary adaptations of the IMI, including focus groups and feasibility trials. Thereby, about half of the IMI were adapted following theoretical frameworks of cultural adaptation of face-to-face treatments. However, in addition to the content and procedural components, we identified four unique methodological components specifically considered in the cultural adaptation of IMI. These include components related to the structure (e.g., shortened text sequences or modules), functionality (e.g., the consideration of a potentially bad internet connection), design and aesthetics (e.g., the amount or design of pictures), and human guidance (e.g., level of guidance). Hence, it may not be sufficient to rely on existing frameworks for cultural adaptation of face-to-face treatments when culturally adapting IMI, as also highlighted by Lal et al.^72^ and implemented by Burchert et al.^111^.

By enabling a systematic cultural adaptation of IMI, our taxonomy on cultural adaptation of IMI can furthermore serve as a base to measure the extent and type of cultural adaptation, and thus can lay an initial foundation for further examining the relevance and necessity of the cultural adaptation of (specific elements of) IMI. This relevance and necessity of cultural adaptation could differ, for example, depending on the target group, the addressed disorder, or the language of the IMI. Conducted exploratory post hoc analyses indicate that culturally adapting IMI for people with a migrant background and people living in LMIC might be more important as compared to culturally adapting IMI for new target groups in western countries, especially with respect to content components. However, in the current systematic review, no link between the extent of the conducted cultural adaptations, and the effectiveness or adherence of the IMI was revealed. The analyses should be interpreted with caution, as the present systematic review included very heterogeneous IMI and did not yield sufficient outcome data. Even with more randomised controlled trials included in meta-analyses, the heterogeneity of interventions studied remains mostly very high, making it difficult to draw conclusions on the influence of specific intervention characteristics or components of cultural adaptation on the effectiveness of the IMI. Thus, questions on possible differences in the necessity of cultural adaptation and its influence on the effectiveness of IMI could be addressed in other study designs. By using randomised factorial trials^112,113^, different versions of the same IMI that were systematically culturally adapted to varying degrees could be tested against each other. For example, deep structure content components versus surface structure content components versus no content components of an IMI could be culturally adapted. The effectiveness of each of the versions could then be evaluated and compared, herewith enabling to draw conclusions on the benefit of culturally adapting these specific components.

Some limitations of this systematic review need to be considered. First, we excluded studies that did not report (*n* = 14) or provide details on (*n* = 6) their cultural adaptation. At the same time, we excluded grey literature with potential unpublished reports on conducted cultural adaptation. This might have led to biased findings and hindered us from drawing conclusions on a potential influence of the (extent of) cultural adaptation of IMI on the adherence or effectiveness of the respective IMI. Second, we also excluded studies that newly developed culturally sensitive IMI for a target group of people living in LMIC, migrants, or indigenous people (*n* = 32), in contrast to studies that culturally adapted previously used and evaluated IMI. Yet, the 17 components of the taxonomy of cultural adaptation might also be helpful in the development of culturally sensitive IMI. Thus, it may be a fruitful next step to conduct a review on the development of culturally sensitive IMI and compare the results with the current review. Third, the wide range of target groups in our review makes it difficult to interpret the findings across all included target groups. Following the cultural diversity approach^114^, the necessary extent and depth of culturally adapting IMI may differ between target groups, also depending on the similarities and differences between the original and new target groups. Fourth, although we extracted and summarised aspects of cultural adaptation that the authors have reported to be adapted in order to enhance the cultural fit, we cannot fully rule out that there might have been other reasons (e.g., resources of the researchers and technical innovations). Fifth, the choice of the two cultural adaptation models used as a basis for data extraction^47,54^ was not based on a systematic approach, but mainly on the frequent use of the models in previous research. Sixth, as the extracted components of cultural adaptation represent differing intensities of adaptation (surface structure adaptations, such as language translation, versus deep structure adaptations, such as the consideration of differences in the concepts of mental health and its treatment), the extent to which the components are considered is not equal to the extensiveness of cultural adaptation. Seventh, reliability regarding data extraction and data synthesis was not evaluated and may thus be limited.

## Conclusions

The mental health treatment gap for people in LMIC and for migrants or indigenous people in high-income countries needs to be addressed with innovative and scalable approaches^5^. To provide impactful interventions, an efficient and systematic cultural adaptation is central^24^. The present systematic review illustrates a taxonomy of 17 components of culturally adapting IMI, making them more relatable to the new target group by considering their specific context, burdens, and understanding of mental health. This taxonomy can serve as a base for the systematic cultural adaptation of IMI. The next steps should be to investigate the relevance and necessity of a systematic cultural adaptation of IMI, which could help provide adequate and effective mental healthcare to underserved populations, herewith contributing to reduce global public mental health inequalities.

## Methods

This systematic review is reported according to the Preferred Reporting Items for Systematic reviews and Meta-Analyses, the PRISMA guidelines (see Supplementary Table 1 for PRISMA checklist)^115^. A systematic review protocol has been published^116^ and registered in the International Prospective Register of Systematic Reviews of the National Institute for Health Research (PROSPERO; registration number: CRD42019142320).

### Eligibility criteria

We included (a) peer-reviewed journal articles that reported on an intervention that (b) was primarily provided in an internet-, computer-, app-, or mobile-based setting (with or without human support; pure videoconferencing interventions were excluded), (c) used psychological methods to address mental disorders or disturbances, and (d) was adapted from a previously used and evaluated IMI to fit a new and culturally differing target group. Interventions newly developed for a culturally differing target group were excluded due to assumed differences in the procedure and content of cultural adaptation versus culturally sensitive development of IMI. Psychological interventions were defined according to Kampling et al. and include, among other psychological-oriented interventions, cognitive behaviour therapy, psychodynamic psychotherapy, behaviour therapy or behaviour modification, systemic therapy, third-wave cognitive behaviour therapies, humanistic therapies, and integrative therapies^117^.

There were no restrictions in the participants’ age nor the language of the articles. Articles were translated to English, if necessary. Furthermore, study protocols were included, if they provided information on the cultural adaptation of the used interventions.

### Search strategy

A systematic literature search was conducted on October, 15th 2020 in EbscoHost/Medline, EbscoHost/PsycINFO, Ovid/Embase, Cochrane Central Register of Controlled Trials (CENTRAL), and Web of Science. The search covered two parallel reviews, with the second review including articles on the cultural adaptation of IMI for health promotion^118^. Keywords of the search term for the present review included a combination of terms and synonyms of (1) internet-based interventions, (2) mental health, and (3) cultural adaptation. The search was complemented by hand search, as well as backward and forward searches. The publication of studies described in an included study protocol was checked before the final data extraction. The full search term can be found in Supplementary Table 2.

### Study selection

The selection of articles was conducted in two steps using Covidence^119^. First, two independent reviewers screened titles and abstracts of all articles against the inclusion criteria. Second, the full-texts of seemingly relevant or ambiguous articles were screened against the inclusion criteria by two independent reviewers. Reasons for exclusion were documented. Disagreement was resolved in discussion, and, if no consensus could be reached, by consulting a third reviewer. The study selection process is illustrated in the PRISMA flow chart (see Fig. 1).

### Data extraction

Using a data extraction form in Covidence^119^, the following information was extracted from the selected articles: (a) study identification items, (b) sample characteristic, (c) study design characteristics, (d) study settings, (e) original intervention characteristics, (f) culturally adapted intervention characteristics, (g) details on content and methodological components of cultural adaptation, (h) details on procedural components of cultural adaptation, as well as (h) primary and secondary outcomes of the studies (adherence and effectiveness). The study authors were contacted if the variables of interest were not available or not clearly presented in the studies. All extracted data was double-checked by an independent reviewer.

### Quality assessment

Two independent reviewers assessed the Quality Assessment Tool for Reviewing Studies with Diverse Designs (QATSDD)^120^. The tool comprises 16 criteria that are applicable to diverse research designs, of which 14 apply to quantitative and to qualitative methods respectively. Thus, the QATSDD enabled an overall quality rating for included quantitative, qualitative, and mixed-methods studies. Beside items assessing completeness and clarity of reporting, further items can help rate the risk of bias of the studies (e.g., fit between stated research question and method of data collection, format and content of data collection tool, or method of analysis; good justification for analytical method selected). The criteria were rated on a four-point scale, producing a global score showing a low or high study quality. The quality score is presented as percentage of the highest possible points. Arising discrepancies between the two reviewers were resolved in discussion; if further needed, a third reviewer was consulted. Cohen’s Kappa was used to assess interrater agreement between low (0 and 1 point) and high (2 and 3 points) study quality^121^.

### Data synthesis

A qualitative synthesis of all included articles was conducted to describe the characteristics and findings of the studies. To answer the research questions, details on the cultural adaptation reported in the included studies were summarised and classified; only adaptations that the authors reported to have been made for cultural reasons were considered. The classification included content components, based on Bernal et al.^47^, methodological components, based on IMI evaluation frameworks^74–76^, and procedural components, based on Barrera and Castro^54^. We additionally rated which of the extracted components were culturally adapted versus not adapted in which intervention (one versus zero point), so to provide descriptive cultural adaptation scores to illustrate the extent of the conducted cultural adaptation for each of the interventions, and each of the components.

Findings related to the adherence to and the effectiveness of the culturally adapted IMI for mental disorders were summarised and descriptively illustrated. Adherence was evaluated using the percentage of completed modules (average number of completed modules divided by the number of all modules of the IMI). In addition, the percentage of study participants who completed all modules of the IMI was illustrated. The effectiveness of the intervention was evaluated using the effect size Hedges’ *g*^122^. In case of conducted randomised controlled trials, the effect size was calculated for the mean differences in the primary outcome(s) of the intervention and control groups at post-treatment assessments. In case of single group study designs, the effect size was calculated for the mean differences in the primary outcome(s) between pre- and post-treatment assessments. If available, adherence and effectiveness outcomes of the original IMI were illustrated in comparison to those of the culturally adapted IMI.

### Post hoc analyses

To exploratively investigate intervention characteristics that might influence the conducted cultural adaptation and the effectiveness and adherence of the adapted IMI, we conducted post hoc analyses. First, potential links between intervention characteristics (duration, provided guidance, and target group of the IMI) and the extent of cultural adaptation or the extent of specific components of cultural adaptation (content, methodological, and procedural) were analysed using Pearson’s correlation coefficients and an analysis of variance. Second, potential links between intervention characteristics or the extent of cultural adaptation and the effectiveness or the adherence (percentage of completers) of the interventions were analysed also using Pearson’s correlation coefficients or analyses of variance. Due to the explorative character of the analyses, no adjustments for multiple comparisons were applied.

## Supplementary information

Supplementary information

## Data Availability

The data that support the findings of this study are available from the corresponding author upon reasonable request.

## References

[1] World Health Organization. Mental health action plan 2013-2020 https://www.who.int/publications/i/item/9789241506021 (2013).

[2] Braveman P (2005). Health disparities and health equity: concepts and measurement. Annu. Rev. Public Health.

[3] Fiscella K, Franks P, Gold MR, Clancy CM (2003). Inequality in quality: addressing socioeconomic, racial, and ethnic disparities in health care. JAMA.

[4] Goesling B, Firebaugh G (2004). The trend in international health inequality. Popul. Dev. Rev..

[5] Patel V (2018). The Lancet Commission on global mental health and sustainable development. Lancet Comm..

[6] World Health Organization. (2004). Prevalence, severity, and unmet need for treatment of mental disorders in the World Health Organization world mental health surveys. J. Am. Med. Assoc..

[7] Patel V (2007). Mental health in low- and middle-income countries. Br. Med. Bull..

[8] Lamkaddem M (2014). Course of post-traumatic stress disorder and health care utilisation among resettled refugees in the Netherlands. BMC Psychiatry.

[9] Laban CJ, Gernaat HBPE, Komproe IH, Jong JTVM (2007). Prevalence and predictors of health service use among Iraqi asylum seekers in the Netherlands. Soc. Psychiatry Psychiatr. Epidemiol..

[10] Slewa-Younan S (2017). The mental health and help-seeking behaviour of resettled Afghan refugees in Australia. Int. J. Ment. Health Syst..

[11] Steel JL, Dunlavy AC, Harding CE, Theorell T (2017). The psychological consequences of pre-emigration trauma and post-migration stress in refugees and immigrants from. Afr. J. Immigr. Minor. Heal..

[12] Turrini G (2017). Common mental disorders in asylum seekers and refugees: umbrella review of prevalence and intervention studies. Int. J. Ment. Health Syst..

[13] Fazel M, Wheeler J, Danesh J (2005). Prevalence of serious mental disorder in 7000 refugees resettled in western countries: a systematic review. Lancet.

[14] Lindert J, Carta MG, Schäfer I, Mollica RF (2016). Refugees mental health - a public mental health challenge. Eur. J. Public Health.

[15] Kirmayer LJ, Brass GM, Tait CL (2000). The mental health of aboriginal peoples: transformations of identity and community. Can. J. Psychiatry.

[16] Nelson SE, Wilson K (2017). The mental health of Indigenous peoples in Canada: a critical review of research. Soc. Sci. Med..

[17] Marrone S (2007). Understanding barriers to health care: a review of disparities in health care services among indigenous populations. Int. J. Circumpolar Health.

[18] Eaton J (2011). Scale up of services for mental health in low-income and middle-income countries. Lancet.

[19] Patel V, Chowdhary N, Rahman A, Verdeli H (2011). Improving access to psychological treatments: lessons from developing countries. Behav. Res. Ther..

[20] Kiselev N (2020). Structural and socio-cultural barriers to accessing mental healthcare among Syrian refugees and asylum seekers in Switzerland. Eur. J. Psychotraumatol..

[21] Byrow Y, Pajak R, Specker P, Nickerson A (2020). Perceptions of mental health and perceived barriers to mental health help-seeking amongst refugees: a systematic review. Clin. Psychol. Rev..

[22] Sijbrandij M (2017). Strengthening mental health care systems for Syrian refugees in Europe and the Middle East: integrating scalable psychological interventions in eight countries. Eur. J. Psychotraumatol..

[23] Trilesnik B (2019). Implementing a need-adapted stepped-care model for mental health of refugees: preliminary data of the state-funded project ‘Refukey’. Front. Psychiatry.

[24] Holmes EA (2018). The Lancet Psychiatry Commission on psychological treatments research in tomorrow’s science. Lancet Psychiatry.

[25] Bockting CLH, Williams AD, Carswell K, Grech AE (2016). The potential of low-intensity and online interventions for depression in low- and middle-income countries. Glob. Ment. Heal.

[26] Bennett-Levy, J., Richards, D. A. & Farrand, P. in *Oxford Guide to Low Intensity CBT Interventions* (eds Bennett-Levy, J. et al.) 3–18 (Oxford University Press, 2010).

[27] Griffiths F, Lindenmeyer A, Powell J, Lowe P, Thorogood M (2006). Why are health care interventions delivered over the Internet? A systematic review of the published literature. J. Med. Internet Res..

[28] Carroll KM, Rounsaville BJ (2010). Computer-assisted therapy in psychiatry: be brave - it’s a new world. Curr. Psychiatry Rep..

[29] Moock J (2014). Support from the Internet for individuals with mental disorders: advantages and disadvantages of e-mental health service delivery. Front. Public Health.

[30] Andersson G, Titov N (2014). Advantages and limitations of Internet-based interventions for common mental disorders. World Psychiatry.

[31] Muñoz RF (2016). Massive open online interventions: a novel model for delivering behavioral- health services worldwide. Clin. Psychol. Sci..

[32] Mohr DC, Schueller SM, Araya R, Gureje O, Montague E (2014). Mental health technologies and the needs of cultural groups. Lancet Psychiatry.

[33] Waller R, Gilbody S (2009). Barriers to the uptake of computerized cognitive behavioural therapy: a systematic review of the quantitative and qualitative evidence. Psychol. Med..

[34] Sander L, Rausch L, Baumeister H (2016). Effectiveness of internet-based interventions for the prevention of mental disorders: a systematic review and meta-analysis. JMIR Ment. Heal.

[35] Andersson G, Titov N, Dear BF, Rozental A, Carlbring P (2019). Internet-delivered psychological treatments: from innovation to implementation. World Psychiatry.

[36] Karyotaki E (2021). Internet-based cognitive behavioral therapy for depression: a systematic review and individual patient data network meta-analysis. JAMA Psychiatry.

[37] Weisel KK (2019). Standalone smartphone apps for mental health—a systematic review and meta-analysis. npj Digit. Med..

[38] Rigabert A (2020). Effectiveness of online psychological and psychoeducational interventions to prevent depression: systematic review and meta-analysis of randomized controlled trials. Clin. Psychol. Rev..

[39] Karyotaki E (2018). Do guided internet-based interventions result in clinically relevant changes for patients with depression? An individual participant data meta-analysis. Clin. Psychol. Rev..

[40] Saraceno B (2007). Barriers to improvement of mental health services in low-income and middle-income countries. Lancet.

[41] Zayas LH, Torres LR, Malcolm J, DesRosiers FS (1996). Clinicians’ definitions of ethnically sensitive therapy. Prof. Psychol. Res. Pr..

[42] Gearing RE (2013). Adaptation and translation of mental health interventions in Middle Eastern Arab countries: a systematic review of barriers to and strategies for effective treatment implementation. Int. J. Soc. Psychiatry.

[43] Sue S, Zane N, Nagayama Hall GC, Berger LK (2009). The case for cultural competency in psychotherapeutic interventions. Annu. Rev. Psychol..

[44] Castro FG, Barrera M, Steiker LKH (2010). Issues and challenges in the design of culturally adapted evidence-based interventions. Annu. Rev. Clin. Psychol..

[45] Sangraula M (2021). Development of the mental health cultural adaptation and contextualization for implementation (mhCACI) procedure: a systematic framework to prepare evidence-based psychological interventions for scaling.. Glob. Ment. Health.

[46] Chu J, Leino A (2017). Advancement in the maturing science of cultural adaptations of evidence-based interventions. J. Consult. Clin. Psychol..

[47] Bernal G, Bonilla J, Bellido C (1995). Ecological validity and cultural sensitivity for outcome research - issues for the cultural-adaptation and development of psychosocial treatments with hispanics. J. Abnorm. Child Psychol..

[48] Rathod S, Phiri P, Naeem F (2019). An evidence-based framework to culturally adapt cognitive behaviour therapy. Cogn. Behav. Ther..

[49] Hwang WC (2006). The psychotherapy adaptation and modification framework: application to Asian Americans. Am. Psychol..

[50] Resnicow K, Baranowski T, Ahluwalia JS, Braithwaite R (1999). Cultural sensitivity in public health: defined and demystified. Ethn. Dis..

[51] Hinton DE, Jalal B (2014). Guidelines for the implementation of culturally sensitive cognitive behavioural therapy among refugees and in global contexts. Intervention.

[52] Heim E, Kohrt BA (2019). Cultural adaptation of scalable psychological interventions: a new conceptual framework. Clin. Psychol. Eur..

[53] Hwang WC (2009). The formative method for adapting psychotherapy (FMAP): a community-based developmental approach to culturally adapting therapy. Prof. Psychol. Res. Pr..

[54] Barrera M, Castro FG (2006). A heuristic framework for the cultural adaptation of interventions. Clin. Psychol. Sci. Pract..

[55] Benish SG, Quintana S, Wampold BE (2011). Culturally adapted psychotherapy and the legitimacy of myth: a direct-comparison meta-analysis. J. Couns. Psychol..

[56] Hall GCN, Ibaraki AY, Huang ER, Marti CN, Stice E (2016). A meta-analysis of cultural adaptations of psychological interventions. Behav. Ther..

[57] Smith TB, Domenech Rodríguez M, Bernal G (2011). Culture. J. Clin. Psychol..

[58] Soto A, Smith TB, Griner D, Rodríguez MD, Bernal G (2018). Cultural adaptations and therapist multicultural competence: two meta‐analytic reviews. J. Clin. Psychol..

[59] Harper Shehadeh MJ, Heim E, Chowdhary N, Maercker A, Albanese E (2016). Cultural adaptation of minimally guided interventions for common mental disorders: a systematic review and meta-analysis. JMIR Ment. Health.

[60] Sijbrandij M (2017). Expanding the evidence: key priorities for research on mental health interventions for refugees in high-income countries. Epidemiol. Psychiatr. Sci..

[61] Knaevelsrud C, Brand J, Lange A, Ruwaard J, Wagner B (2015). Web-based psychotherapy for posttraumatic stress disorder in war-traumatized Arab patients: randomized controlled trial. J. Med. Internet Res..

[62] Arjadi R (2018). Internet-based behavioural activation with lay counsellor support versus online minimal psychoeducation without support for treatment of depression: a randomised controlled trial in Indonesia. Lancet Psychiatry.

[63] Lindegaard T (2020). Internet-based cognitive behavioural therapy for depression and anxiety among Arabic-speaking individuals in Sweden: a pilot randomized controlled trial. Cogn. Behav. Ther..

[64] Heim, E. et al. Effect of cultural adaptation of a smartphone-based self-help programme on its acceptability and efficacy: study protocol for a randomized controlled trial. *PsychArchives*10.23668/PSYCHARCHIVES.3152 (2020). 10.32872/cpe.2743 PMC11303917 39119053

[65] Fu Z, Burger H, Arjadi R, Bockting CLH (2020). Effectiveness of digital psychological interventions for mental health problems in low-income and middle-income countries: a systematic review and meta-analysis. Lancet Psychiatry.

[66] Ramos, G. & Chavira, D. A. Use of technology to provide mental health care for racial and ethnic minorities: evidence, promise, and challenges. *Cogn. Behav. Pract*. 10.1016/j.cbpra.2019.10.004 (2019).

[67] Escoffery C (2018). A systematic review of adaptations of evidence-based public health interventions globally. Implement. Sci..

[68] Stirman SW, Baumann AA, Miller CJ (2019). The FRAME: an expanded framework for reporting adaptations and modifications to evidence-based interventions. Implement. Sci..

[69] Abi Ramia J (2018). Community cognitive interviewing to inform local adaptations of an e-mental health intervention in Lebanon. Glob. Ment. Health.

[70] Arjadi R, Nauta MH, Suryani AO, Bockting CLH (2018). Guided Act and Feel Indonesia - Internet-based behavioral activation intervention for depression in Indonesia: a systematic cultural adaptation. Makara Hubs-Asia.

[71] Juniar D (2019). Web-based stress management program for university students in Indonesia: systematic cultural adaptation and protocol for a feasibility study. JMIR Res. Protoc..

[72] Lal S (2018). Cultural and contextual adaptation of an ehealth intervention for youth receiving services for first-episode psychosis: adaptation framework and protocol for Horyzons-Canada phase 1. JMIR Res. Protoc..

[73] Salamanca-Sanabria A, Richards D, Timulak L (2019). Adapting an internet-delivered intervention for depression for a Colombian college student population: an illustration of an integrative empirical approach. Internet Inter..

[74] Stoyanov SR (2015). Mobile App Rating Scale: a new tool for assessing the quality of health mobile apps. JMIR mHealth uHealth.

[75] Baumel A, Faber K, Mathur N, Kane JM, Muench F (2017). Enlight: a comprehensive quality and therapeutic potential evaluation tool for mobile and web-based eHealth interventions. J. Med. Internet Res..

[76] Kim P, Eng TR, Deering MJ, Maxfield A (1999). Review of published criteria for evaluating health-related websites. West. J. Med..

[77] Titov N, Schofield C, Staples L, Dear BF, Nielssen O (2018). A comparison of Indigenous and non-Indigenous users of MindSpot: an Australian digital mental health service. Australas. Psychiatry.

[78] Bolinski F (2018). Effectiveness of a transdiagnostic individually tailored Internet-based and mobile-supported intervention for the indicated prevention of depression and anxiety (ICare Prevent) in Dutch college students: study protocol for a randomised controlled trial. Trials.

[79] Rahmadiana M (2019). Guided internet-based transdiagnostic intervention for Indonesian university students with symptoms of anxiety and depression: a pilot study protocol. Internet Inter..

[80] Imamura K (2019). Effects of two types of smartphone-based stress management programmes on depressive and anxiety symptoms among hospital nurses in Vietnam: a protocol for three-arm randomised controlled trial. BMJ Open.

[81] Chen H (2020). Predictors of treatment outcomes and adherence in internet-based cognitive behavioral therapy for social anxiety in China. Behav. Cogn. Psychother.

[82] Kaal E (2020). Testing the efficacy of a minimal-guidance online self-help intervention for alcohol misuse in Estonia: study protocol of a randomized controlled trial. BMC Public Health.

[83] Wasil AR (2020). Harnessing single-session interventions to improve adolescent mental health and well-being in India: development, adaptation, and pilot testing of online single-session interventions in Indian secondary schools. Asian J. Psychiatr..

[84] Okujava N (2019). Digital cognitive behavioral therapy for insomnia – The first Georgian version. Can we use it practice?. Internet Interv..

[85] Yokomitsu K (2020). Gamified mobile computerized cognitive behavioral therapy for Japanese university students with depressive symptoms: protocol for a randomized controlled trial. JMIR Res. Protoc..

[86] Landis JR, Koch GG (1977). The measurement of observer agreement for categorical data. Biometrics.

[87] Brooks LA, Bloomer MJ, Manias E (2019). Culturally sensitive communication at the end-of-life in the intensive care unit: a systematic review. Aust. Crit. Care.

[88] Wang Z, Wang J, Maercker A (2013). Chinese My Trauma Recovery, a web-based intervention for traumatized persons in two parallel samples: randomized controlled trial. J. Med. Internet Res..

[89] Harper Shehadeh MJ (2020). Step-by-Step, an e-mental health intervention for depression: a mixed methods pilot study from Lebanon. Front. Psychiatry.

[90] Shala M (2020). Cultural adaptation of Hap-pas-Hapi, an internet and mobile-based intervention for the treatment of psychological distress among Albanian migrants in Switzerland and Germany. Internet Inter..

[91] Spanhel K (2019). Cultural adaptation of internet interventions for refugees: results from a user experience study in Germany. Internet Inter..

[92] Campbell ANC (2015). Acceptability of a web-based community reinforcement approach for substance use disorders with treatment-seeking American Indians/Alaska Natives. Community Ment. Health J..

[93] Ip P (2016). Effectiveness of a culturally attuned Internet-based depression prevention program for Chinese adolescents: a randomized controlled trial. Depress Anxiety.

[94] Ünlü Ince B (2013). Internet-based, culturally sensitive, problem-solving therapy for Turkish migrants with depression: randomized controlled trial. J. Med. Internet Res..

[95] Wagner B, Schulz W, Knaevelsrud C (2012). Efficacy of an Internet-based intervention for posttraumatic stress disorder in Iraq: a pilot study. Psychiatry Res..

[96] Kanuri N (2020). Examining the initial usability, acceptability and feasibility of a digital mental health intervention for college students in India. Int. J. Psychol..

[97] Kayrouz R (2015). A feasibility open trial of guided Internet-delivered cognitive behavioural therapy for anxiety and depression amongst Arab Australians. Internet Inter..

[98] Kayrouz R, Dear BF, Karin E, Fogliati VJ, Titov N (2016). A pilot study of a clinician-guided internet-delivered cognitive behavioural therapy for anxiety and depression among Arabs in Australia, presented in both English and Arabic languages. Internet Inter..

[99] Kayrouz R (2016). A pilot study of self-guided internet-delivered cognitive behavioural therapy for anxiety and depression among Arabs. Internet Inter..

[100] Paris M (2018). Culturally adapted, web-based cognitive behavioral therapy for Spanish-speaking individuals with substance use disorders: a randomized clinical trial. Am. J. Public Health.

[101] Silva MA (2020). Changes in DSM criteria following a culturally-adapted computerized CBT for Spanish-speaking individuals with substance use disorders. J. Subst. Abus. Treat..

[102] Spitzer RL, Kroenke K, Williams JBW (1999). Validation and utility of a self-report version of PRIME-MD: the PHQ primary care study. JAMA.

[103] Spitzer RL, Kroenke K, Williams JBW, Löwe B (2006). A brief measure for assessing generalized anxiety disorder. Arch. Intern. Med..

[104] Foa EB, Cashman L, Jaycox L, Perry K (1997). The validation of a self-report measure of posttraumatic stress disorder: The Posttraumatic Diagnostic Scale. Psychol. Assess..

[105] Muroff J (2019). An outcome study of the CASA-CHESS smartphone relapse prevention tool for Latinx Spanish-speakers with substance use disorders. Subst. Use Misuse.

[106] Lin LY (2020). An internet-based intervention for individuals with social anxiety and different levels of Taijin Kyofusho in China. J. Cross Cult. Psychol..

[107] Kelders SM, Kok RN, Ossebaard HC, Van Gemert-Pijnen JEWC (2012). Persuasive system design does matter: a systematic review of adherence to web-based interventions. J. Med. Internet Res..

[108] Baumel A, Muench F, Edan S, Kane JM (2019). Objective user engagement with mental health apps: aystematic search and panel-based usage analysis. J. Med. Internet Res..

[109] Van Ballegooijen W (2014). Adherence to internet-based and face-to-face cognitive behavioural therapy for depression: a meta-analysis. PLoS ONE.

[110] Simon N (2019). Acceptability of internet-based cognitive behavioural therapy (i-CBT) for post-traumatic stress disorder (PTSD): a systematic review. Eur. J. Psychotraumatol..

[111] Burchert S (2019). User-centered app adaptation of a low-intensity e-mental health intervention for Syrian refugees. Front. Psychiatry.

[112] Watkins ER, Newbold A (2020). Factorial designs help to understand how psychological therapy works. Front. Psychiatry.

[113] Collins, L. M. *Optimization of Behavioral, Biobehavioral, and Biomedical Interventions: the Multiphase Optimization Strategy (MOST)* (Springer International Publishing, 2018).

[114] von Lersner, U. & Kizilhan, J. I. *Kultursensitive Psychotherapie [Culture-sensitive psychotherapy]*. *Fortschritte der Psychotherapie* - *Band**64* (Hogrefe, 2017).

[115] Liberati A (2009). The PRISMA statement for reporting systematic reviews and meta-analyses of studies that evaluate health care interventions: explanation and elaboration. J. Clin. Epidemiol..

[116] Spanhel K, Balci S, Baumeister H, Bengel J, Sander LB (2020). Cultural adaptation of Internet- and mobile-based interventions for mental disorders: a systematic review protocol. Syst. Rev..

[117] Kampling, H., Baumeister, H., Jackel, W. H. & Mittag, O. Prevention of depression in chronically physically ill adults. *Cochrane Database Syst. Rev*. **3**, CD011246 (2014). 10.1002/14651858.CD011246.pub2 PMC8092431 33667319

[118] Balci S, Spanhel K, Sander LB, Baumeister H (2020). Protocol for a systematic review and meta-analysis of culturally adapted internet- and mobile-based health promotion interventions. BMJ Open.

[119] Covidence. Covidence systematic review software, Veritas Health Innovation, Melbourne, Australia. Available at www.covidence.org.

[120] Sirriyeh R, Lawton R, Gardner P, Armitage G (2012). Reviewing studies with diverse designs: the development and evaluation of a new tool. J. Eval. Clin. Pract..

[121] Cohen J (1960). A coefficient of agreement for nominal scales. Educ. Psychol. Meas..

[122] Hedges, L. & Olkin, I. *Statistical Methods for Meta-analysis* (Academic Press, 1985).

[123] Abuwalla Z (2017). Proposed model for the cultural adaptation of an Internet-based depression prevention intervention (CATCH-IT) for Arab adolescents. Int. J. Adolesc. Med. Health.

[124] Arjadi R, Nauta MH, Bockting CLH (2018). Acceptability of internet-based interventions for depression in Indonesia. Internet Inter..

[125] Choi I (2012). Culturally attuned internet treatment for depression amongst Chinese Australians: a randomised controlled trial. J. Affect. Disord..

[126] Daponte D (2018). Facilitating the dissemination of iCBT for the treatment of anxiety and depression: a feasibility study. Behav. Chang.

[127] Eylem O (2021). Reducing suicidal ideation among Turkish migrants in the Netherlands and in the UK: the feasibility of a randomised controlled trial of a guided online intervention. Pilot Feasibility Stud..

[128] Garabiles MR, Harper Shehadeh M, Hall BJ (2019). Cultural adaptation of a scalable World Health Organization e-mental health program for overseas Filipino workers. JMIR Form. Res..

[129] Gorman JR (2013). Creating a culturally appropriate web-based behavioral intervention for American Indian/ Alaska native women in Southern California: the healthy women healthy native nation study. Am. Indian Alsk. Nativ. Ment. Health Res..

[130] Hiratsuka VY (2019). An internet-based therapeutic tool for American Indian/Alaska native adults with posttraumatic stress disorder: user testing and developmental feasibility study. J. Med. Internet Res..

[131] Lal S (2020). Adaptation of a digital health innovation to prevent relapse and support recovery in youth receiving services for first-episode psychosis: results from the Horyzons-Canada phase 1 study. JMIR Form. Res.

[132] Luo YJ, Jackson T, Stice E, Chen H (2021). Effectiveness of an internet dissonance-based eating disorder prevention intervention among body-dissatisfied young Chinese women. Behav. Ther..

[133] Muroff J (2017). Use of a smartphone recovery tool for Latinos with co-occurring alcohol and other drug disorders and mental disorders. J. Dual Diagn..

[134] Nygren T, Berg M, Sarkohi A, Andersson G (2018). Development of an Internet-based cognitive behavioral therapy self-help program for arabic-speaking immigrants: mixed-methods study. JMIR Res. Protoc..

[135] Nygren T (2019). Internet-based treatment of depressive symptoms in a Kurdish population: a randomized controlled trial. J. Clin. Psychol..

[136] Patel U (2017). Cultural considerations for the adaptation of an Internet-based intervention for depression prevention in Mainland China. Int. J. Adolesc. Med. Health.

[137] Pinto-Bruno ÁC, Pot AM, Kleiboer A, Droes R-M, van Straten A (2019). An online minimally guided intervention to support family and other unpaid carers of people with dementia: protocol for a randomized controlled trial. JMIR Res. Protoc..

[138] Salamanca-Sanabria A (2020). A culturally adapted cognitive behavioral internet-delivered intervention for depressive symptoms: randomized controlled trial. JMIR Ment. Health.

[139] Saulsberry A (2013). Chicago urban resiliency building (CURB): an internet-based depression-prevention intervention for urban African-American and Latino adolescents. J. Child Fam. Stud..

[140] Silva, M. A. et al. Computer-based training for cognitive behavioral therapy for Spanish-speaking substance users: adaptation and satisfaction (unpublished). 10.1080/15332640.2022.2086194 PMC10350903 35714996

[141] Sit HF (2020). The cultural adaptation of Step-by-Step: an intervention to address depression among Chinese young adults. Front. Psychiatry.

[142] Sobowale K (2013). Adaptation of an internet-based depression prevention intervention for Chinese adolescents: from ‘ CATCH-IT’ to ‘grasp the opportunity. Int. J. Adolesc. Med. Health.

[143] Teles S, Napolskij MS, Paúl C, Ferreira A, Seeher K (2020). Training and support for caregivers of people with dementia: The process of culturally adapting the World Health Organization iSupport programme to Portugal. Dementia.

[144] Vöhringer M (2020). Should I stay or must I go? Predictors of dropout in an internet-based psychotherapy programme for posttraumatic stress disorder in Arabic. Eur. J. Psychotraumatol.

[145] Kishimoto T (2016). Internet-based cognitive behavioral therapy for social anxiety with and without guidance compared to a wait list in China: a Propensity Score Study. Psychother. Psychosom..

[146] Berger T, Hohl E, Caspar F (2009). Internet‐based treatment for social phobia: a randomized controlled trial. J. Clin. Psychol..

[147] Perini S, Titov N, Andrews G (2009). Clinician-assisted Internet-based treatment is effective for depression: randomized controlled trial. Aust. N. Z. J. Psychiatry.

[148] Titov N (2011). Transdiagnostic internet treatment for anxiety and depression: a randomised controlled trial. Behav. Res. Ther..

[149] Van Spijker BAJ, Van Straten A, Kerkhof AJFM (2014). Effectiveness of online self-help for suicidal thoughts: results of a randomised controlled trial. PLoS ONE.

[150] Kuhn E (2017). A randomized controlled trial of a smartphone app for posttraumatic stress disorder symptoms. J. Consult. Clin. Psychol..

[151] Voorhees BWVan (2009). Randomized clinical trial of an Internet-based depression prevention program for adolescents (Project CATCH-IT) in primary care: twelve-week outcomes. J. Dev. Behav. Pediatr..

[152] Lange A (2003). Interapy: a controlled randomized trial of the standardized treatment of posttraumatic stress through the internet. J. Consult. Clin. Psychol..

[153] Stice E, Rohde P, Durant S, Shaw H (2012). A preliminary trial of a prototype internet dissonance-based eating disorder prevention program for young women with body image concerns. J. Consult. Clin. Psychol..

[154] McTavish FM, Chih MY, Shah D, Gustafson DH (2008). How patients recovering from alcoholism use a smartphone intervention. J. Dual Diagn..

[155] Andersson G (2005). Internet-based self-help for depression: randomised controlled trial. Br. J. Psychiatry.

[156] Van Straten A (2014). Guided Internet-delivered cognitive behavioural treatment for insomnia: a randomized trial. Psychol. Med..

[157] Kiluk BD (2016). Randomized trial of computerized cognitive behavioral therapy for alcohol use disorders: efficacy as a virtual stand-alone and treatment add-on compared with standard outpatient treatment. Alcohol. Clin. Exp. Res..

[158] Richards D (2015). A randomized controlled trial of an internet-delivered treatment: its potential as a low-intensity community intervention for adults with symptoms of depression. Behav. Res. Ther..

[159] Van Straten A, Cuijpers P, Smits N (2008). Effectiveness of a web-based self-help intervention for symptoms of depression, anxiety, and stress: randomized controlled trial. J. Med. Internet Res..

[160] Steinmetz SE, Benight CC, Bishop SL, James LE (2012). My Disaster Recovery: a pilot randomized controlled trial of an Internet intervention. Anxiety, Stress Coping.

[161] Beck AT, Ward CH, Mendelson M, Mock J, Erbaugh J (1961). An inventory for measuring depression. Arch. Gen. Psychiatry.

[162] Beck AT, Steer RA, Ranieri WF (1988). Scale for suicide ideation: psychometric properties of a self‐report version. J. Clin. Psychol..

[163] Weathers, F. W., Litz, B. T., Herman, D. S., Huska, J. A. & Keane, T. M. The PTSD Checklist (PCL): reliability, validity,and diagnostic utility. Paper presented at the Annual convention of the international society for traumatic stress studies, San Antonio, TX, October (1993).

[164] Radloff LS (1977). The CES-D Scale: A self-report depression scale for research in the general population. Appl. Psychol. Meas..

[165] Horowitz M, Wilner N, Alvarez W (1979). Impact of Event Scale: a measure of subjective stress. Psychosom. Med..

[166] Mattick RP, Clarke JC (1998). Development and validation of measures of social phobia scrutiny fear and social interaction anxiety. Behav. Res. Ther..

[167] Stice E, Fisher M, Martinez E (2004). Eating Disorder Diagnostic Scale: additional evidence of reliability and validity. Psychol. Assess..

[168] Kessler F (2012). Psychometric properties of the sixth version of the Addiction Severity Index (ASI-6) in Brazil. Rev. Bras. Psiquiatr..

[169] Bastien CH, Valliéres A, Morin CM (2001). Validation of the Insomnia Severity Index as an outcome measure for insomnia research. Sleep. Med..

[170] Buysse DJ, Reynolds CF, Monk TH, Berman SR, Kupfer DJ (1989). The Pittsburgh Sleep Quality Index: a new instrument for psychiatric practice and research. Psychiatry Res..

[171] Kessler RC (2002). Short screening scales to monitor population prevalences and trends in non-specific psychological distress. Psychol. Med..

