# Supplementary material for: Cultural adaptation of internet- and mobile-based interventions for mental disorders: a systematic review
Source: NPJ Digit Med. 2021 Aug 25;4:128. doi: 10.1038/s41746-021-00498-1 (PMC8387403; doi:10.1038/s41746-021-00498-1)
Supplement: Supplementary file 1 — Supplementary information [file 41746_2021_498_MOESM1_ESM.pdf]

Supplementary information

This supplementary information has been provided by the authors to give readers additional information about their work.

Supplement to:

Cultural adaptation of internet- and mobile-based interventions for mental disorders: a systematic review

Kerstin Spanhel [1]*, Sumeyye Balci [2], Felicitas Feldhahn [1], Juergen Bengel [1], Harald Baumeister [2], & Lasse B. Sander [1]

1 Department of Rehabilitation Psychology and Psychotherapy, Institute of Psychology, University of Freiburg, Engelbergerstr. 41, D-79085 Freiburg, Germany

2 Department of Clinical Psychology and Psychotherapy, Institute of Psychology and Education, Ulm University, Lise-Meitner-Str. 16, D-89081 Ulm, Germany

* Corresponding author: kerstin.spanhel@psychologie.uni-freiburg.de

Table of Content:

Supplementary Tables *3*

Supplementary Table 1: PRISMA checklist **3**

Supplementary Table 2: PsycINFO search term **6**

Supplementary Table 3: Details on the original and culturally adapted internet- and mobile-based interventions (IMI) used in the included articles **24**

Supplementary Table 4: Quality ratings of the included studies using the Quality Assessment Tool for Reviewing Studies with Diverse Designs [85] **33**

Supplementary Table 5: Detailed illustration of the conducted cultural adaptations of the 17 extracted aspects **39**

Supplementary Table 6: Results of the post hoc conducted analyses **109**

Supplementary References *111*

Supplementary Tables

Supplementary Table 1: PRISMA checklist

Section/topic	#	Checklist item	Reported on page #
**TITLE**			
Title	1	Identify the report as a systematic review, meta-analysis, or both.	1
**ABSTRACT**			
Structured summary	2	Provide a structured summary including, as applicable: background; objectives; data sources; study eligibility criteria, participants, and interventions; study appraisal and synthesis methods; results; limitations; conclusions and implications of key findings; systematic review registration number.	2
**INTRODUCTION**			
Rationale	3	Describe the rationale for the review in the context of what is already known.	3-5
Objectives	4	Provide an explicit statement of questions being addressed with reference to participants, interventions, comparisons, outcomes, and study design (PICOS).	6
**METHODS**			
Protocol and registration	5	Indicate if a review protocol exists, if and where it can be accessed (e.g., Web address), and, if available, provide registration information including registration number.	6
Eligibility criteria	6	Specify study characteristics (e.g., PICOS, length of follow-up) and report characteristics (e.g., years considered, language, publication status) used as criteria for eligibility, giving rationale.	6-7
Information sources	7	Describe all information sources (e.g., databases with dates of coverage, contact with study authors to identify additional studies) in the search and date last searched.	7
Search	8	Present full electronic search strategy for at least one database, including any limits used, such that it could be repeated.	7, Suppl Table 2
Study selection	9	State the process for selecting studies (i.e., screening, eligibility, included in systematic review, and, if applicable, included in the meta-analysis).	7
Data collection process	10	Describe method of data extraction from reports (e.g., piloted forms, independently, in duplicate) and any processes for obtaining and confirming data from investigators.	8
Data items	11	List and define all variables for which data were sought (e.g., PICOS, funding sources) and any assumptions and simplifications made.	8

Section/topic	#	Checklist item	Reported on page #
Risk of bias in individual studies	12	Describe methods used for assessing risk of bias of individual studies (including specification of whether this was done at the study or outcome level), and how this information is to be used in any data synthesis.	8
Summary measures	13	State the principal summary measures (e.g., risk ratio, difference in means).	9
Synthesis of results	14	Describe the methods of handling data and combining results of studies, if done, including measures of consistency (e.g., I^2) for each meta-analysis.	9
Risk of bias across studies	15	Specify any assessment of risk of bias that may affect the cumulative evidence (e.g., publication bias, selective reporting within studies).	N/A
Additional analyses	16	Describe methods of additional analyses (e.g., sensitivity or subgroup analyses, meta-regression), if done, indicating which were pre-specified.	9-10
**RESULTS**			
Study selection	17	Give numbers of studies screened, assessed for eligibility, and included in the review, with reasons for exclusions at each stage, ideally with a flow diagram.	10, Figure 1
Study characteristics	18	For each study, present characteristics for which data were extracted (e.g., study size, PICOS, follow-up period) and provide the citations.	10-11, Table 2
Risk of bias within studies	19	Present data on risk of bias of each study and, if available, any outcome level assessment (see item 12).	11, Suppl Table 4
Results of individual studies	20	For all outcomes considered (benefits or harms), present, for each study: (a) simple summary data for each intervention group (b) effect estimates and confidence intervals, ideally with a forest plot.	Table 2, 3, 4
Synthesis of results	21	Present results of each meta-analysis done, including confidence intervals and measures of consistency.	12-15
Risk of bias across studies	22	Present results of any assessment of risk of bias across studies (see Item 15).	N/A
Additional analysis	23	Give results of additional analyses, if done (e.g., sensitivity or subgroup analyses, meta-regression [see Item 16]).	15
**DISCUSSION**			

Section/topic	#	Checklist item	Reported on page #
Summary of evidence	24	Summarize the main findings including the strength of evidence for each main outcome; consider their relevance to key groups (e.g., healthcare providers, users, and policy makers).	15-16
Limitations	25	Discuss limitations at study and outcome level (e.g., risk of bias), and at review-level (e.g., incomplete retrieval of identified research, reporting bias).	18-19
Conclusions	26	Provide a general interpretation of the results in the context of other evidence, and implications for future research.	16-17, 19
**FUNDING**			
Funding	27	Describe sources of funding for the systematic review and other support (e.g., supply of data); role of funders for the systematic review.	19-20

From: Moher D, Liberati A, Tetzlaff J, Altman DG, The PRISMA Group (2009). Preferred Reporting Items for Systematic Reviews and Meta-Analyses: The PRISMA Statement. PLoS Med 6(7): e1000097. doi:10.1371/journal.pmed1000097

For more information, visit: **www.prisma-statement.org**.

Supplementary Table 2: PsycINFO search term

**S1**	DE „internet“
**S2**	DE „Mobile Devices“
**S3**	DE „Cellular Phones“
**S4**	DE „Computers“
**S5**	TI computer
**S6**	TI website
**S7**	TI websites
**S8**	TI web
**S9**	TI online
**S10**	TI internet
**S11**	TI webbased
**S12**	TI internetbased
**S13**	TI media-based
**S14**	TI computerized
**S15**	TI computerised
**S16**	TI cyber
**S17**	TI mobile
**S18**	TI App
**S19**	TI Apps
**S20**	TI smartphone
**S21**	TI smartphones
**S22**	TI phone
**S23**	TI phones
**S24**	S1 OR S2 OR S3 OR S4 OR S5 OR S6 OR S7 OR S8 OR S9 OR S10 OR S11 OR S12 OR S13 OR S14 OR S15 OR S16 OR S17 OR S18 OR S19 OR S20 OR S21 OR S22 OR S23
**S25**	DE „Psychotherapy”
**S26**	DE „Analytical Psychotherapy”
**S27**	DE „Behavior Therapy“
**S28**	DE „Brief Psychotherapy“
**S29**	DE „Cognitive Behavior Therapy“
**S30**	DE „Narrative Therapy“
**S31**	DE „Psychodynamic Psychotherapy“
**S32**	DE „Psychodynamic Psychotherapy“
**S33**	DE „Psychotherpeutic Counseling“
**S34**	DE „Behavior Modification“
**S35**	DE „Self-Management”
**S36**	DE „Self-Instructional Training“
**S37**	DE „Self-Help Techniques“

**S38**	DE „Psychotherapeutic Techniques“
**S39**	DE „Cognitive Techniques“
**S40**	DE „Cognitive Restructuring“
**S41**	DE „Cognitive Therapy“
**S42**	DE „Therapeutic Processes“
**S43**	DE „intervention“
**S44**	TI Intervention OR AB Intervention
**S45**	TI Interventions OR AB Interventions
**S46**	TI treatment OR AB treatment
**S47**	TI treatments OR AB treatments
**S48**	TI therapy OR AB therapy
**S49**	TI therapies OR AB therapies
**S50**	TI therapeutics OR AB therapeutics
**S51**	TI therapeutic OR AB therapeutic
**S52**	TI psychotherapy OR AB psychotherapy
**S53**	TI psychotherapies OR AB psychotherapies
**S54**	TI psychoeducation OR AB psychoeducation
**S55**	TI psychotherapeutic OR AB psychotherapeutic
**S56**	TI psychotherapeutics OR AB psychotherapeutics
**S57**	TI cbt OR AB cbt
**S58**	TI program OR AB program
**S59**	TI programs OR AB programs
**S60**	TI programme OR AB programme
**S61**	TI programmes OR AB programmes
**S62**	TI training OR AB training
**S63**	TI trainings OR AB trainings
**S64**	TI lesson OR AB lesson
**S65**	TI lessons OR AB lessons
**S66**	TI counselling OR AB counselling
**S67**	TI counseling OR AB counseling
**S68**	TI coaching OR AB coaching
**S69**	TI self-help OR AB self-help
**S70**	TI self-care OR AB self-care
**S71**	TI selfcare OR AB selfcare
**S72**	TI self-change OR AB self-change
**S73**	TI self-instruction OR AB self-instruction
**S74**	DE „Computer Assisted Therapy”
**S75**	DE „Online Therapy”
**S76**	DE „Telemedicine”
**S77**	TI telemedicine OR AB telemedicine

**S78**	TI telehealth OR AB telehealth
**S79**	TI tele-health OR AB tele-health
**S80**	TI telecare OR AB telecare
**S81**	TI eHealth OR AB eHealth
**S82**	TI e-health OR AB e-health
**S83**	TI „electronic health" OR AB „electronic health"
**S84**	TI „e-mental health" OR AB „e-mental health"
**S85**	TI e-therapy OR AB e-therapy
**S86**	TI e-therapeutic OR AB e-therapeutic
**S87**	TI mHealth OR AB mHealth
**S88**	TI m-health OR AB m-health
**S89**	TI „mobile health" OR AB „mobile health"
**S90**	TI cCBT OR AB cCBT
**S91**	TI iCBT OR AB iCBT
**S92**	S74 OR S75 OR S76 OR S77 OR S78 OR S79 OR S80 OR S81 OR S82 OR S83 OR S84 OR S85 OR S86 OR S87 OR S88 OR S89 OR S90 OR S91
**S93**	S25 OR S26 OR S27 OR S28 OR S29 OR S30 OR S31 OR S32 OR S33 OR S34 OR S35 OR S36 OR S37 OR S38 OR S39 OR S40 OR S41 OR S42 OR S43 OR S44 OR S45 OR S46 OR S47 OR S48 OR S49 OR S50 OR S51 OR S52 OR S53 OR S54 OR S55 OR S56 OR S57 OR S58 OR S59 OR S60 OR S61 OR S62 OR S63 OR S64 OR S65 OR S66 OR S67 OR S68 OR S69 OR S70 OR S71 OR S72 OR S73
**S94**	S24 AND S93
**S95**	S92 OR S94
**S96**	DE "Cross Cultural Treatment"
**S97**	DE "Cross Cultural Counseling"
**S98**	DE "Multicultural Counseling"
**S99**	DE "Cross Cultural Psychology"
**S100**	DE "International Psychology"
**S101**	DE "Transcultural Psychiatry"
**S102**	DE "Cross Cultural Differences"
**S103**	DE "Racial and Ethnic Differences"
**S104**	DE "Cultural Sensitivity"
**S105**	DE "Multiculturalism"
**S106**	DE "Sociocultural Factors"
**S107**	TX "Cultural adaptation" OR TX "Cultural adaptations" OR TX "Culturally adapted"
**S108**	TX "cultural attunement" OR TX "cultural attunements" OR TX "culturally attuned"
**S109**	TX "cultural adjustment" OR TX "cultural adjustments" OR TX "culturally adjusted
**S110**	TX "culturally tailored" OR TX "cultural tailoring"
**S111**	TX "culturally tailored" OR TX "cultural tailoring"
**S112**	TX "Cultural modification" OR TX "Cultural modifications" OR TX "Culturally modified"
**S113**	TX "contextual adaptation" OR TX "contextual adaptation"

S114 TX “local adaptation” OR TX “local adaptations”
S115 TX "Cultural consideration" OR TX "Cultural considerations"
S116 TX "culturally suitable" OR TX "cultural suitability"
S117 TX "culturally adequate" OR TX "cultural adequacy"
S118 TX "culturally appropriate" OR TX "cultural appropriateness"
S119 TX "Cultural influence" OR TX "Cultural influences" OR TX "Culturally influenced"
S120 TI CALD OR AB CALD
S121 TI "culturally diverse" OR AB "culturally diverse" OR TI "cultural diversity" OR AB "cultural diversity"
S122 TI "linguistically diverse" OR AB "linguistically diverse" OR TI "linguistic diversity" OR AB "linguistic diversity"
S123 TI "Cultural difference" OR AB "cultural difference" OR TI "Cultural differences" OR AB "Cultural differences"
S124 TI "Culturally specific" OR AB "Culturally specific" OR TI "Cultural specificity" OR AB "Cultural specificity" OR TI "Cultural specificities" OR AB "Cultural specificities" OR TI "Cultural specific" OR AB "Cultural specific"
S125 TI "Cultural identities" OR AB "Cultural identities" OR TI "Cultural identity" OR AB "Cultural identity"
S126 TI "cultural background" OR AB "cultural background" OR TI "cultural backgrounds" OR AB "cultural backgrounds"
S127 TI “culturally compared" OR AB “culturally compared" OR TI “cultural comparison” OR AB “cultural comparison” OR TI “cultural comparisons” OR AB “cultural comparisons”
S128 TI “cultural group” OR AB “cultural group” OR TI “cultural groups” OR AB “cultural groups”
S129 TI “cultural aspect” OR AB “cultural aspect” OR TI “cultural aspects” OR AB “cultural aspects”
S130 TI "Cultural factor" OR AB "Cultural factor" OR TI "Cultural factors" OR AB "Cultural factors"
S131 TI "culturally enhanced" OR AB "culturally enhanced"
S132 TI "culturally grounded" OR AB "culturally grounded"
S133 TI "Cultural equivalence" OR AB "Cultural equivalence"
S134 TI "cultural fit" OR AB "cultural fit"
S135 TI "cultural sensitivity" OR AB "cultural sensitivity" OR TI "cultural sensitive" OR AB "cultural sensitive" OR TI "culturally sensitive" OR AB "culturally sensitive"
S136 TI "cultural awareness" OR AB "cultural awareness"
S137 TI "Cultural knowledge" OR AB "Cultural knowledge"
S138 TI “cultural understanding” OR AB “cultural understanding”
S139 TI “cultural expertise” OR AB “cultural expertise”
S140 TI “cultural skills” OR AB “cultural skills”
S141 TI "Culturally informed" OR AB "Culturally informed"
S142 TI "Culturally safe" OR AB "Culturally safe" OR TI "Cultural safety" OR AB "Cultural safety
S143 TI "Culturally responsive" OR AB "Culturally responsive" OR TI "cultural responsiveness" OR AB "cultural responsiveness"
S144 TI "Cultural focus" OR AB "Cultural focus" OR TI "Culturally focused" OR AB "Culturally focused"
S145 TI "Culturally relevant" OR AB "Culturally relevant" OR TI "Cultural relevance" OR AB "Cultural relevance"
S146 TI "Culturally congruent" OR AB "Culturally congruent" OR TI "Cultural congruence" OR AB "Cultural congruence"
S147 TI "Culturally consistent" OR AB "Culturally consistent" OR TI "cultural consistency" OR AB "cultural consistency"
S148 TI “multi cultur*” OR AB “multi cultur*” OR TI multicultur* OR AB multicultur*
S149 TI “inter cultur*” OR AB “inter cultur*” OR TI “intercultur*” OR AB “intercultur
S150 TI “cross cultur*” OR AB “cross cultur*” OR TI “crosscultur*” OR AB “crosscultur*”

S151 TI sociocultur* OR AB sociocultur* OR TI "socio cultur*" OR AB "socio cultur*"
S152 TI bicultur* OR AB bicultur*
S153 TI bicultur* OR bicultur*
S154 TI "ethnic bias" OR AB "ethnic bias" OR TI "ethnic biases" OR AB "ethnic biases"
S155 TI "Racial bias" OR AB "Racial bias" OR TI "Racial biases" OR AB "Racial biases"
S156 TI "ethnic disparity" OR AB "ethnic disparity" OR TI "ethnic disparities" OR AB "ethnic disparities"
S157 TI "Racial disparity" OR AB "Racial disparity" OR TI "Racial disparities" OR AB "Racial disparities"
S158 TI "ethnic diversity" OR AB "ethnic diversity" OR TI "ethnic diversities" OR AB "ethnic diversities" OR TI "ethnically diverse" OR AB "ethnically diverse"
S159 TI “racial group” OR AB “racial group” OR TI "racial groups" OR AB "racial groups"
S160 TI “ethnic group” OR AB “ethnic group” OR TI "ethnic groups" OR AB "ethnic groups"
S161 TI “ethnic aspect” OR AB “ethnic aspect” OR TI "ethnic aspects" OR AB "ethnic aspects"
S162 TI “racial aspect” OR AB “racial aspect” OR TI "racial aspects" OR AB "racial aspects"
S163 TI racial OR TI race OR TI races OR TI ethnic OR TI ethnicity OR TI ethnicities OR TI culture OR TI cultures OR TI cultural OR TI minority OR TI minorities OR TI language OR TI linguistic
S164 TI barriers N2 S163
S165 AB racial OR AB race OR AB races OR AB ethnic OR AB ethnicity OR AB ethnicities OR AB culture OR AB cultures OR AB cultural OR AB minority OR AB minorities OR AB language OR AB linguistic
S166 AB barriers N2 S165
S167 TI racial OR TI race OR TI races OR TI ethnic OR TI ethnicity OR TI ethnicities OR TI culture OR TI cultures OR TI cultural OR TI minority OR TI minorities
S168 AB racial OR AB race OR AB races OR AB ethnic OR AB ethnicity OR AB ethnicities OR AB culture OR AB cultures OR AB cultural OR AB minority OR AB minorities
S169 DE "Communication Barriers" AND (S167 OR S168)
S170 DE "Health disparities" AND (S167 OR S168)
S171 (TI "Health inequality" OR AB "Health inequality") AND (S167 OR S168)
S172 DE “Treatment compliance” AND (S167 OR S168)
S173 TI “culturally acceptable” OR AB “culturally acceptable” OR TI “cultural acceptability” OR AB “cultural acceptability”
S174 TI "Cultural socialisation" OR AB "Cultural socialisation" OR TI "Cultural socialization" OR AB "Cultural socialization"
S175 S96 OR S97 OR S98 OR S99 OR S100 OR S101 OR S102 OR S103 OR S104 OR S105 OR S106 OR S107 OR S108 OR S109 OR S110 OR S111 OR S112 OR S113 OR S114 OR S115 OR S116 OR S117 OR S118 OR S119 S120 OR S121 OR S122 OR S123
S176 S124 OR S125 OR S126 OR S127 OR S128 OR S129 OR S130 OR S131 OR S132 OR S133 OR S134 OR S135 OR S136 OR S137 OR S138 OR S139 OR S140 OR S141 OR S142 OR S143 OR S144 OR S145 OR S146 OR S147 OR S148 OR S149 OR S150 OR S151 OR S152 OR S153
S177 S154 OR S155 OR S156 OR S157 OR S158 OR S159 OR S160 OR S161 OR S162 OR S164 OR S166 OR S169 OR S170 OR S171 OR S172 OR S173 OR S174
S178 S175 OR S176 OR S177
S179 TI Africa OR TI African OR TI Africans
S180 TI Asia OR TI Asian OR TI Asians
S181 TI “Middle America” OR TI “Middle American” OR TI “Middle Americans”

S182 TI “South America” OR TI “South American” OR TI “South Americans”
S183 TI “Central America” OR TI “Central American” OR TI “Central Americans”
S184 TI Arab OR TI Arabs
S185 TI “Middle East” OR TI “Middle Eastern”
S186 TI Afghanistan OR TI Afghan OR TI Afghans
S187 TI Albania OR TI Albanian OR TI Albanians
S188 TI Algeria OR TI Algerian OR TI Algerians
S189 TI Angolans OR TI Angolan OR TI Angolans
S190 TI Antilles
S191 TI Antigua OR TI Antiguan OR TI Antiguans OR TI Barbuda OR TI Barbadian OR TI Barbadians
S192 TI Argentina OR TI Argentinian OR TI Argentine OR TI Argentines OR TI Argentinean OR TI Argentinians OR TI Argentineans
S193 TI Armenia OR TI Armenian OR TI Armenians
S194 TI Aruba OR TI Aruban OR TI Arubans
S195 TI Azerbaijan OR TI Azerbaijani OR TI Azerbaijanis OR TI Azeri
S196 TI Bahamas
S197 TI Bahrain OR TI Bahraini OR TI Bahrainis
S198 TI Bajun OR TI Bajuni OR TI Bajunis
S199 TI Bangladesh OR TI Bangladeshi OR TI Bangladeshis
S200 TI Barbados
S201 TI Belize OR TI Belizean OR TI Belizeans
S202 TI Benin OR TI Beninese
S203 TI Bermuda
S204 TI Byelarus OR TI Byelorussian OR TI Belarus OR TI Belorussian OR TI Belorussia OR TI Belarussian OR TI Byelorussions OR TI Belorussians OR TI Belarussions
S205 TI Bhutan OR TI Bhutanese
S206 TI Bolivia OR TI Bolivian OR TI Bolivians
S207 TI Bosnia OR TI Bosnian OR TI Bosnians
S208 TI Herzegovina OR TI Hercegovina OR TI Herzegovinian OR TI Herzegovinians
S209 TI Botswana OR TI Batswana
S210 TI Brasil OR TI Brazil OR TI Brazilian OR TI Brazils
S211 TI Brunei OR TI Darussalam
S212 TI Bulgaria OR TI Bulgarian OR TI Bulgarians
S213 TI Burkina OR TI Burkinabe OR TI Faso OR TI Fasso OR TI Volta
S214 TI Burundi OR TI Burundian OR TI Burundians OR TI Urund
S215 TI Caledonia OR TI Caledonian OR TI Caledonians
S216 TI Cambodia OR TI Cambodian OR TI Cambodians OR TI Khmer OR TI Kampuchea
S217 TI Cameroon OR TI Cameroons OR TI Cameroonian OR TI Cameron OR TI Camerons
S218 TI "Cape Verde" OR TI "Cape Verdian" OR TI "Cabo Verde" OR TI "Cabo Verdian" OR TI "Cabo Verdians" OR TI "Cape Verdians" OR TI "Cape Verdeans" OR TI "Cape Verdean"
S219 TI Caribbean OR TI Caribbeans

**S220**	TI Chad OR TI Chadian OR TI Chadians
**S221**	TI Chile OR TI Chilean OR TI Chileans
**S222**	TI China OR TI Chinese
**S223**	TI Colombia OR TI Colombian OR TI Colombians
**S224**	TI Comoros OR TI Comoro OR TI Comores OR TI Comoran OR TI Comorans
**S225**	TI Mayotte OR TI Mahorais
**S226**	TI Congo OR TI Congolese OR TI Zaire
**S227**	TI "Cook Islands"
**S228**	TI Rica OR TI Rican OR TI Ricans
**S229**	TI d'Ivoire OR TI Ivory OR TI Ivoirian OR TI Ivoirians
**S230**	TI Cuba OR TI Cuban OR TI Cubans
**S231**	TI Curacao OR TI Curacaoan OR TI Curacaoans
**S232**	TI Cyprus OR TI Cyprian OR TI Cyprians
**S233**	TI Djibouti
**S234**	TI Dominica OR TI Dominican OR TI Dominicans
**S235**	TI Ecuador OR TI Ecuadorian OR TI Ecuadorians
**S236**	TI Egypt OR TI Egyptian OR TI Egyptians
**S237**	TI Salvador OR TI Salvadorian OR TI Salvadorians
**S238**	TI Emirates OR TI Emirati
**S239**	TI Eritrea OR TI Eritrean OR TI Eritreans
**S240**	TI Eswatini OR TI Swaziland OR TI Swazi OR TI Swazis
**S241**	TI Ethiopia OR TI Ethiopian OR TI Ethiopians
**S242**	TI Faroe
**S243**	TI Fiji OR TI Fijian
**S244**	TI Gabon OR TI Gabonese
**S245**	TI Gambia OR TI Gambian OR TI Gambians
**S246**	TI Georgia OR TI Georgian OR TI Georgians
**S247**	TI Ghana OR TI Ghanaian OR TI Ghanaians
**S248**	TI Gibraltar OR TI Gibraltarian OR TI Gibraltarians
**S249**	TI Grenada OR TI Grenadian OR TI Grenadians
**S250**	TI Guam OR TI Guamanian OR TI Guamanians OR TI Chamorro
**S251**	TI Guatemala OR TI Guatemalan OR TI Guatemalans
**S252**	TI Guinea OR TI Guinean OR TI Guineans
**S253**	TI Guiana OR TI Guyana OR TI Guyanese OR TI Guianan OR TI Guianans OR TI Guianese
**S254**	TI Haiti OR TI Haitian OR TI Haitians
**S255**	TI Honduras OR TI Honduran OR TI Hondurans
**S256**	TI "Hong Kong" OR TI "Hong Kongese"
**S257**	TI India OR TI Indian OR TI Indians
**S258**	TI Indonesia OR TI Indonesian OR TI Indonesians
**S259**	TI Iran OR TI Iranian OR TI Iranians

**S260**	TI Persia OR TI Persian OR TI Persians
**S261**	TI Iraq OR TI Iraqi
**S262**	TI Israel OR TI Israeli
**S263**	TI Jamaica OR TI Jamaican OR TI Jamaicans
**S264**	TI Japan OR TI Japanese
**S265**	TI Jordan OR TI Jordanian OR TI Jordanians
**S266**	TI Kazakhstan OR TI Kazakh OR TI Kazakhs
**S267**	TI Kenya OR TI Kenyan OR TI Kenyans
**S268**	TI Kiribati OR TI Kiribatian OR TI Kiribatians
**S269**	TI Korea OR TI Korean OR TI Koreans
**S270**	TI Kosovo OR TI Kosovar
**S271**	TI Kuwait OR TI Kuwaiti OR TI Kuwaitis
**S272**	TI Kyrgyzstan OR TI Kirghizia OR TI Kyrgyz OR TI Kirghiz OR TI Kirgizstan OR TI Kyrgyzstani OR TI Kyrgyzstanis
**S273**	TI Lao OR TI Laos OR TI Laotian OR TI Laotians
**S274**	TI Latin OR TI Latino OR TI Latinos
**S275**	TI Lebanon OR TI Lebanese
**S276**	TI Lesotho OR TI Lesothan OR TI Lesothans OR TI Basutoland OR TI Basotho
**S277**	TI Liberia OR TI Liberian OR TI Liberians
**S278**	TI Libya OR TI Libyan OR TI Libyans
**S279**	TI Macao OR TI Macau OR TI Macanese
**S280**	TI Macedonia OR TI Macedonian OR TI Macedonians
**S281**	TI Madagasca OR TI Madagascan OR TI Madagascans OR TI Malagasy
**S282**	TI Malawi OR TI Malawian OR TI Nyasaland
**S283**	TI Malaysia OR TI Malaysian OR TI Malaysians OR TI Malaya OR TI Malay OR TI Sabah OR TI Sarawak
**S284**	TI Maldives OR TI Maldivian OR TI Maldivians
**S285**	TI Mali OR TI Malian OR TI Malians
**S286**	TI Mariana
**S287**	TI Marshall OR TI Marshallese
**S288**	TI Mauritania OR TI Mauritanian OR TI Mauritanians
**S289**	TI Mauritius OR TI Mauritian OR TI Mauritians OR TI Agalega
**S290**	TI Mexico OR TI Mexican OR TI Mexicans
**S291**	TI Micronesia OR TI Micronesian OR TI Micronesians OR TI Mariana
**S292**	TI moldova OR TI Moldovian OR TI Moldovia OR TI Moldovan OR TI Moldovans OR TI Moldovians
**S293**	TI Monaco OR TI Monacan OR TI Monacans
**S294**	TI Mongolia OR TI Mongolian OR TI Mongolians
**S295**	TI Montenegro OR TI Montenegrin OR TI Montenegrins
**S296**	TI Montserrat
**S297**	TI Morocco OR TI Moroccan OR TI Moroccans OR TI Ifni
**S298**	TI Mozambique OR TI Mozambican OR TI Mozambicans
**S299**	TI myanmar OR TI Myanmarese OR TI Burma OR TI Burmese

**S300**	TI Namibia OR TI Namibian OR TI Namibians
**S301**	TI Nauru OR TI Nauruan OR TI Nauruans
**S302**	TI Nepal OR TI Nepalese
**S303**	TI Nicaragua OR TI Nicaraguan OR TI Nicaraguans
**S304**	TI Niger
**S305**	TI Nigeria OR TI Nigerian OR TI Nigerians
**S306**	TI Niue
**S307**	TI Oman OR TI Omani OR TI Omanis OR TI Muscat
**S308**	TI Pakistan OR TI Pakistani OR TI Pakistanis
**S309**	TI Palau OR TI Palauan OR TI Palauans
**S310**	TI Palestina OR TI Palestinian OR TI Palestinians OR TI Gaza OR TI West-Bank OR TI "West Bank"
**S311**	TI Panama OR TI Panamanian OR TI Panamanians
**S312**	TI Paraguay OR TI Paraguayan OR TI Paraguayans
**S313**	TI Peru OR TI Peruvian OR TI Peruvians
**S314**	TI Philippines OR TI Philipines OR TI Phillipines OR TI Phillippines OR TI Filipino OR TI Filipinos OR TI Philippino OR TI Philippinos
**S315**	TI Polynesia OR TI Polynesian OR TI Polynesians
**S316**	TI "Puerto Rico" OR TI "Puerto Rican" OR TI "Puerto Ricans"
**S317**	TI Qatar OR TI Qatari OR TI Qataris
**S318**	TI Rhodesia OR TI Rhodesian OR TI Rhodesians
**S319**	TI Romania OR TI Roumania OR TI Romanian OR TI Romanians OR TI Rumania
**S320**	TI Russia OR TI Russian OR TI Russians
**S321**	TI Rwanda OR TI Ruanda OR TI Rwandan OR TI Rwandans
**S322**	TI Kitts OR TI Nevis
**S323**	TI Lucia OR TI Lucian OR TI Lucians
**S324**	TI Vincent OR TI Grenadines
**S325**	TI "Saint Helena"
**S326**	TI Samoa OR TI Samoan OR TI Samoans
**S327**	TI "San Marino" OR TI "San Marinese"
**S328**	TI "Sao Tome" OR TI "Sao Tomean" OR TI "Sao Tomeans" OR TI Principe
**S329**	TI Saudi OR TI Arabian OR TI Arabians OR TI Arabia
**S330**	TI Senegal OR TI Senegalese
**S331**	TI Serbia OR TI Serbian OR TI Serbians
**S332**	TI seychelles OR TI Seychellois
**S333**	TI Sierra OR TI Leone OR TI Leonean OR TI Leoneans
**S334**	TI Singapore OR TI Singaporean OR TI Singaporeans
**S335**	TI Slovakia OR TI Slovakian OR TI Slovakians
**S336**	TI Slovenia OR TI Slovenian OR TI Slovenians
**S337**	TI Solomon
**S338**	TI Somalia OR TI Somali OR TI Somalis OR TI Somaliland OR TI Somalian OR TI Somalians
**S339**	TI "Sri Lanka" OR TI "Sri Lankan" OR TI "Sri Lankans" OR TI Ceylon

**S340**	TI Sudan OR TI Sudanese
**S341**	TI Suriname OR TI Surinam OR TI Surinen
**S342**	TI Syria OR TI Syrian OR TI Syrians
**S343**	TI Taiwan OR TI Taiwanese
**S344**	TI Tajikistan OR TI Tadzhikistan OR TI Tadjikistan OR TI Tadzhik OR TI Tadzhiks OR TI Tajik OR TI Tajiks
**S345**	TI Tanzania OR TI Tanzanian OR TI Tanzanians
**S346**	TI Thailand OR TI Thai OR TI Thais
**S347**	TI Timor OR TI Timur OR TI Atoni OR TI Atonis OR TI Leste
**S348**	TI Tobagonian OR TI Tobagonians
**S349**	TI Togo OR TI Togolese
**S350**	TI Tokelau
**S351**	TI Tonga OR TI Tongan OR TI Tongans
**S352**	TI Tobago OR TI Trinidad OR TI Trinidadian OR TI Trinidadians
**S353**	TI Tunisia OR TI Tunisian OR TI Tunisians
**S354**	TI Turkey OR TI Turkish
**S355**	TI Turkmenistan OR TI Turkmen OR TI Turkmenistani OR TI Turkmenistanis
**S356**	TI Tuvalu OR TI Tuvaluan OR TI Tuvaluans
**S357**	TI Uganda OR TI Ugandan OR TI Ugandans
**S358**	TI Ukraine OR TI Ukrainian OR TI Ukrainians
**S359**	TI Uruguay OR TI Uruguayan OR TI Uruguayans
**S360**	TI USSR OR TI Soviet OR TI Soviets
**S361**	TI Uzbekistan OR TI Uzbek OR TI Uzbeks
**S362**	TI Vanuatu OR TI Vanuatuan OR TI Vanuatuans
**S363**	TI Venezuela OR TI Venezuelan OR TI Venezuelans
**S364**	TI Vietnam OR TI "Viet Nam" OR TI Vietnamese
**S365**	TI Wallis
**S366**	TI Yemen OR TI Yemeni OR TI Yemenis
**S367**	TI Yugoslavia OR TI Yugoslavian OR TI Yugoslavians OR TI Yugoslav OR TI Yugoslavs OR TI Jugoslavia
**S368**	TI Zambia OR TI Zambian OR TI Zambians
**S369**	TI Zimbabwe OR TI Zimbabwean OR TI Zimbabweans
**S370**	S179 OR S180 OR S181 OR S182 OR S183 OR S184 OR S185 OR S186 OR S187 OR S188 OR S189 OR S190 OR S191 OR S192 OR S193 OR S194 OR S195 OR S196 OR S197 OR S198 OR S199 OR S200 OR S201 OR S202 OR S203 OR S204 OR S205 OR S206 OR S207 OR S208 OR S209 OR S210 OR S211 OR S212 OR S213 OR S214 OR S215 OR S216 OR S217 OR S218 OR S219 OR S220 OR S221 OR S222 OR S223 OR S224 OR S225 OR S226 OR S227 OR S228 OR S229 OR S230 OR S231 OR S232 OR S233 OR S234 OR S235 OR S236 OR S237 OR S238 OR S239 OR S240 OR S241 OR S242 OR S243 OR S244 OR S245 OR S246 OR S247 OR S248 OR S249 OR S250 OR S251 OR S252 OR S253 OR S254 OR S255 OR S256 OR S257 OR S258 OR S259 OR S260 OR S261 OR S262 OR S263 OR S264 OR S265 OR S266 OR S267 OR S268 OR S269 OR S270 OR S271 OR S272 OR S273 OR S274 OR S275 OR S276 OR S277 OR S278 OR S279 OR S280 OR S281 OR S282 OR S283 OR S284 OR S285 OR S286 OR S287 OR S288 OR S289 OR S290 OR S291 OR S292 OR S293 OR S294 OR S295 OR S296 OR S297 OR S298 OR S299 OR S300 OR S301 OR S302 OR S303 OR S304 OR S305 OR S306 OR S307 OR S308 OR S309 OR S310 OR S311 OR S312 OR S313 OR

	S314 OR S315 OR S316 OR S317 OR S318 OR S319 OR S320 OR S321 OR S322 OR S323 OR S324 OR S325 OR S326 OR S327 OR S328 OR S329 OR S330 OR S331 OR S332 OR S333 OR S334 OR S335 OR S336 OR S337 OR S338 OR S339 OR S340 OR S341 OR S342 OR S343 OR S344 OR S345 OR S346 OR S347 OR S348 OR S349 OR S350 OR S351 OR S352 OR S353 OR S354 OR S355 OR S356 OR S357 OR S358 OR S359 OR S360 OR S361 OR S362 OR S363 OR S364 OR S365 OR S366 OR S367 OR S368 OR S369
**S371**	TI LAMIC OR AB LAMIC OR TI LAMICs OR AB LAMICs
**S372**	TI LMIC OR AB LMIC OR TI LMICs OR AB LMICs
**S373**	TI "LAMI Country" OR AB "LAMI Country" OR TI "LAMI Countries" OR AB "LAMI Countries"
**S374**	TI "LMI Country" OR AB "LMI Country" OR TI "LMI Countries" OR AB "LMI Countries"
**S375**	TI "third world" OR AB "third world"
**S376**	(TI developing OR TI "less developed" OR TI "under developed" OR underdeveloped OR TI "least developed" OR TI crisis OR TI war OR TI "middle income" OR TI "low income" OR TI poor) N2 (TI country OR TI countries OR TI nation OR TI nations OR TI area OR TI areas OR TI economy OR TI economies OR TI population OR TI populations)
**S377**	DE "Developing Countries"
**S378**	DE "Human Migration"
**S379**	DE "Refugees"
**S380**	DE "Immigration"
**S381**	TI BME OR TI BEM OR TI BAME OR TI afro
**S382**	TI "black and minority ethnic" OR TI "black and ethnic minority" OR TI "black, Asian and minority ethnic"
**S383**	TI Kurdish OR TI Kurd OR TI Kurds OR TI Kurdistan
**S384**	TI Amerindian OR TI Amerindians
**S385**	TI Hispanic OR TI Hispanics
**S386**	TI Yezidi OR TI Yezidis OR TI Yazidi OR TI Yazidis
**S387**	TI Inuit OR TI Inuits OR TI "first nations" OR TI indigenous
**S388**	TI Eskimo OR TI Eskimos
**S389**	TI Kalaallit OR TI Kalaallits
**S390**	TI Romany OR TI Romanies
**S391**	TI Gypsies OR TI Gipsies OR TI Gypsy OR TI Gipsy
**S392**	TI Aborigine OR TI Aborigines
**S393**	TI Refugee OR TI refugees
**S394**	TI "Asylum seeker" OR TI "Asylum seekers"
**S395**	TI Migrant OR TI Migrants
**S396**	TI immigrant OR TI immigrants
**S397**	TI emigrant OR TI emigrants
**S398**	TI (person OR persons OR people OR group OR groups)
**S399**	(TI displaced) N S398
**S400**	S371 OR S372 OR S373 OR S374 OR S375 OR S376 OR S377 OR S378 OR S379 OR S380
**S401**	S381 OR S382 OR S383 OR S384 OR S385 OR S386 OR S387 OR S388 OR S389 OR S390 OR S391 OR S392
**S402**	S393 OR S394 OR S395 OR S396 OR S397 OR S399
**S403**	S400 OR S401 OR S402

**S404**	S178 OR S370 OR S403
**S405**	DE "Mental Disorders"
**S406**	DE "Chronic Mental Illness"
**S407**	DE "Behavior Disorders"
**S408**	DE "Emotional Adjustment" OR DE "Emotional Disturbances"
**S409**	DE "Emotional Disturbances"
**S410**	DE "Psychiatric Patients"
**S411**	DE "Psychiatric Symptoms"
**S412**	DE "Psychopathology"
**S413**	DE "Thought Disturbances"
**S414**	DE "Psychological Stress"
**S415**	DE "Psychological Endurance"
**S416**	DE "Behavior Problems"
**S417**	DE "Mental Health"
**S418**	TI “mental distress“ OR AB “mental distress“ OR TI “mental stress” OR AB “mental stress” OR TI “mentally stressed” OR AB “mentally stressed”
**S419**	TI “mental issue” OR AB “mental issue” OR TI “mental issues” OR AB “mental issues”
**S420**	TI “mental disturbance” OR AB “mental disturbance” OR TI “mental disturbances” OR AB “mental disturbances” OR TI “mentally disturbed” OR AB “mentally disturbed”
**S421**	TI “mental problem” OR AB “mental problem” OR TI “mental problems” OR AB “mental problems”
**S422**	TI “mental disorder” OR AB “mental disorder” OR TI “mental disorders” OR AB “mental disorders”
**S423**	TI “mental disease” OR AB “mental disease” OR TI “mental diseases” OR AB “mental diseases”
**S424**	TI “mental illness” OR AB “mental illness” OR TI “mental illnesses” OR AB “mental illnesses” OR TI “mentally ill” OR AB “mentally ill”
**S425**	TI “psychiatric disorder” OR AB “psychiatric disorder” OR TI “psychiatric disorders” OR AB “psychiatric disorders”
**S426**	TI “psychiatric disease” OR AB “psychiatric disease” OR TI “psychiatric diseases” OR AB “psychiatric diseases”
**S427**	TI “psychiatric illness” OR AB “psychiatric illness” OR TI “psychiatric illnesses” OR AB “psychiatric illnesses”
**S428**	TI “psychological illness” OR AB “psychological illness” OR TI “psychological illnesses” OR AB “psychological illnesses” OR TI “psychologically ill” OR AB “psychologically ill” OR TI “psychically ill” OR AB “psychically ill”
**S429**	TI “psychological disorder” OR AB “psychological disorder” OR TI “psychological disorders” OR AB “psychological disorders”
**S430**	TI “psychological disease” OR AB “psychological disease” OR TI “psychological diseases” OR AB “psychological diseases”
**S431**	TI “emotional strains” OR AB “emotional strains” OR TI “emotional strain” OR AB “emotional strain” OR TI “emotionally strained” OR AB “emotionally strained”
**S432**	TI “emotional stress” OR AB “emotional stress” OR TI “emotional distress” OR AB “emotional distress” OR TI “emotionally stressed” OR AB “emotionally stressed”
**S433**	TI “emotional disturbance” OR AB “emotional disturbance” OR TI “emotional disturbances” OR AB “emotional disturbances” OR TI “emotionally disturbed” OR AB “emotionally disturbed”
**S434**	TI “emotional damage” OR AB “emotional damage”
**S435**	TI “psychically disturbed” OR AB “psychically disturbed”
**S436**	TI “psychic strain” OR AB “psychic strain” OR TI “psychic strains” OR AB “psychic strains” OR TI “psychically strained” OR AB “psychically strained”

**S437**	TI “psychic distress” OR AB “psychic distress” OR TI “psychic stress” OR AB “psychic stress” OR TI “psychically stressed” OR AB “psychically stressed”
**S438**	TI “Psychological distress” OR AB “Psychological distress” OR TI “Psychological stress” OR AB “Psychological stress” OR TI “Psychologically stressed” OR AB “Psychologically stressed”
**S439**	TI “Psychological strain” OR AB “Psychological strain” OR TI “Psychological strains” OR AB “Psychological strains” OR TI “Psychologically strained” OR AB “Psychologically strained”
**S440**	TI “mental ill-health” OR AB “mental ill-health” OR TI “psychological ill-health” OR AB “psychological ill-health”
**S441**	TI “mental health” OR AB “mental health” OR TI “mental health” OR AB “mental health”
**S442**	TI “mental well being” OR AB “mental well being” OR TI “mental wellbeing” OR AB “mental wellbeing”
**S443**	TI “psychological well being” OR AB “psychological well being” OR TI “psychological wellbeing” OR AB “psychological wellbeing”
**S444**	S405 OR S406 OR S407 OR S408 OR S409 OR S410 OR S411 OR S412 OR S413 OR S414 OR S415 OR S416 OR S417 OR S418 OR S419 OR S420 OR S421 OR S422 OR S423 OR S424 OR S425 OR S426 OR S427 OR S428 OR S429 OR S430 OR S431 OR S432 OR S433 OR S434 OR S435 OR S436 OR S437 OR S438 OR S439 OR S440 OR S441 OR S442 OR S443
**S445**	DE "Adjustment Disorders"
**S446**	DE "Affective Disorders"
**S447**	DE "Bipolar Disorder"
**S448**	DE "Cyclothymic Personality"
**S449**	DE "Disruptive Mood Dysregulation Disorder"
**S450**	DE "Major Depression"
**S451**	DE "Dysthymic Disorder"
**S452**	DE "Mania"
**S453**	DE "Hypomania"
**S454**	DE "Seasonal Affective Disorder"
**S455**	DE “anhedonia”
**S456**	TI "Bipolar Disorder" OR AB "Bipolar Disorder" OR TI "Bipolar Disorders" OR AB "Bipolar Disorders"
**S457**	TI "Mood Disorder" OR AB "Mood Disorder" OR TI "Mood Disorders" OR AB "Mood Disorders"
**S458**	TI "Cyclothymic Disorder" OR AB "Cyclothymic Disorder" OR TI "Cyclothymic Disorders" OR AB "Cyclothymic Disorders" OR TI "premenstrual dysphoric disorder" OR AB "premenstrual dysphoric disorder" OR TI "premenstrual dysphoric disorders" OR AB "premenstrual dysphoric disorders"
**S459**	TI "affective Disorder" OR AB "affective Disorder" OR TI "affective Disorders" OR AB "affective Disorders"
**S460**	TI depression OR AB depression OR TI depressions OR AB depressions OR TI depressed OR AB depressed OR TI depressive OR AB depressive
**S461**	TI dysthymic OR AB dysthymic OR TI dysthymia OR AB dysthymia OR TI melancholy OR AB melancholy OR TI melancholic OR AB melancholic OR TI melancholia OR AB melancholia
**S462**	S445 OR S446 OR S447 OR S448 OR S449 OR S450 OR S451 OR S452 OR S453 OR S454 OR S455 OR S456 OR S457 OR S458 OR S459 OR S460 OR S461
**S463**	DE "Anxiety Disorders"
**S464**	DE "Acute Stress Disorder"
**S465**	DE "Generalized Anxiety Disorder"
**S466**	DE "Obsessive Compulsive Disorder"

**S467**	DE "Panic Disorder"
**S468**	DE "Phobias"
**S469**	DE "Acrophobia"
**S470**	DE "Agoraphobia"
**S471**	DE "Claustrophobia"
**S472**	DE "Social Phobia"
**S473**	DE "Separation Anxiety Disorder"
**S474**	DE “anxiety”
**S475**	DE “Fear”
**S476**	DE “Panic”
**S477**	DE "Panic Attack"
**S478**	TI agoraphobia OR AB agoraphobia
**S479**	TI Anxiety OR AB Anxiety
**S480**	TI Neurotic OR AB Neurotic
**S481**	TI "Obsessive-Compulsive Disorder" OR AB "Obsessive-Compulsive Disorder" OR TI "Obsessive-Compulsive Disorders" OR AB "Obsessive-Compulsive Disorders"
**S482**	TI phobic OR AB phobic OR TI phobia OR AB phobia
**S483**	TI panic OR AB panic OR TI "selective mutism" OR AB "selective mutism"
**S484**	S463 OR S464 OR S465 OR S466 OR S467 OR S468 OR S469 OR S470 OR S471 OR S472 OR S473 OR S474 OR S475 OR S476 OR S477 OR S478 OR S479 OR S480 OR S481 OR S482 OR S483
**S485**	DE "Post-Traumatic Stress"
**S486**	DE "Posttraumatic Stress Disorder"
**S487**	DE "Complex PTSD"
**S488**	DE "Emotional Trauma"
**S489**	TI trauma OR AB trauma OR TI traumatised OR AB traumatised OR TI traumatized OR AB traumatized
**S490**	TI PTSD OR AB PTSD OR TI "stress disorder" OR AB "stress disorder" OR TI "stress disorders" OR AB "stress disorders"
**S491**	TI "post traumatic" OR AB "post traumatic" OR TI posttraumatic OR AB posttraumatic OR TI traumatic OR AB traumatic
**S492**	TI "Adjustment Disorders" OR AB "Adjustment Disorders" OR TI "Adjustment Disorder" OR AB "Adjustment Disorder"
**S493**	S485 OR S486 OR S487 OR S488 OR S489 OR S490 OR S491 OR S492
**S494**	DE "Autism Spectrum Disorders"
**S495**	DE "Dissociative Disorders"
**S496**	DE "Depersonalization"
**S497**	DE "Depersonalization/Derealization Disorder"
**S498**	DE "Dissociative Identity Disorder"
**S499**	DE "Dissociation"
**S500**	TI "Dissociative Disorder" OR AB "Dissociative Disorder" OR TI "Dissociative Disorders" OR AB "Dissociative Disorders"
**S501**	TI Depersonalisation OR AB Depersonalisation OR TI Depersonalization OR AB Depersonalization
**S502**	TI Dissociation OR AB Dissociation
**S503**	S494 OR S495 OR S496 OR S497 OR S498 OR S499 OR S500 OR S501 OR S502
**S504**	DE "Impulse Control Disorders"

**S505**	DE "Impulsiveness"
**S506**	DE "Kleptomania"
**S507**	DE "Pathological Gambling"
**S508**	DE "Trichotillomania"
**S509**	DE "Hoarding Disorder"
**S510**	DE "Hoarding Behavior"
**S511**	TI "Impulse Control Disorder" OR AB "Impulse Control Disorder" OR TI "Impulse Control Disorders" OR AB "Impulse Control Disorders”
**S512**	TI "hoarding behavior" OR AB "hoarding behavior" OR TI "hoarding disorder" OR AB "hoarding disorder"
**S513**	TI gambling OR AB gambling
**S514**	TI Trichotillomania OR AB Trichotillomania
**S515**	S504 OR S505 OR S506 OR S507 OR S508 OR S509 OR S510 OR S511 OR S512 OR S513 OR S514
**S516**	DE "Eating Disorders"
**S517**	DE "Anorexia Nervosa"
**S518**	DE "Binge Eating Disorder"
**S519**	DE "Bulimia"
**S520**	DE "Purging (Eating Disorders)“
**S521**	DE "Binge Eating"
**S522**	TI "Eating Disorder" OR AB "Eating Disorder" OR TI "Eating Disorders" OR AB "Eating Disorders"
**S523**	TI Bulimia OR AB Bulimia
**S524**	TI Anorexia OR AB Anorexia
**S525**	TI "Binge Eating" OR AB "Binge Eating"
**S526**	S516 OR S517 OR S518 OR S519 OR S520 OR S521 OR S522 OR S523 OR S524 OR S525
**S527**	DE "Personality Disorders"
**S528**	DE "Borderline States"
**S529**	DE "Obsessive Compulsive Personality Disorder"
**S530**	DE "Histrionic Personality Disorder"
**S531**	DE "Neurosis"
**S532**	DE "Antisocial Personality Disorder"
**S533**	DE "Avoidant Personality Disorder"
**S534**	DE "Borderline Personality Disorder"
**S535**	DE "Dependent Personality Disorder"
**S536**	DE "Narcissistic Personality Disorder"
**S537**	DE "Paranoid Personality Disorder"
**S538**	DE "Passive Aggressive Personality Disorder"
**S539**	DE "Schizoid Personality Disorder"
**S540**	DE "Schizotypal Personality Disorder"
**S541**	TI "Personality Disorder" OR AB "Personality Disorder" OR TI "Personality Disorders" OR AB "Personality Disorders"
**S542**	TI Borderline OR AB Borderline
**S543**	TI histrionic OR AB histrionic
**S544**	TI Paranoid OR AB Paranoid

**S545**	TI schizoid OR AB schizoid
**S546**	TI Schizotypal OR AB Schizotypal
**S547**	TI narcism OR AB narcism
**S548**	DE "Psychosis"
**S549**	DE "Affective Psychosis"
**S550**	DE "Schizophrenia"
**S551**	DE "Paranoia (Psychosis)"
**S552**	DE "Paranoid Schizophrenia"
**S553**	DE "Schizoaffective Disorder"
**S554**	TI Schizophrenia OR AB Schizophrenia
**S555**	TI Psychotic OR AB Psychotic
**S556**	TI psychosis OR AB psychosis OR TI psychoses OR AB psychoses
**S557**	TI schizoaffective OR AB schizoaffective
**S558**	TI schizophrenic OR AB schizophrenic
**S559**	TI "Multiple Personality" OR AB "Multiple Personality" OR TI "Multiple Personalities" OR AB "Multiple Personalities"
**S560**	S527 OR S528 OR S529 OR S530 OR S531 OR S532 OR S533 OR S534 OR S535 OR S536 OR S537 OR S538 OR S539 OR S540 OR S541 OR S542 OR S543 OR S544 OR S545 OR S546 OR S547 OR S548 OR S549 OR S550 OR S551 OR S552 OR S553 OR S554 OR S555 OR S556 OR S557 OR S558 OR S559
**S561**	DE "Sleep Disorders"
**S562**	DE "Hypersomnia"
**S563**	DE "Insomnia"
**S564**	DE "Parasomnias"
**S565**	DE "Sleep Apnea"
**S566**	DE "Sleep Deprivation"
**S567**	DE “sleep”
**S568**	TI "Sleep Wake Disorder" OR AB "Sleep Wake Disorder" OR TI "Sleep Wake Disorders" OR AB "Sleep Wake Disorders"
**S569**	TI "Sleep Disorder" OR AB "Sleep Disorder" OR TI "Sleep Disorders" OR AB "Sleep Disorders"
**S570**	TI "Sleeping Disorder" OR AB "Sleeping Disorder" OR TI "Sleeping Disorders" OR AB "Sleeping Disorders"
**S571**	TI "Sleeping problem" OR AB "Sleeping problem" OR TI "Sleeping problems" OR AB "Sleeping problems"
**S572**	TI "sleep problem" OR AB "sleep problem" OR TI "sleep problems" OR AB "sleep problems"
**S573**	TI Dyssomnia OR AB Dyssomnia
**S574**	TI Parasomnia OR AB Parasomnia
**S575**	TI insomnia OR AB insomnia
**S576**	TI "restless legs" OR AB "restless legs"
**S577**	S561 OR S562 OR S563 OR S564 OR S565 OR S566 OR S567 OR S568 OR S569 OR S570 OR S571 OR S572 OR S573 OR S574 OR S575 OR S576
**S578**	DE "Toxic Disorders"
**S579**	DE "Substance Use Disorder"
**S580**	DE "Alcohol Intoxication"
**S581**	DE "Alcoholism"

**S582**	TI "Substance-Related Disorder" OR AB "Substance-Related Disorder" OR TI "Substance-Related Disorders" OR AB "Substance-Related Disorders"
**S583**	TI "Alcohol-Related Disorder" OR AB "Alcohol-Related Disorder" OR TI "Alcohol-Related Disorders" OR AB "Alcohol-Related Disorders"
**S584**	TI alcoholism OR AB alcoholism
**S585**	TI "alcohol dependence" OR AB "alcohol dependence"
**S586**	TI "alcohol dependency" OR AB "alcohol dependency"
**S587**	TI "alcohol abuse" OR AB "alcohol abuse"
**S588**	TI "substance abuse" OR AB "substance abuse"
**S589**	TI "substance dependence" OR AB "substance dependence"
**S590**	TI "substance dependency" OR AB "substance dependency"
**S591**	TI addiction OR AB addiction
**S592**	S578 OR S579 OR S580 OR S581 OR S582 OR S583 OR S584 OR S585 OR S586 OR S587 OR S588 OR S589 OR S590 OR S591
**S593**	TI "somatic symptom disorder" OR AB "somatic symptom disorder" OR TI "somatic symptom disorders" OR AB "somatic symptom disorders"
**S594**	TI somatization OR AB somatization OR TI somatisation OR AB somatisation
**S595**	TI Somatoform OR AB Somatoform
**S596**	S593 OR S594 OR S595
**S597**	S 462 OR S484 OR S493 OR S503 OR S515 OR S526 OR S560 OR S577 OR S592 OR S596
**S598**	DE “Health Behavior”
**S599**	DE “Health Promotion"
**S600**	DE "Contraceptive Devices"
**S601**	DE "Risk Factors"
**S602**	DE "Protective Factors"
**S603**	DE "Self-Examination (Medical)"
**S604**	DE "Treatment Compliance"
**S605**	DE "Meditation"
**S606**	DE "Safe Sex"
**S607**	DE "Social Behavior"
**S608**	DE "Health Education"
**S609**	DE "Lifestyle"
**S610**	S598 OR S599 OR S600 OR S601 OR S602 OR S603 OR S604 OR S605 OR S606 OR S607 OR S608 OR S609
**S611**	TI nutrition OR AB Nutrition
**S612**	TI "body weight" OR AB "body weight"
**S613**	TI "weight reduction" OR AB "weight reduction"
**S614**	TI Diet OR AB Diet
**S615**	TI "weight loss" OR AB "weight loss"
**S616**	TI "Food Intake" OR AB "Food Intake"
**S617**	TI "Food consumption" OR AB "Food consumption"
**S618**	TI "Nutritional Status" OR AB "Nutritional Status"
**S619**	TI "Food preferences" OR AB "Food preferences"

**S620**	TI "Food Habits" OR AB "Food habits"
**S621**	S611 OR S612 OR S613 OR S614 OR S615 OR S616 OR S617 OR S618 OR S619 OR S620
**S622**	TI Exercise OR AB Exercise
**S623**	TI sport OR AB sport OR TI sport OR AB sport
**S624**	TI "Physical Fitness" OR AB "Physical Fitness"
**S625**	TI "Physical Education" OR AB "Physical Education"
**S626**	TI "Physical exertion" OR AB "Physical exertion"
**S627**	TI "Movement therapy" OR AB "Movement therapy"
**S628**	S622 OR S623 OR S624 OR S625 OR S626 OR S627
**S629**	TI "Health Education" OR AB "Health Education"
**S630**	TI "Disease Control" OR AB "Disease Control"
**S631**	TI Preven* OR AB Preven*
**S632**	TI "Healthy People Program" OR AB "Healthy People Program"
**S633**	TI "Patient Satisfaction" OR AB "Patient Satisfaction"
**S634**	TI "wellness program" OR AB "wellness program"
**S635**	TI "Obesity Management" OR AB "Obesity Management"
**S636**	TI "sleep hygiene" OR AB "sleep hygiene"
**S637**	TI "Self Care" OR AB "Self Care"
**S638**	TI "Self-Care" OR AB "Self-Care"
**S639**	TI "Patient adherence" OR AB "Patient adherence"
**S640**	TI "Patient Compliance" OR AB "Patient Compliance"
**S641**	S629 OR S630 OR S631 OR S632 OR S633 OR S634 OR S635 OR S636 OR S637 OR S638 OR S639 OR S640
**S642**	S610 OR S621 OR S628 OR S641
**S643**	S444 OR S597 OR S642
**S644**	S95 AND S404 AND S643

Supplementary Table 3: Details on the original and culturally adapted internet- and mobile-based interventions (IMI) used in the included articles

1st author (year)	Name	Language	Target group	Ethnicity	Mental disorder	Therapy approach (adapted IMI)	Website vs. app/ guidance (adapted IMI)	Duration/ No. modules (adapted IMI)
	original IMI	original IMI	original IMI	original IMI	original IMI			
	adapted IMI	adapted IMI	adapted IMI	adapted IMI	adapted IMI			
Abi Ramia (2018)[1] Harper Shehadeh (2020)[2]	Step-by-Step [3]	English	immigrants, general population	various ethnicities	mixed			
	Step-by-Step for Lebanon	Arabic, English	immigrants, general population	Lebanese, Syrian, Palestinian	depression	(C)BT	Website with guidance	5 modules
Abuwalla (2017)[4]	CATCH-IT[5]	English	adolescents	American	depression			
	CATCH-IT for Arab adolescents [a]	Arabic	adolescents	Arabic	depression	(C)BT, IPT	Website without guidance	14 modules
Arjadi (2018a+b)[6, 7]	Doe en Voel (=Act and Feel) [8]	Dutch	general population	Dutch	depression			
	Guided Act and Feel Indonesia	Indonesian	general population	Indonesian ethnicities	depression	(C)BT	Website with guidance	10 weeks 8 modules
Bolinski (2018)[9]	ICare Prevent [10]	German	general population	German	depression, anxiety			
	ICare Prevent	Dutch	students	Dutch	mixed	CBT	Website with/ without guidance	7 weeks 8 modules
Brooks (2013)[11]	-	English	veterans, patients	American	traumata			
	American Indian PTSD Core Module	English	indigenous veterans/ patients	American Indian	traumata	-	Remote monitoring with guidance	90 days 16 modules

1st author (year)	Name<br>original IMI<br>adapted IMI	Language<br>original IMI<br>adapted IMI	Target group<br>original IMI<br>adapted IMI	Ethnicity<br>original IMI<br>adapted IMI	Mental disorder<br>original IMI<br>adapted IMI	Therapy approach (adapted IMI)	Website vs. app/ guidance (adapted IMI)	Duration/ No. modules (adapted IMI)
Burchert (2018)[12]	Step-by-Step for Lebanon [1]	Arabic	immigrants, general population	Lebanese, Syrian, Palestinian	depression			
	Step-by-Step for Syrian refugees	Arabic	immigrants (refugees)	Syrian, Arabic	stress, impaired functioning	(C)BT	Website/ app with guidance	5 weeks
Campbell (2015)[13]	Community reinforcement approach [14]	English	general population	American	substance abuse			
	Therapeutic Education System [a]	English	indigenous	American Indian /Alaska Natives	substance abuse	community reinforcem ent approach	Website without guidance	8 weeks<br>32 modules
Chen (2019)[15]<br>Lin (2020)[16]	Internet-based self-help for social phobia [17]	German	general population	Swiss	social anxiety			
	ICBT for social anxiety in China	Chinese	general population	Chinese	social anxiety	(C)BT	Website with guidance	8 weeks<br>8 modules
Choi (2012)[18]	Sadness iCBT Program [19]	English	general population	Australian	depression			
	Brighten Your Mood Program	English, Chinese	immigrants	Chinese Australians	depression	(C)BT	Website with guidance	8 weeks<br>6 modules
DaPonte (2018)[20]	Wellbeing Course [21]	English	general population	Australian, Anglo-Canadian	depression, anxiety			
	Cours Mieux-être	French	general population	French-Canadian	depression, anxiety	CBT	Website with guidance	8 weeks<br>5 modules

1st author (year)	Name	Language	Target group	Ethnicity	Mental disorder	Therapy approach (adapted IMI)	Website vs. app/ guidance (adapted IMI)	Duration/ No. modules (adapted IMI)
	original IMI	original IMI	original IMI	original IMI	original IMI			
	adapted IMI	adapted IMI	adapted IMI	adapted IMI	adapted IMI			
Eylem (2020)[22]	[23]	Dutch	general population	Dutch	suicidal ideation			
	-	English, Dutch, Turkish	immigrants	Turkish(-Dutch/-English)	suicidal ideation	CBT, PST	Website/ app with guidance	6 weeks 6 modules
Garabiles (2019)[24]	Step-by-Step general [3]/ for Lebanon [1]	English, Arabic	immigrants, general population	Arabic (in Lebanon)	depression			
	Step-by-Step for overseas Filipino workers	English, Filipino	labour migrants	Filipino	general mental health	(C)BT	Website with guidance	6 weeks 6 modules
Gorman (2013)[25]	Web-based Screening, Brief Intervention and Referral for Treatment [26]	English	women in childbearing age	American	-			
	-	English	indigenous women in childbearing age	American Indian/ Alaska Native	-	BT	Website with guidance	-
Hiratsuka (2019)[27]	PTSD Coach Online [28]	English	veterans	American	traumata, stress			
	Health is Our Tradition: Balance and Harmony after Trauma	English	indigenous	American Indian/ Alaska Native	traumata	(C)BT	Website, app without guidance	12 weeks 16 modules
Imamura (2019)[29]	Internet CBT program: Useful	Japanese	workers	Japanese	stress	CBT	App with guidance	10 weeks

**1st author (year)**	**Name**<br>original IMI<br>adapted IMI	**Language**<br>original IMI<br>adapted IMI	**Target group**<br>original IMI<br>adapted IMI	**Ethnicity**<br>original IMI<br>adapted IMI	**Mental disorder**<br>original IMI<br>adapted IMI	**Therapy approach** (adapted IMI)	**Website vs. app/ guidance** (adapted IMI)	**Duration/ No. modules** (adapted IMI)
	mental health solutions series for business [30]							6 modules
	-	Vietnamese	workers (nurses)	Vietnamese	depression, anxiety			
Ip (2016)[31]	CATCH-IT [5]	English	adolescents	American	depression			~ 8 months
Sobowale (2013)[32]	Grasp the Opportunity	Chinese	adolescents	(Hong Kong) Chinese	depression, traumata, stress, substance abuse	CBT	Website with guidance	10 modules
Juniar (2019)[33]	GET.ON Stress [34]	German	workers	German	stress			6 weeks
	Rileks (=Relax)	Indonesian	students	Indonesian	stress	PST/ emotional therapy	Website with guidance	6 modules
Kaal (2020)[35]	Takecareofyou [36]	German	general population	Swiss	alcohol abuse			10 weeks
	Selge	Estonian, Russian	general population	Estonian	alcohol abuse	(C)BT, motivational interviewing	Website with guidance	8 modules
Kanuri (2020)[37]	Lantern Anxiety Program [38]	English	general population	Indian	anxiety			~4 weeks
	Mana Maali Digital Anxiety Program (Gardener of the Mind)	English	students	Indian	anxiety	CBT	Website without guidance	20 modules

1st author (year)	Name	Language	Target group	Ethnicity	Mental disorder	Therapy approach (adapted IMI)	Website vs. app/ guidance (adapted IMI)	Duration/ No. modules (adapted IMI)
	original IMI	original IMI	original IMI	original IMI	original IMI			
	adapted IMI	adapted IMI	adapted IMI	adapted IMI	adapted IMI			
Kayrouz (2015/ 2016a+b)[39–41]	Wellbeing Course [21]	English	general population	Australian	depression, anxiety	(C)BT, IPT	Website with guidance	8 weeks 5 modules
	Arab Wellbeing Course	English, Arabic	immigrants	Arabic	depression, anxiety			
Knaevelsrud (2015)[42] Vöhringer (2020)[43] Wagner (2012)[44]	Interapy [45]	Dutch	general population	Dutch	traumata	CBT	Website with guidance	5 weeks 10 modules
	Ilanjafsy	Arabic	general population	Arabic	traumata, depression, anxiety			
Lal (2020)[46]	Horyzons [47]	English	patients	Australian	psychotic disorder	(C)BT, psycho-social therapy	Website with guidance	-
	Horyzons Canada	English	patients	Canadian	psychotic disorder			
Lindegaard (2020)[48] Nygren (2018)[49]	(mix of interventions)	English, Swedish	-	-	depression, aggressive behaviour	CBT	Website without guidance	8 weeks 9 modules
	-	+ Arabic	immigrants (refugees)	Arabic	depression, anxiety, sleep, stress			
Luo (2020)[50]	eBody Project [51]	English	adolescents	American	eating disorders	CBT	Website with guidance	6 weeks, 6 modules
	eBody Project – Chinese version	Chinese	young women	Chinese	eating disorders			

**1st author (year)**	**Name**	**Language**	**Target group**	**Ethnicity**	**Mental disorder**	**Therapy approach** (adapted IMI)	**Website vs. app/ guidance** (adapted IMI)	**Duration/ No. modules** (adapted IMI)
	original IMI	original IMI	original IMI	original IMI	original IMI			
	adapted IMI	adapted IMI	adapted IMI	adapted IMI	adapted IMI			
Muroff (2017/ 2019)[52,53]	A-CHESS [54]	English	patients (after residential treatment)	American	substance abuse	CBT, self-determination therapy	App without guidance	unlimited
	CASA-CHESS	Spanish	immigrated patients (after residential treatment)	Latino / Hispanic (-American)	substance abuse			
Nygren (2019)[55]	iCBT for depression [56]	Swedish	general population	Swedish	depression	CBT	Website with guidance	8 weeks<br>7 modules
	-	Kurdish (Sorani)	immigrants	Kurdish	depression			
Okujava (2019)[57]	i-Sleep [58]	Dutch	patients	Dutch	sleep	CBT	Website with guidance	6 weeks<br>5 modules
	DCBT for insomnia	Georgian	patients	Georgian	sleep			
Paris (2018)[59]	CBT4CBT [62]	English	patients, general population	American	substance abuse	CBT	Website without guidance	8 weeks<br>7 modules
Silva (in prep)[60]<br>Silva (2020)[61]	CBT4CBT Spanish	Spanish	immigrated patients	Latino(-American)	substance abuse			
Patel (2016)[63]	CATCH-IT [5]	English	adolescents	American	depression	(C)BT, PST, IPT	Website without guidance	8 weeks<br>16 modules
	Yia You (Keep going) [a]	Chinese	adolescents	Chinese	depression			

1st author (year)	Name<br>original IMI<br>adapted IMI	Language<br>original IMI<br>adapted IMI	Target group<br>original IMI<br>adapted IMI	Ethnicity<br>original IMI<br>adapted IMI	Mental disorder<br>original IMI<br>adapted IMI	Therapy approach (adapted IMI)	Website vs. app/ guidance (adapted IMI)	Duration/ No. modules (adapted IMI)
Pinto-Bruno (2019)[64]	iSupport (Pot et al., 2019)	English	caregivers (unpaid)	Indian	stress, mental health problems	(C)BT, PST	Website with guidance	~ 3 months<br>5 modules
	iSupport	Dutch	caregivers (unpaid)	Dutch	stress, mental health problems			
Rahmadiana (2019)[66]	ICare Prevent [10]	German	students	German, Swiss	depression, anxiety	BT	Website/ app with guidance	7 weeks<br>7 modules
	I-AiMental WELLness (Saya menuju mental sehat)	Indonesian	students	Indonesian	depression, anxiety			
Salamanca-Sanabria (2019/ 2020)[67,68]	Space from depression [69]	English	general	Irish	depression	CBT	Website with guidance	7 weeks<br>7 modules
	Yo puedo sentirme bien (I can feel better)	Spanish	students	Colombian	depression			
Saulsberry (2013)[70]	CATCH-IT [5]	English	adolescents	American	depression	(C)BT, IPT, community resilience concept	Website with guidance	14 modules
	CURB (Chicago Urban Resiliency Buidling)	English, Spanish	immigrated adolescents	Latino-American, African-American	depression			
Shala (2020)[71]	Step-by-Step [3]	English	immigrants, general population	various ethnicities	mixed	(C)BT	Website with guidance	5 modules
	Hap-pas-Hapi	Albanian	immigrants	Albanian	stress			

1st author (year)	Name<br>original IMI<br>adapted IMI	Language<br>original IMI<br>adapted IMI	Target group<br>original IMI<br>adapted IMI	Ethnicity<br>original IMI<br>adapted IMI	Mental disorder<br>original IMI<br>adapted IMI	Therapy approach (adapted IMI)	Website vs. app/ guidance (adapted IMI)	Duration/ No. modules (adapted IMI)
Sit (2020)[72]	Step-by-Step [3]	English	immigrants, general population	various ethnicities	mixed			
	SbS for Chinese young adults	Chinese	young adults	Chinese	depression, anxiety	(C)BT	Website with guidance	5 modules
Spanhel (2019)[73]	GET.ON Recovery [74]	German	workers	German	sleep			
	eSano Sleep-e [a]	English, German	immigrants (refugees)	Ethnicities from war-/ conflict countries	sleep	CBT	Website/ app without guidance	3 weeks<br>4 modules
Teles (2020)[75]	iSupport (Pot et al., 2019)	English	caregivers (unpaid)	Indian	stress, mental health problems			
	iSupport for dementia – European-Portuguese version	Portugues e	caregivers (unpaid)	Portuguese	stress, mental health problems	(C)BT, PST	Website with guidance	~ 3 months<br>5 modules
Titov (2018)[76]	Wellbeing Course [21]	English	general population	Australian	depression, anxiety			
	Indigenous Wellbeing Course	English	indigenous	Native-Australians	depression, anxiety	CBT, IPT	Website with guidance	8 weeks<br>6 modules
Ünlü Ince (2013)[77]	Alles Onder Control [78]	Dutch	general population	Dutch	depression			
	Alles Onder Control Turks	Dutch, Turkish	immigrants	Turkish (-Dutch)	depression	PST	Website with guidance	5 weeks<br>5 modules
Wang (2013)[79]	My Disaster Recovery [80]	English	general population	American	traumata			4 weeks

1st author (year)	Name<br>original IMI<br>adapted IMI	Language<br>original IMI<br>adapted IMI	Target group<br>original IMI<br>adapted IMI	Ethnicity<br>original IMI<br>adapted IMI	Mental disorder<br>original IMI<br>adapted IMI	Therapy approach (adapted IMI)	Website vs. app/ guidance (adapted IMI)	Duration/ No. modules (adapted IMI)
	Chinese My Trauma Recovery	Chinese	general population	China	traumata	social cognitive therapy	Website with/ without guidance	6 modules
Wasil (2020)[81]	Growth mindset intervention [82]	English	pupils, adolescents	American	depression, anxiety	CBT	Website without guidance	1 day<br>1 module
	Growth mindset intervention	English	pupils, adolescents	Indian	general mental health			
Yokomitsu (2020)[83]	SPARX [84]	English	pupils	New Zealander	depression	(C)BT	App without guidance	12 weeks<br>7 modules
	SPARX Japanese Version	Japanese	students	Japanese	depression			

(C)BT (cognitive) behaviour therapy, *IPT* interpersonal therapy, *PST* problem solving therapy.

[a] Cultural adaptation planned but not yet conducted.

Supplementary Table 4: Quality ratings of the included studies using the Quality Assessment Tool for Reviewing Studies with Diverse Designs [85]

1st author (year)	Reviewer	Fram	Aim	Set	Sam	Repr	Coll	Tool	Recr	Rel1	Fit1	Fit2	Fit3	Ana	Rel2	User	Disc	Sum score (max)
Abi Ramia (2018)[1]	R1	3	3	3	0	2	2	3	2	n	n	3	3	2	3	1	3	
	R2	3	3	3	2	3	3	3	3	n	n	3	3	3	3	3	3	**35**
	**Consensus**	**3**	**3**	**3**	**0**	**2**	**3**	**3**	**2**	**n**	**n**	**3**	**3**	**2**	**3**	**2**	**3**	**(42)**
Abuwalla (2017)[4]	R1	3	3	3	0	2	2	1	0	n	n	2	2	0	0	0	2	
	R2	3	3	3	1	1	3	3	2	n	n	3	2	2	3	0	2	**21**
	**Consensus**	**3**	**3**	**3**	**0**	**1**	**2**	**3**	**0**	**n**	**n**	**2**	**2**	**0**	**0**	**0**	**2**	**(42)**
Arjadi (2018a)[6]	R1	3	3	3	0	2	3	2	0	n	n	3	0	0	2	0	2	
	R2	3	3	2	1	2	3	3	1	n	n	3	2	3	3	2	2	**31**
	**Consensus**	**3**	**3**	**2**	**0**	**2**	**3**	**2**	**1**	**n**	**n**	**3**	**2**	**3**	**3**	**2**	**2**	**(42)**
Arjadi (2018b)[86]	R1	3	3	3	3	2	3	2	3	0	3	n	3	3	n	0	3	
	R2	2	3	3	1	2	3	2	3	0	2	n	3	1	n	0	3	**33**
	**Consensus**	**3**	**3**	**3**	**3**	**2**	**3**	**2**	**3**	**0**	**2**	**n**	**3**	**3**	**n**	**0**	**3**	**(42)**
Bolinski (2018)[9]	R1	3	3	3	3	n	3	3	n	n	3	n	3	2	n	0	3	
	R2	3	3	3	3	n	3	3	n	n	3	n	2	2	n	2	3	**29**
	**Consensus**	**3**	**3**	**3**	**3**	**n**	**3**	**3**	**n**	**n**	**3**	**n**	**3**	**2**	**n**	**0**	**3**	**(33)**
Brooks (2013)[11]	R1	3	2	3	0	2	1	1	0	n	n	3	0	0	0	2	2	
	R2	2	3	1	0	1	1	1	0	n	n	2	2	1	2	1	1	**15**
	**Consensus**	**2**	**2**	**1**	**0**	**1**	**1**	**1**	**0**	**n**	**n**	**2**	**1**	**0**	**2**	**1**	**1**	**(42)**
Burchert (2018)[12]	R1	3	3	3	2	3	3	3	3	n	n	3	3	3	3	3	3	
	R2	3	3	2	2	2	3	3	2	n	n	2	2	3	3	3	3	**36**
	**Consensus**	**3**	**3**	**2**	**2**	**2**	**3**	**3**	**2**	**n**	**n**	**2**	**2**	**3**	**3**	**3**	**3**	**(42)**
Campbell (2015)[13]	R1	3	3	3	0	2	3	3	3	1	2	3	3	2	3	2	3	
	R2	3	3	3	1	1	3	3	2	3	2	2	3	1	2	1	3	**37**
	**Consensus**	**3**	**3**	**3**	**1**	**2**	**3**	**3**	**2**	**2**	**2**	**2**	**3**	**1**	**3**	**1**	**3**	**(48)**
Chen (2019)[15]	R1	3	3	2	0	2	2	1	2	3	3	n	3	1	n	0	2	
	R2	3	3	2	0	2	2	2	2	3	3	n	3	2	n	0	3	**28**
	**Consensus**	**3**	**3**	**2**	**0**	**2**	**2**	**1**	**2**	**3**	**3**	**n**	**3**	**1**	**n**	**0**	**3**	**(42)**
Choi (2012)[18]	R1	3	3	3	3	2	3	3	3	3	3	n	3	3	n	1	3	
	R2	2	3	3	3	2	3	3	3	3	3	n	3	3	n	1	3	**37**
	**Consensus**	**2**	**3**	**3**	**3**	**1**	**3**	**3**	**3**	**3**	**3**	**n**	**3**	**3**	**n**	**1**	**3**	**(42)**

1st author (year)	Reviewer	Fram	Aim	Set	Sam	Repr	Coll	Tool	Recr	Rel1	Fit1	Fit2	Fit3	Ana	Rel2	User	Disc	Sum score (max)
DaPonte (2018)[20]	R1	3	3	3	0	2	3	3	3	3	3	n	3	2	n	2	3	
	R2	2	3	3	1	1	3	2	3	2	1	n	2	2	n	1	3	**29**
	**Consensus**	**3**	**3**	**3**	**0**	**1**	**3**	**2**	**3**	**1**	**2**	**n**	**2**	**2**	**n**	**1**	**3**	**(42)**
Eylem (2020)[22]	R1	3	3	3	3	2	2	3	3	0	3	3	3	2	1	2	3	
	R2	3	3	3	3	1	3	3	2	1	3	3	2	2	1	2	2	**40**
	**Consensus**	**3**	**3**	**3**	**3**	**1**	**3**	**3**	**3**	**1**	**3**	**3**	**3**	**2**	**1**	**2**	**3**	**(48)**
Garabiles (2019)[24]	R1	3	3	3	3	2	2	3	3	0	3	3	3	2	1	2	3	
	R2	3	3	3	3	1	3	3	2	1	3	3	2	2	1	2	2	**33**
	**Consensus**	**3**	**3**	**3**	**3**	**1**	**3**	**3**	**3**	**1**	**3**	**3**	**3**	**2**	**1**	**2**	**3**	**(42)**
Gorman (2013)[25]	R1	3	3	2	1	1	3	2	3	n	n	3	3	2	3	2	2	
	R2	3	3	3	1	2	2	2	2	n	n	3	3	3	2	3	3	**34**
	**Consensus**	**3**	**3**	**3**	**1**	**2**	**2**	**2**	**2**	**n**	**n**	**3**	**3**	**2**	**3**	**2**	**3**	**(42)**
Harper Shehadeh (2020)[2]	R1	2	3	3	1	3	3	1	3	1	3	3	3	2	3	3	3	
	R2	3	3	3	1	2	2	1	2	1	3	3	3	2	3	3	3	**38**
	**Consensus**	**2**	**3**	**3**	**1**	**2**	**3**	**1**	**2**	**1**	**3**	**3**	**3**	**2**	**3**	**3**	**3**	**(48)**
Hiratsuka (2019)[27]	R1	3	3	3	0	2	3	2	3	0	3	n	3	2	n	2	3	
	R2	3	3	3	1	2	3	3	1	1	2	n	2	2	n	2	3	**30**
	**Consensus**	**3**	**3**	**3**	**0**	**2**	**3**	**2**	**2**	**0**	**3**	**n**	**2**	**2**	**n**	**2**	**3**	**(42)**
Imamura (2019)[29]	R1	3	3	3	3	n	3	3	n	n	3	n	3	3	n	0	2	
	R2	3	3	3	3	n	2	1	n	n	3	n	3	1	n	2	3	**29**
	**Consensus**	**3**	**3**	**3**	**3**	**n**	**2**	**2**	**n**	**n**	**3**	**n**	**3**	**2**	**n**	**2**	**3**	**(33)**
Ip (2016)[31]	R1	3	3	3	0	2	2	3	3	0	3	n	3	2	n	1	2	
	R2	3	3	3	3	3	3	2	3	2	3	n	3	2	n	0	3	**32**
	**Consensus**	**3**	**3**	**3**	**0**	**3**	**3**	**2**	**3**	**1**	**3**	**n**	**3**	**2**	**n**	**0**	**3**	**(42)**
Juniar (2019)[33]	R1	3	3	3	1	3	3	2	3	n	n	3	2	1	0	2	2	
	R2	3	3	3	2	2	3	3	2	n	n	3	3	3	3	3	2	**36**
	**Consensus**	**3**	**3**	**3**	**2**	**2**	**3**	**3**	**2**	**n**	**n**	**3**	**3**	**2**	**2**	**3**	**2**	**(42)**
Kaal (2020)[35]	R1	3	3	3	3	n	3	2	n	n	3	n	2	0	n	0	1	
	R2	3	3	3	3	n	3	2	n	n	3	n	3	2	n	0	2	**25**
	**Consensus**	**3**	**3**	**3**	**3**	**n**	**3**	**2**	**n**	**n**	**3**	**n**	**3**	**1**	**n**	**0**	**1**	**(33)**
Kanuri (2020)[37]	R1	3	3	1	0	2	2	3	2	1	2	3	3	2	0	2	3	
	R2	2	3	2	1	1	2	3	1	3	3	3	3	2	2	2	3	

1st author (year)	Reviewer	Fram	Aim	Set	Sam	Repr	Coll	Tool	Recr	Rel1	Fit1	Fit2	Fit3	Ana	Rel2	User	Disc	Sum score (max)
	**Consensus**	**3**	**3**	**1**	**0**	**2**	**2**	**3**	**1**	**3**	**3**	**3**	**3**	**2**	**2**	**2**	**3**	**36 (48)**
Kayrouz (2015)[41]	R1	3	3	3	3	2	3	3	3	3	2	n	3	3	n	1	3	
	R2	3	3	3	2	1	3	2	3	2	2	n	3	2	n	2	3	
	**Consensus**	**3**	**3**	**3**	**3**	**1**	**3**	**3**	**3**	**2**	**2**	**n**	**3**	**3**	**n**	**2**	**3**	**37 (42)**
Kayrouz (2016a)[40]	R1	3	3	3	2	2	3	3	3	2	2	n	3	2	n	1	3	
	R2	3	3	3	3	1	3	2	3	3	2	n	3	1	n	1	3	
	**Consensus**	**3**	**3**	**3**	**3**	**1**	**3**	**2**	**3**	**3**	**2**	**n**	**3**	**2**	**n**	**1**	**3**	**35 (42)**
Kayrouz (2016b)[39]	R1	3	3	3	1	2	3	3	3	2	2	n	3	3	n	1	3	
	R2	3	3	3	3	2	3	2	3	2	2	n	3	1	n	1	3	
	**Consensus**	**3**	**3**	**3**	**3**	**2**	**3**	**3**	**3**	**2**	**2**	**n**	**3**	**2**	**n**	**1**	**3**	**36 (42)**
Knaevelsrud (2015)[42]	R1	3	3	3	0	3	2	3	3	0	2	n	2	1	n	0	3	
	R2	3	3	3	1	3	3	2	3	3	2	n	3	2	n	1	3	
	**Consensus**	**3**	**3**	**3**	**0**	**3**	**3**	**2**	**3**	**1**	**2**	**n**	**3**	**2**	**n**	**1**	**3**	**32 (42)**
Lal (2020)[46]	R1	3	3	3	0	2	3	1	3	n	n	3	2	1	2	2	1	
	R2	2	3	3	1	2	3	3	1	n	n	3	3	3	3	3	2	
	**Consensus**	**2**	**3**	**3**	**0**	**2**	**3**	**2**	**1**	**n**	**n**	**3**	**2**	**2**	**2**	**2**	**1**	**28 (42)**
Lin (2020)[16]	R1	3	3	3	3	1	1	2	3	2	3	n	3	2	n	1	1	
	R2	3	3	3	1	1	2	3	3	3	3	n	2	2	n	1	1	
	**Consensus**	**3**	**3**	**3**	**3**	**1**	**2**	**3**	**3**	**3**	**3**	**n**	**3**	**2**	**n**	**1**	**1**	**34 (42)**
Lindegaard (2020)[48]	R1	3	3	3	3	2	3	3	3	3	3	n	3	3	n	2	3	
	R2	3	3	2	3	3	3	2	3	3	3	n	3	3	n	0	2	
	**Consensus**	**3**	**3**	**2**	**3**	**3**	**3**	**3**	**3**	**3**	**3**	**n**	**3**	**3**	**n**	**2**	**3**	**40 (42)**
Luo (2020)[50]	R1	3	2	3	0	3	3	3	3	3	3	n	3	2	n	0	3	
	R2	3	3	3	1	3	2	3	3	3	3	n	2	2	n	1	2	
	**Consensus**	**3**	**2**	**3**	**0**	**3**	**3**	**3**	**3**	**3**	**3**	**n**	**3**	**2**	**n**	**1**	**3**	**35 (42)**
Muroff (2017)[52]	R1	3	2	2	0	1	2	1	2	1	1	n	2	2	n	1	2	
	R2	3	1	3	1	1	2	2	1	1	1	n	1	1	n	1	3	
	**Consensus**	**3**	**2**	**3**	**0**	**1**	**2**	**1**	**1**	**1**	**1**	**n**	**1**	**1**	**n**	**1**	**3**	**21 (42)**
Muroff (2019)[53]	R1	3	3	3	1	2	3	2	2	3	3	n	3	2	n	0	2	
	R2	3	3	3	0	2	3	3	2	3	2	n	2	2	n	1	2	
	**Consensus**	**3**	**3**	**3**	**1**	**2**	**3**	**3**	**2**	**3**	**3**	**n**	**2**	**2**	**n**	**0**	**2**	**32 (42)**

1st author (year)	Reviewer	Fram	Aim	Set	Sam	Repr	Coll	Tool	Recr	Rel1	Fit1	Fit2	Fit3	Ana	Rel2	User	Disc	Sum score (max)
Nygren (2018)[49]	R1	3	3	3	0	1	3	2	2	0	2	3	2	1	0	2	3	
	R2	3	3	3	1	1	3	3	2	1	1	3	2	1	3	1	3	**34**
	**Consensus**	**3**	**3**	**3**	**0**	**1**	**3**	**3**	**2**	**1**	**2**	**3**	**2**	**1**	**2**	**2**	**3**	**(48)**
Nygren (2019)[55]	R1	3	3	3	1	2	3	3	3	3	3	n	3	3	n	1	3	
	R2	3	3	3	3	1	3	3	3	3	3	n	3	2	n	1	3	**36**
	**Consensus**	**3**	**3**	**3**	**1**	**2**	**3**	**3**	**3**	**3**	**3**	**n**	**3**	**2**	**n**	**1**	**3**	**(42)**
Okujava (2019)[57]	R1	2	3	2	0	1	1	1	2	0	2	n	2	2	n	0	1	
	R2	2	3	3	0	1	1	2	3	0	1	n	1	1	n	0	1	**18**
	**Consensus**	**2**	**3**	**3**	**0**	**1**	**1**	**1**	**3**	**0**	**1**	**n**	**1**	**1**	**n**	**0**	**1**	**(42)**
Paris (2018)[59]	R1	2	3	2	1	2	2	1	2	0	3	n	3	2	n	1	2	
	R2	3	3	3	1	2	3	2	3	1	3	n	3	2	n	0	3	**30**
	**Consensus**	**2**	**3**	**3**	**1**	**2**	**3**	**2**	**3**	**0**	**3**	**n**	**3**	**2**	**n**	**0**	**3**	**(42)**
Patel (2017)[63]	R1	3	3	3	0	2	2	2	1	n	n	2	2	0	0	0	2	
	R2	3	3	3	1	1	3	3	3	n	n	3	3	2	3	2	2	**27**
	**Consensus**	**3**	**3**	**3**	**0**	**2**	**3**	**3**	**2**	**n**	**n**	**2**	**2**	**0**	**0**	**2**	**2**	**(42)**
Pinto-Bruno (2019)[64]	R1	3	3	3	3	n	3	1	n	n	3	n	3	3	n	0	1	
	R2	3	3	3	3	n	3	2	n	n	3	n	3	1	n	2	2	**27**
	**Consensus**	**3**	**3**	**3**	**3**	**n**	**3**	**1**	**n**	**n**	**3**	**n**	**3**	**2**	**n**	**1**	**2**	**(33)**
Rahmadiana (2019)[66]	R1	3	3	3	3	n	3	3	n	n	3	3	2	2	n	2	1	
	R2	3	3	3	3	n	2	3	n	n	3	3	3	3	n	2	3	**34**
	**Consensus**	**3**	**3**	**3**	**3**	**n**	**3**	**3**	**n**	**n**	**3**	**3**	**3**	**2**	**n**	**2**	**3**	**(36)**
Salamanca-Sanabria (2019)[67]	R1	3	2	3	0	1	3	3	3	2	2	2	3	2	2	2	3	
	R2	3	3	3	1	1	3	2	2	1	3	3	3	3	2	1	3	**36**
	**Consensus**	**3**	**2**	**3**	**0**	**1**	**3**	**2**	**3**	**2**	**3**	**3**	**3**	**2**	**2**	**1**	**3**	**(48)**
Salamanca-Sanabria (2020)[68]	R1	3	3	3	0	3	3	3	3	3	3	n	3	3	n	2	3	
	R2	3	3	3	1	3	3	3	2	3	2	n	3	3	n	2	3	**37**
	**Consensus**	**3**	**3**	**3**	**0**	**3**	**3**	**3**	**3**	**3**	**2**	**n**	**3**	**3**	**n**	**2**	**3**	**(42)**
Saulsberry (2013)[70]	R1	3	3	3	0	1	3	2	2	n	n	2	3	0	2	2	1	
	R2	3	3	3	1	2	3	3	1	n	n	3	3	2	3	3	1	**31**
	**Consensus**	**3**	**3**	**3**	**0**	**2**	**3**	**3**	**1**	**n**	**n**	**3**	**3**	**0**	**3**	**3**	**1**	**(42)**
Shala (2020)[71]	R1	3	3	3	0	1	2	3	1	n	n	3	3	2	3	2	2	
	R2	3	3	3	1	3	3	2	1	n	n	3	3	2	3	3	3	

1st author (year)	Reviewer	Fram	Aim	Set	Sam	Repr	Coll	Tool	Recr	Rel1	Fit1	Fit2	Fit3	Ana	Rel2	User	Disc	Sum score (max)
	**Consensus**	**3**	**3**	**3**	**0**	**3**	**2**	**3**	**1**	**n**	**n**	**3**	**3**	**2**	**3**	**2**	**2**	**33 (42)**
Silva (in prep)[60]	R1	3	3	2	n	1	2	2	1	1	3	n	2	2	n	3	2	
	R2	3	3	3	n	3	1	1	1	1	3	n	2	0	n	2	2	**29 (39)**
	**Consensus**	**3**	**3**	**3**	**n**	**2**	**2**	**2**	**1**	**1**	**3**	**n**	**2**	**2**	**n**	**3**	**2**	
Silva (2020)[61]	R1	2	2	3	1	2	2	2	2	1	1	n	1	1	n	1	1	
	R2	3	2	2	1	2	3	3	1	1	3	n	3	3	n	0	2	**26 (42)**
	**Consensus**	**3**	**2**	**3**	**1**	**2**	**3**	**2**	**1**	**1**	**3**	**n**	**2**	**2**	**n**	**0**	**1**	
Sit (2020)[72]	R1	3	2	3	0	2	2	2	3	n	n	3	2	1	0	1	3	
	R2	3	3	1	3	3	2	2	1	n	n	3	2	2	2	3	3	**30 (42)**
	**Consensus**	**3**	**2**	**3**	**1**	**3**	**2**	**2**	**2**	**n**	**n**	**3**	**2**	**0**	**2**	**2**	**3**	
Sobowale (2013)[3232]	R1	3	3	3	1	2	3	2	2	n	n	3	3	2	3	2	3	
	R2	3	3	3	1	1	3	2	1	n	n	3	3	2	3	2	3	**34 (42)**
	**Consensus**	**3**	**3**	**3**	**1**	**2**	**3**	**2**	**1**	**n**	**n**	**3**	**3**	**2**	**3**	**2**	**3**	
Spanhel (2019)[73]	R1	3	3	3	1	1	3	3	3	n	n	3	3	3	2	3	3	
	R2	3	3	3	3	1	3	2	2	n	n	2	3	3	2	2	3	**33 (42)**
	**Consensus**	**3**	**3**	**3**	**0**	**1**	**3**	**2**	**3**	**n**	**n**	**2**	**3**	**3**	**2**	**2**	**3**	
Teles (2020)[75]	R1	3	3	2	3	2	3	3	0	n	n	2	2	2	1	1	3	
	R2	3	3	3	3	1	2	1	1	n	n	2	2	1	1	1	3	**27 (42)**
	**Consensus**	**3**	**3**	**2**	**3**	**2**	**2**	**2**	**0**	**n**	**n**	**2**	**2**	**1**	**1**	**1**	**3**	
Titov (2018)[76]	R1	2	3	3	0	2	2	1	3	1	3	n	3	2	n	0	2	
	R2	1	3	3	1	2	3	3	2	1	3	n	3	2	n	1	3	**28 (42)**
	**Consensus**	**1**	**3**	**3**	**0**	**2**	**3**	**1**	**2**	**1**	**3**	**n**	**3**	**2**	**n**	**1**	**3**	
Ünlü Ince (2013)[77]	R1	3	3	3	3	2	3	3	3	3	3	n	3	2	n	0	3	
	R2	3	3	3	3	1	3	3	3	3	3	n	2	2	n	1	3	**35 (42)**
	**Consensus**	**3**	**3**	**3**	**3**	**1**	**3**	**3**	**3**	**3**	**3**	**n**	**2**	**2**	**n**	**0**	**3**	
Vöhringer (2020)[43]	R1	3	3	3	1	3	3	2	3	3	3	n	2	2	n	0	3	
	R2	2	3	3	0	3	2	3	1	3	3	n	3	3	n	1	3	**34 (42)**
	**Consensus**	**2**	**3**	**3**	**0**	**3**	**3**	**3**	**2**	**3**	**3**	**n**	**3**	**3**	**n**	**0**	**3**	
Wagner (2012)[44]	R1	2	3	3	0	1	2	3	2	2	2	n	2	2	n	0	3	
	R2	3	3	3	1	2	3	3	3	3	3	n	3	2	n	0	3	**30 (42)**
	**Consensus**	**3**	**3**	**3**	**0**	**1**	**3**	**3**	**2**	**3**	**2**	**n**	**2**	**2**	**n**	**0**	**3**	

1st author (year)	Reviewer	Fram	Aim	Set	Sam	Repr	Coll	Tool	Recr	Rel1	Fit1	Fit2	Fit3	Ana	Rel2	User	Disc	Sum score (max)
Wang (2013)[79]	R1	2	3	3	3	2	3	2	3	3	3	n	3	2	n	0	2	**37 (42)**
	R2	3	3	3	3	3	3	2	3	3	3	n	3	3	n	0	3	
	**Consensus**	**3**	**3**	**3**	**3**	**3**	**3**	**2**	**3**	**3**	**3**	**n**	**3**	**2**	**n**	**0**	**3**	
Wasil (2020)[81]	R1	3	2	2	0	1	2	2	2	n	n	2	2	2	0	2	0	**20 (42)**
	R2	2	3	3	1	2	2	2	1	n	n	3	0	0	0	2	1	
	**Consensus**	**3**	**2**	**2**	**0**	**1**	**2**	**2**	**1**	**n**	**n**	**2**	**2**	**1**	**0**	**2**	**0**	
Yokomitsu (2020)[83]	R1	3	3	2	3	n	3	3	n	n	3	n	2	1	n	0	1	**25 (33)**
	R2	2	3	3	3	n	3	3	n	n	3	n	3	2	n	0	2	
	**Consensus**	**2**	**3**	**3**	**3**	**n**	**3**	**3**	**n**	**n**	**3**	**n**	**3**	**1**	**n**	**0**	**1**	
**Mean of consensus ratings**		**2.82**	**2.87**	**2.82**	**1.20**	**1.88**	**2.71**	**2.35**	**2.06**	**1.88**	**2.63**	**2.67**	**2.60**	**1.82**	**2.04**	**1.27**	**2.49**	**31.36**

Fram Explicit theoretical framework, *Aim* Statement of aims/ objectives in main body of report, *Set* Clear description of research settings, *Sam* Evidence of sample size considered in terms of analysis, *Repr* Representative sample of target group of a reasonable size, *Coll* Description of procedure for data collection, *Tool* Rationale for choice of data collection tool(s), *Recr* Detailed recruitment data, *Rel1* Statistical assessment of reliability and validity of measurement tool(s) (quantitative), *Fit1* Fit between stated research question and method of data collection (quantitative), *Fit2* Fit between stated research question and format and content of data collection tool (qualitative), *Fit3* Fit between research question and method of analysis, *Ana* Good justification for analytical method selected, *Rel2* Assessment of reliability of analytical process (qualitative), *User* Evidence of user involvement in design, *Disc* Strengths and limitations critically discussed, *R1* first reviewer, *R2* second reviewer, *n* not applicable.

Supplementary Table 5: Detailed illustration of the conducted cultural adaptations of the 17 extracted aspects

1. Illustrated characters	Content/ background of characters	Appearances/ names of characters	Role of the characters
Abi Ramia (2018)[1]<br>Harper Shehadeh (2020)[2]	stories of the role models more relatable to the target group to enable identification	illustrated characters got more realistic faces; hand gesture of main characters changed	role models should not be prescriptive but provide the story of how they used instructions that the doctor gave to encourage the user to do the same thing
Abuwalla (2017)[4]	stories written by Arab adolescents so to fit the Arab context	names that fit the Arab context	
Arjadi (2018a+b)[6,7]	stories of previous participants’ experiences; role models more relatable to the Indonesian culture	case examples presented with male and female figures	
Bolinski (2018)[9]		culturally diverse characters that illustrate exercises and examples etc. (e.g. person of colour, Muslim)	
Brooks (2013)[11]			
Burchert (2018)[12]	stories and issues similar to the target group to enable identification with the content	choice of narrators with different clothing styles: e.g. women with and without hijab	
Campbell (2015)[13]<br>Chen (2019)[15]<br>Lin (2020)[16]		Native actors in videos	
Choi (2012)[18]		redrawing illustrations to reflect people with Asian features	
DaPonte (2018)[20]		names of vignettes changed	
Eylem (2020)[22]		cultural case examples	
Garabiles (2019)[24]	role models with relatable stories and personalities: common problems of oversea workers; neutral examples that fit heterogeneous educational background and occupations	appropriate appearances and illustrations of role models (e.g. warm facial expression and hand gestures, different hairstyles, signs of wealth, should look successful); appropriate Filipino names with positive meanings (e.g. John and Leona instead of Karim and Zeina)	main character: older and successful oversea-worker explaining intervention to participant instead of medical or mental health professional (normalising experience of problems); characters offer encouragement to users, e.g. illustrations with thumbs-up sign

1. Illustrated characters	Content/ background of characters	Appearances/ names of characters	Role of the characters
Gorman (2013)[25]	role models with relatable stories (personal story about having a child affected by alcohol use during pregnancy)	pictures of Native women, babies and children to enhance identification	addition of audio and video clips of personal stories (e.g. parents of children with foetal alcohol spectrum disorder expressing their points of view and experiences)
Hiratsuka (2019)[27]	testimonial videos featuring American Indian/ Alaska Native who have experienced trauma; include people with a history of trauma		
Imamura (2019)[29]	role models with relatable stories (major stressors of nurses, e.g. job overload)	change of characters leading through the intervention	
Ip (2016)[31]			
Sobowale (2013)[32]			
Juniar (2019)[33]	more relevant case examples of different Indonesian students	Indonesian professor in the greeting section; pictures of role models to fit the Indonesian context, e.g. clothing	
Kaal (2020)[35]	adaptation of the social profiles and stories: considered the ethnic background to allow the users to identify with the virtual companions of the program	a local (little-known) actor who guides through the intervention has been used; replaced pictures of an animated personal fictional companion with pictures of local ethnic-looking people who could be a neighbour); renamed persons by names common in local culture	
Kanuri (2020)[37]	adaptation of the vignettes to fit the context of Indian college students	videos were recorded with locals	
Kayrouz (2015/ 2016a+b)[39–41]	role models with relatable stories: male and female role models with problems and burdens similar to those of the target group	images of peoples with Arab features and names	
Knaevelsrud (2015)[42] Vöhringer (2020)[43]			
Wagner (2012)[44]			
Lal (2020)[46]			

1. Illustrated characters	Content/ background of characters	Appearances/ names of characters	Role of the characters
Lindegaard (2020)[48]<br>Nygren (2018)[49]	role models with relatable stories	role models that are appropriate for the target audience	
Luo (2020)[50]		change of names of characters to popular Chinese names (e.g. Lindsay to Xiaoli); pictures of women were changed to Asian women	
Muroff (2017/ 2019)[52,53]	stories from clients and staff to make them more relatable		
Nygren (2019)[55]	role models more relatable to Kurdish experiences		
Okujava (2019)[57]	patients' example stories were transformed according socio-economic and cultural environment of Georgia	illustrations of characters were changed reflect typical Georgians	
Paris (2018)[59]<br>Silva (in prep)[60]<br>Silva (2020)[61]	role models with Latino cultural values and concepts and living culturally relevant experiences	family from a Latin American country as main characters	change of the narrator characteristics: no lecturing expert but an abuela sitting in her living room
Patel (2016)[63]	role models with relatable stories written by Chinese adolescents based on their own experiences	images and videos showing native Chinese adolescents	
Pinto-Bruno (2019)[64]		change of names of the role models	
Rahmadiana (2019)[66]	role models are Indonesian students with relatable stories	all illustrated characters portray Indonesian feature, e.g. name, facial features	
Salamanca-Sanabria (2019/ 2020)[67,68]	role models with relatable stories, topics and scenarios/ stereotypes	include typical daily live aspects: e.g. male student with traditional bag, image of average Colombian girl hairstyle and clothes matching current styles	
Saulsberry (2013)[70]	role models with relatable stories, reflecting vulnerability and protective factors, common life experiences and common problems of minorities; e.g. including the cultural and family context	videos / pictures with African American and Latino narrators; use of typical names	

1. Illustrated characters	Content/ background of characters	Appearances/ names of characters	Role of the characters
Shala (2020)[71]	new narratives for the characters with causes and symptoms mentioned most frequently by the participants and with appropriate treatment goals so to make them feel understood and not feel alone		
Sit (2020)[72]	changed the storyline for Chinese students	changed the design of the main characters; professional characters were changed from medical doctor/ senior characters/ kind mother character to a cartoon character in casual wear and a young female person (i.e. peers); leading characters were adapted to match the target population's gender, age, and style of dress, with common Chinese names	
Spanhel (2019)[73]	role models that illustrate culturally diverse characters to represent heterogeneous target group with relatable stories and problems	role models with culturally diverse looks and names	
Teles (2020)[75]	adapt illustrated scenarios to be precise and understandable	personal names of characters used in scenarios were changed	
Titov (2018)[76]			
Ünlü Ince (2013)[77]	role models with similar problems as target population	clothing of role models	
Wang (2013)[79]			
Wasil (2020)[81]	characters were made more relatable with relevant and similar problems	characters had Indian names	testimonials were added to help the students see how the concept helped other students; use vignettes to engage and motivate students

**1. Illustrated characters**	**Content/ background of characters**	**Appearances/ names of characters**	**Role of the characters**
Yokomitsu (2020)[83]		change of the lead character's gender from male to female with a white national costume; more gentle and maternal expression (instead of Maori chief who gives a powerful impression); include the context when it comes to characters, e.g. face and eyes were slightly rounded	

**2. Illustrated activities**	**Daily life**	**Gender-specific behaviour**	**Religious/ traditional activities**	**Coping strategies**
Abi Ramia (2018)[1] Harper Shehadeh (2020)[2]	adapt daily activities (e.g. do not give an example of the woman going out for a walk alone; rather use simple indoor self-care activities: personal hygiene, or outdoor more complex activities: going out with friends, organising a family trip)	more gender specific activities in order to go with realistic local situation (reorganising or tidying the house for women, playing football or exercising for men)	praying	relaxing activities
Abuwalla (2017)[4]	adaptation of activities, e.g. note the interdiction to listen to modern music		reading holy books and praying	religious activities as relaxing activities (meditative and calming benefits)
Arjadi (2018a+b)[6,7]	involve culturally related activities (e.g. going to the market instead of walking your dog in the park)		include religious activities (e.g. pray, go to religious places)	
Bolinski (2018)[9]				
Brooks (2013)[11]				
Burchert (2018)[12]				
Campbell (2015)[13]	include relevant scenarios: e.g. native themes in scenes at home, conversations about Native art and music		praying	native spirituality as coping strategies: prayer, sweats, singing, drumming
Chen (2019)[15] Lin (2020)[16]	adapted illustrated daily activities			

2. Illustrated activities

	Daily life	Gender-specific behaviour	Religious/ traditional activities	Coping strategies
Choi (2012)[18]				use of traditional passive coping strategies, e.g. withdrawal and "accepting fate"
DaPonte (2018)[20]				
Eylem (2020)[22]				
Garabiles (2019)[24]	adapt jobs and tasks people do, use relatable activities; e.g. filling up a package to the Philippines, preparing to go to work instead of doing household chores	include strong traditional gender roles, e.g. isolation from others (males don't show emotional weakness)		introduce enjoyable and doable activities as coping strategies, e.g. eating meals together, connecting with families back home, listening to music and singing; adapt coping strategies, e.g. staying in bed all day seen as positive coping strategy
Gorman (2013)[25]				emphasise activities with family and friends who do not drink
Hiratsuka (2019)[27]	add or omit suggested activities that are common or uncommon in American Indian/ Alaska Native communities			
Imamura (2019)[29]				
Ip (2016)[31] Sobowale (2013)[32]	increase references to engagement in pleasant social activities; adjustments to match Hong Kong context: parenting style, e.g. hyper-criticism of children			
Juniar (2019)[33]	include activities more related to Indonesia in exercises, e.g. hang out with friends after class, have a coffee with friends		praying (instead of: e.g. walking with dog, having a cocktail)	
Kaal (2020)[35]				
Kanuri (2020)[37]				

2. Illustrated activities

	Daily life	Gender-specific behaviour	Religious/ traditional activities	Coping strategies
Kayrouz (2015/ 2016a+b)[39–41]	culturally appropriate activities: e.g. fishing with cousins, visiting family instead of friends	gender separation in daily life	including religious activities to reduce psychological symptoms: e.g. prayer, acts of service and charity	
Knaevelsrud (2015)[42] Vöhringer (2020)[43]				
Wagner (2012)[44]				
Lal (2020)[46]	include Canadian do's and don'ts of writing a resume and a cover letter			
Lindegaard (2020)[48]				
Nygren (2018)[49]				
Luo (2020)[50]				
Muroff (2017/ 2019)[52,53]				added culturally relevant resources
Nygren (2019)[55]	adapt activities: rather social activities than individual activities			
Okujava (2019)[57]				
Paris (2018)[59] Silva (in prep)[60]				
Silva (2020)[61]				
Patel (2016)[63]				
Pinto-Bruno (2019)[64]	adaptation of activities, timetables			
Rahmadiana (2019)[66]	changing for typical Indonesian activities: e.g. playing soccer/badminton/ jogging instead of Nordic walking; change parts related to alcohol (e.g. instead: drinking tea/coffee/fruit juice)			
Salamanca-Sanabria (2019/ 2020)[67,68]	changing examples for more relevant content, e.g. "failing an exam" instead of "losing a driver licence"			

**2. Illustrated activities**	**Daily life**	**Gender-specific behaviour**	**Religious/ traditional activities**	**Coping strategies**
Saulsberry (2013)[70]				supportive peer relationships, age-related developmental tasks (e.g. participation in /leadership of adolescent organizations/ sports / activities)
Shala (2020)[71]	adapt activities to the living conditions in the migration context, in order not to evoke a stronger longing for family members in the homeland; e.g. social activities with grandparents are often not possible			
Sit (2020)[72]	adapt scenarios according to Chinese students (e.g. go to a café instead of making a picnic); change experiences to fit Chinese students (e.g. give a gentle touch on shoulder instead of a hug)			
Spanhel (2019)[73]	changing for relevant everyday habits, activities, jobs: e.g. television turned on, playing on smartphone; less focus on time and structure, e.g. less emphasis on homework (e.g. sleeping diary), plans, calendars		include religious activities	strategies to deal with rumination, particularly imagination exercises
Teles (2020)[75]	include typical activities and cultural habits (e.g. the kind of breakfast that is prepared) and customs (food, leisure activities)		include traditions	
Titov (2018)[76]	themes etc. reflecting experiences of indigenous people			
Ünlü Ince (2013)[77]				
Wang (2013)[79]				

2. Illustrated activities

	Daily life	Gender-specific behaviour	Religious/ traditional activities	Coping strategies
Wasil (2020)[81]	included examples that were more relevant for Indian adolescents (e.g. mentioning cricket rather than baseball)			
Yokomitsu (2020)[83]				

3. Illustrated environment/ burdens

	Mental health	Countries, politics	Education	Burdens
Abi Ramia (2018)[1]<br>Harper Shehadeh (2020)[2]		various cultures and religions; complex colonial and migration history	many illiterate people	high politicised post-conflict environment; war and violence; financial problems; unemployment; marital problems; do not focus on children because of those who might have lost their children during the war
Abuwalla (2017)[4]		high prevalence in these societies for bullying, obesity, decreased exercise, academic pressure, political conflicts, poverty, economic crises, forced immigration		
Arjadi (2018a+b)[6,7]				
Bolinski (2018)[9]				dealing with problems representative for the target group (e.g. dealing with family members, partners, and friend)
Brooks (2013)[11]	high risk of substance use	adding information about Native populations in general		minority; stressful environments
Burchert (2018)[12]			high illiterate rates among young people due to war	
Campbell (2015)[13]				
Chen (2019)[15]<br>Lin (2020)[16]		replaced a story in an exercise with a story about Chinese history		
Choi (2012)[18]				

3. Illustrated environment/ burdens

	Mental health	Countries, politics	Education	Burdens
DaPonte (2018)[20]		statistics relevant to Canada instead of Australia		
Eylem (2020)[22]		rephrasing thinking patterns according to the cultural knowledge of Turkish populations		
Garabiles (2019)[24]		living for better socioeconomic status of family instead of individual aspirations: working abroad to contribute to family's expenses/ family's sole provider		use relatable and typical problems: leaving their family, having personal conflicts, having too much work, having no break / day off, feeling lonely/ stressed/ anxious (immigration)
Gorman (2013)[25]	partly high rate of alcohol consumption and alcohol dependence	include examples of common drink types e.g. alcoholic energy drink; examples with alcohol drinking behaviour in native communities to address perceived norms		high infant mortality rate
Hiratsuka (2019)[27]		some people may not have a quiet place		additional content on historical trauma; additional possible trauma triggers were included that fit the context
Imamura (2019)[29]				various stressors due to job setting: high workload, emotional cost of caring, lack of reward, shift work, leadership/ management style, professional conflict
Ip (2016)[31] Sobowale (2013)[32]	possible comorbid conditions of depression: alcohol and gambling endorsed in society	adjustments to match Hong Kong context		
Juniar (2019)[33]		place (e.g. landscape); exclude mention of traveling and getting souvenirs		students cope with: academic and social demands, career preparation after graduation; stress can lead to social isolation, lower academic performance, eventually study dropout
Kaal (2020)[35]				

3. Illustrated environment/ burdens

	Mental health	Countries, politics	Education	Burdens
Kanuri (2020)[37]				included stressors relevant to the target group
Kayrouz (2015/ 2016a+b)[39–41]			low socio-economic status, e.g. due to difficulty of recognition of previous expertise	war in home countries; challenges in adjusting the life in Australia (shame and failure)
Knaevelsrud (2015)[42] Vöhringer (2020)[43] Wagner (2012)[44]	low medical infrastructure due to war	frequent power cuts		situation of (civil) war and violence due to political situation increases traumatising events: e.g. human rights abuses, torture, killing, disappearances, beatings, experiencing death of family and friends
Lal (2020)[46]		make the contents locally relevant		
Lindegaard (2020)[48] Nygren (2018)[49]		eventually adding information about good habits when living in Sweden, e.g. eating vitamin D supplements, importance of going outside despite the cold weather		pre- and postmigration stressors; exposure to armed conflict and trauma in country of origin; uncertainty due to asylum-seeking process
Luo (2020)[50]		Chinese advertisements were included in educational games		
Muroff (2017/ 2019)[52,53]	stressors: higher rate of mental disorders, disease and poverty	links to relevant podcasts		acculturation process
Nygren (2019)[55]		including examples of Kurdish culture: e.g. food and drinking preferences; unsecure political situation in home country; dissatisfaction with Swedish society		loss of stressors related to pre- peri- and post-migration: e.g. persecution, violence, torture, uncertainty due to asylum seeking process, perception of discrimination, worry about political situation in home country, loneliness, family conflicts

**3. Illustrated environment/ burdens**				
	**Mental health**	**Countries, politics**	**Education**	**Burdens**
Okujava (2019)[57]		context was updated according to the Georgian reality		
Paris (2018)[59] Silva (in prep)[60] Silva (2020)[61]		poverty, unemployment, lack of health insurance		culturally relevant experiences: e.g. immigration-related family separation; vulnerability because of racism and discrimination, social exclusion, and physical comorbidities
Patel (2016)[63]				one-child families: high level of pressure for academic excellence as stressor for adolescents, lack of siblings could lead to decreased social skills, loneliness, lack of outlet for frustration, less support in conflicts with parents; stressors: absence of parents due to migration for work (from rural areas)
Pinto-Bruno (2019)[64]				
Rahmadiana (2019)[66]		incorporate the current living circumstances of the Indonesian population		face various changes: study commitments, financial shortage, living independently for the first time in live; demanding studies: high study workload
Salamanca-Sanabria (2019/ 2020)[67,68]	low access to mental health services, e.g. due to limited health insurance			e.g. pregnant student, economic problems, difficulties in romantic or peer relationships, high self-criticism/ perfectionism
Saulsberry (2013)[70]			low socio-economic status, low level of parents' education	stories should reflect the unique vulnerability and protective factors experienced by ethnic minority adolescents; chronic life stressors: e.g. exposure to neighbourhood violence; immigration stress, sexual activity, substance abuse, emotional trauma, physical trauma

**3. Illustrated environment/ burdens**				
	**Mental health**	**Countries, politics**	**Education**	**Burdens**
Shala (2020)[71]				included causes of problems that are frequently reported by the target group/ aspects related to migration (e.g. post-migration living difficulties, desire to return to the home country, the lack of wealth in retirement as a result of the social decline after migration, pressure to be good in job or education; discrimination; difficulties in cultural belonging)
Sit (2020)[72]		changed transportation (e.g. not use cab but bus): include a list of local services to meet the needs of young adults (mental healthcare; career development)		inclusion of problems relevant to Chinese students (e.g. free-riders in group projects)
Spanhel (2019)[73]	different reasons for sleeping problems (adapt to flight, being away from family, living situation); poor mental health	adapt specific recommendations/ rules to situation of target group: e.g. "prepare bed to signalise bedtime" rather than "preparing bedroom nicely" (common living situations without bedroom); adapt contents to the context (bed room, noise in a refugee camp,...)	low educational level and high level of illiteracy (no education during war)	pre-, peri- and postflight stressors: e.g. traumata, war and flight experiences; concern about security, worry for security of family; uncertainty concerning asylum-seeking process: e.g. illegal status, lack of stability; poor living, poor sleeping conditions; no pictures of the sea, avoidance of other links to the flight
Teles (2020)[75]		include national statistics and empirical studies on the Portuguese population (e.g. most popular leisure activities); national registries (e.g. names), and resource mapping (e.g. websites and informative contents)		
Titov (2018)[76]	poor mental health	family and community violence		intergenerational trauma; longing for country

3. Illustrated environment/ burdens

	Mental health	Countries, politics	Education	Burdens
Ünlü Ince (2013)[77]	high rates of depression among women			stressors connected to migration: complexities in adjustment process, unemployment, minority status, intergenerational tensions; e.g. young woman who migrated two years ago and can't find her way in the Netherlands
Wang (2013)[79]				
Wasil (2020)[81]		ensure concordance with local culture and context		
Yokomitsu (2020)[83]				

4. Illustrated values/ traditions

	Handling relationships/ sexuality / marriage	Value/ importance of family/ community	Value/ importance of religion/ respect/ spirituality	other values
Abi Ramia (2018)[1] Harper Shehadeh (2020)[2]	social support from friends and siblings rather than from spouse (Syrian women fear prejudice or abandonment; men fear showing weakness)		religion as important aspect in live and help against distress	
Abuwalla (2017)[4]	gender segregation e.g. no story about male-female relationships; males are dominant	importance of family unity (emotional support, support in treatment): family secrets are not shared outside the family; focus on community or tribal background; societal and family norms, values, roles, and authority directives predict behaviour	respect for elderly and man/ tradition/ marriage/ hospitality	educational achievement (pressure for academic success)/ stability/ modesty/ good income
Arjadi (2018a+b)[6,7]	male therapist character instead of female (patriarchal system)			
Bolinski (2018)[9]				

4. Illustrated values/ traditions

	Handling relationships/ sexuality / marriage	Value/ importance of family/ community	Value/ importance of religion/ respect/ spirituality	other values
Brooks (2013)[11]		patients were encouraged to talk with their family members about traumatisation	spirituality as important factor in community	
Burchert (2018)[12]				
Campbell (2015)[13]				
Chen (2019)[15]				included Chinese values and culture, customs and traditions to make it more acceptable
Lin (2020)[16]				
Choi (2012)[18]			promote cultural values of respect and interpersonal harmony instead of individual autonomy	
DaPonte (2018)[20]				
Eylem (2020)[22]		importance of family, e.g.: I have failed my family, I am a black spot in my family`s forehead	importance of honour	
Garabiles (2019)[24]		highlighting Filipino values: family orientation, hospitality, warmth, positive thinking, sociability: e.g. characters thinking of their families and missing them; social hierarchy and harmony in community are important		
Gorman (2013)[25]		family and community as important value: high influence on alcohol use behaviour, e.g. program designed for "you and other women in our community"		

4. Illustrated values/ traditions	Handling relationships/ sexuality / marriage	Value/ importance of family/ community	Value/ importance of religion/ respect/ spirituality	other values
Hiratsuka (2019)[27]		content on healthy relationships; focus on people in context of their community and families		replace all references to assertiveness with expressing oneself
Imamura (2019)[29]				
Ip (2016)[31]<br>Sobowale (2013)[32]		importance of families; collectivistic community, value of harmony within social relations		importance of academic success
Juniar (2019)[33]				
Kaal (2020)[35]				
Kanuri (2020)[37]				
Kayrouz (2015/ 2016a+b)[39–41]	exclusion of image depicting male and females in discussion	high importance of family: e.g. male example seeking advice from elder brother instead of friend; collectivistic culture	religion and respect are important	importance of status: loss of status associated with shame
Knaevelsrud (2015)[42]<br>Vöhringer (2020)[43]<br>Wagner (2012)[44]		respect of family is important: e.g. therapist expressed explicit respect toward family and concept of family, female participants with trauma of sexual abuse are explicitly discouraged from sharing their traumatic experience to other family members due to potential serious social consequences of known dishonour	religion is important and helpful	
Lal (2020)[46]				
Lindegaard (2020)[48]<br>Nygren (2018)[49]	eventually addressing issues related to shame and guilt concerning culture-specific practices, e.g. culture-specific sexual practices, child-bearing practices			
Luo (2020)[50]				

4. Illustrated values/ traditions

	Handling relationships/ sexuality / marriage	Value/ importance of family/ community	Value/ importance of religion/ respect/ spirituality	other values
Muroff (2017/ 2019)[52,53]		importance of family: include in recovery process, social isolation due to disconnections from family is especially challenging	integrate cultural beliefs and relevant sociocultural factors	
Nygren (2019)[55]		collectivistic community: focus on social activities, e.g. engaging with family and relatives); importance of family bonds and maintaining in-group harmony		
Okujava (2019)[57]				Georgian values, customs and traditions were taken into consideration
Paris (2018)[59] Silva (in prep)[60]	Include gender-specific values (e.g. machismo, caballerismo, marianismo)	family orientation, value of interpersonal relationships (personalismo)	Include respect, trust, wisdom	include fatalism
Silva (2020)[61]				
Patel (2016)[63]		high importance of family and collectivistic community: inclusion of family into intervention as a strong protective factor		themes more relevant to a Chinese adolescent, e.g. pressure for academic excellence, filial piety, and balancing school and social life
Pinto-Bruno (2019)[64]				
Rahmadiana (2019)[66]	changing/excluding parts related to unmarried cohabitation (e.g. instead: living with siblings/cousins), sexual activity, sexual terms (e.g. erection)		majority are Muslim students, inappropriate topics are excluded	

4. Illustrated values/ traditions	Handling relationships/ sexuality / marriage	Value/ importance of family/ community	Value/ importance of religion/ respect/ spirituality	other values
Salamanca-Sanabria (2019/ 2020)[67,68]				adaptations based on observable norms, values, and beliefs of the target population
Saulsberry (2013)[70]		stories featuring the cultural and family contexts: high connection to community, high importance of family: e.g. enhancing connectedness to family		
Shala (2020)[71]		include families and friends in the treatment goals (not only individual needs); take into account possible consequences in the family and community that might be more relevant for participants than their own individual suffering		
Sit (2020)[72]		include interactions with friends		included specific cultural knowledge related to local values, customs, and traditions within the Chinese background (e.g. don't verbalise compliments)
Spanhel (2019)[73]		importance of family (include in examples)	importance of religion (include in coping strategies)	
Teles (2020)[75]				include norms, values, and beliefs of the Portuguese population
Titov (2018)[76]		content on topics of family and community violence		longing for their country
Ünlü Ince (2013)[77]				

4. Illustrated values/ traditions

	Handling relationships/ sexuality / marriage	Value/ importance of family/ community	Value/ importance of religion/ respect/ spirituality	other values
Wang (2013)[79]				
Wasil (2020)[81]				include locally-valued character strengths: gratitude, growth, and positive activities
Yokomitsu (2020)[83]				

5. Language translation

	Translating intervention	Translating to dialect	Providing various language options to choose
Abi Ramia (2018)[1]	English to Arabic	spoken Arabic	
Harper Shehadeh (2020)[2]			
Abuwalla (2017)[4]	English to Arabic	most common dialects of Arabic	
Arjadi (2018a+b)[6,7]	Dutch to English to Bahasa Indonesia		
Bolinski (2018)[9]	German to Dutch		
Brooks (2013)[11]			
Burchert (2018)[12]	Arabic to different languages (Kurdish, formal Arabic, etc.)		offer more language options (supply native language support for all important services)
Campbell (2015)[13]			
Chen (2019)[15]	German to Chinese		
Lin (2020)[16]			
Choi (2012)[18]	English to Chinese		
DaPonte (2018)[20]	English to French		
Eylem (2020)[22]	Dutch to Turkish, English		
Garabiles (2019)[24]	English to Filipino language		
Gorman (2013)[25]			
Hiratsuka (2019)[27]			
Imamura (2019)[29]	Japanese to Vietnamese		
Ip (2016)[31]	English to Kantonese		
Sobowale (2013)[32]			

5. Language translation	Translating intervention	Translating to dialect	Providing various language options to choose
Juniar (2019)[33]	German to English to Bahasa Indonesia		
Kaal (2020)[35]	German to Estonian and Russian		Russian, Estonian
Kanuri (2020)[37]			
Kayrouz (2015/ 2016a+b)[39–41]	English to Arabic		
Knaevelsrud (2015)[42] Vöhringer (2020)[43]	Dutch to modern standard Arabic		
Wagner (2012)[44]			
Lal (2020)[46]		use audio tracks read by individuals with a Canadian accent	
Lindegaard (2020)[48]	Swedish to English to Arabic		
Nygren (2018)[49]			
Luo (2020)[50]	English to Chinese		
Muroff (2017/ 2019)[52,53]	English to Spanish		
Nygren (2019)[55]	Swedish to Kurdish		
Okujava (2019)[57]	Dutch to Georgian		
Paris (2018)[59] Silva (in prep)[60]	English to Spanish		
Silva (2020)[61]			
Patel (2016)[63]	English to Mandarin Chinese		
Pinto-Bruno (2019)[64]	English to Dutch		
Rahmadiana (2019)[66]	German to English to Bahasa Indonesia		
Salamanca-Sanabria (2019/ 2020)[67,68]	English to Spanish	Colombian accent	
Saulsberry (2013)[70]	English to Spanish		
Shala (2020)[71]	English to Albanian		potentially also German
Sit (2020)[72]	English to Chinese	additional simplified Chinese version for a Macao Chinese version	
Spanhel (2019)[73]	German to English; eventually translation into various (native) languages		Provided in English and German
Teles (2020)[75]	from English to Portuguese		

5. Language translation			
	**Translating intervention**	**Translating to dialect**	**Providing various language options to choose**
Titov (2018)[76]			
Ünlü Ince (2013)[77]	Dutch to Turkish		
Wang (2013)[79]	English to Chinese		
Wasil (2020)[81]			
Yokomitsu (2020)[83]	English to Japanese		

6. Language tailoring			
	**Simplify text: simplifying sentences and technical terms**	**Use of informal language/ slang and mixing languages for keywords**	**Use of milder descriptions for mental health concepts / culturally sensitive terms**
Abi Ramia (2018)[1]<br>Harper Shehadeh (2020)[2]	simplifying psychoeducational parts for illiterate participants	different wording translations to appeal better and depict distress and recovery	use no medicalising or clinical terms such as depression or mental illness, but rather words like distress; Arabic word of "psyche" instead of "mood"
Abuwalla (2017)[4]			
Arjadi (2018a+b)[6,7]	easier words to keep it simple		
Bolinski (2018)[9]		the polite German form (i.e. 'Sie') has been exchanged for the colloquial form (i.e. 'Du').	
Brooks (2013)[11]	less technical phrasing, modify wording for easier readability	less formal, eliminate jargon	
Burchert (2018)[12]			change single terms (e.g. patient, doctor, grief)
Campbell (2015)[13]		include Native words and slang	
Chen (2019)[15]<br>Lin (2020)[16]	adapted examples in the text		used culturally appropriate words
Choi (2012)[18]	modifying phrases, concepts, and descriptions		include appropriate terms, e.g. lowmood or "closed heart" instead of depression
DaPonte (2018)[20]			

**6. Language tailoring**			
	**Simplify text: simplifying sentences and technical terms**	**Use of informal language/ slang and mixing languages for keywords**	**Use of milder descriptions for mental health concepts / culturally sensitive terms**
Eylem (2020)[22]	reducing literacy, rephrasing sentences		Use of cultural expression of sadness and the cultural concept honour
Garabiles (2019)[24]	simplify texts and words: use simplest words: "friends" instead of "peers"; shorten long sentences	specify abstract terms: e.g. "helping hands" instead of "social support"; use mix of English and Filipino words ("Taglish") if adequate, e.g. "congratulations" and "homesick" in English; changing tone of texts to become more conversational and story-like to sound more engaging and realistic, adapt to slang, e.g. address patients as "countryman" and "sis" or "bro"	changing of certain descriptions so it sounds less grave and normative ("stressed at the moment" instead of "suffering at the moment")
Gorman (2013)[25]	changes of words and texts, e.g. "blood system" instead of "bloodstream" to enhance comprehension		
Hiratsuka (2019)[27]			some words were changed, e.g. bad dream instead of nightmare
Imamura (2019)[29]			
Ip (2016)[31] Sobowale (2013)[32]	simplify theoretical jargon and words		
Juniar (2019)[33]	simplifying text	include everyday language not to be too formal	changing words, e.g. stress and "thinking too much" (=Banyak pikiran/kepikiran) instead of depression
Kaal (2020)[35]		customizing sentence construction to match local language structure and style	replaced translation matches with a word that has a more appropriate meaning in the contexts of local values
Kanuri (2020)[37]	simplification of the language to suit a population for whom English was often a second language	all audio and video were recorded by locals to ensure the accent and pronunciation were easy to understand	

**6. Language tailoring**	**Simplify text: simplifying sentences and technical terms**	**Use of informal language/ slang and mixing languages for keywords**	**Use of milder descriptions for mental health concepts / culturally sensitive terms**
Kayrouz (2015/ 2016a+b)[39–41]		use of "Salam Al laykum" as common greeting	use terms that are congruent and sensitive to values and meanings of the culture, e.g. exclusion of "psychological health" in the title of the course
Knaevelsrud (2015)[42] Vöhringer (2020)[43]			
Wagner (2012)[44]			
Lal (2020)[46]		include French words on a list indicating that the user is in risk, because local youths might be bilingual with their mother tongue being French	
Lindegaard (2020)[48] Nygren (2018)[49]	change semantics, simplify some words and explanation parts if too academic	change words primarily used in North African countries (confusing for other origins)	
Luo (2020)[50]		adaptation of words, e.g. in educational games	
Muroff (2017/ 2019)[52,53]		change words and terms relevant in several Central and South American countries	
Nygren (2019)[55]			
Okujava (2019)[57]		adjustment of language to fit Georgian reality	
Paris (2018)[59] Silva (in prep)[60] Silva (2020)[61]	changes in order to address a rather poor academic experience, e.g. use the word "practice" instead of "homework"	minimised accent and avoidance of colloquial or regional expressions to reduce the possibility that viewers would associate characters with one particular country	
Patel (2016)[63]		use of modern vernacular instead of traditional Chinese Idioms	
Pinto-Bruno (2019)[64]			

6. Language tailoring	Simplify text: simplifying sentences and technical terms	Use of informal language/ slang and mixing languages for keywords	Use of milder descriptions for mental health concepts / culturally sensitive terms
Rahmadiana (2019)[66]		if translated Filipino words/terms seem too extensive, the English word is also joined, e.g. suasana hati tertekan (Depressed mood)	
Salamanca-Sanabria (2019/ 2020)[67,68]		use of informal language style, e.g. "Tuteo": tú, more "colloquial use"; using questions and more informal expressions, e.g. "how to start to motivate myself?" instead of "boosting behaviour"	make it more understandable for the target population (e.g. I can feel better instead of Space from depression)
Saulsberry (2013)[70]		use of typical names, idioms, vernacular for youth	
Shala (2020)[71]			find an appropriate word for distress which is accepted by Albanian people from various regions
Sit (2020)[72]			change of expressions to fit the Chinese context
Spanhel (2019)[73]	simplify, concretise, clarify text (low level of abstraction/ reading); non-academic		
Teles (2020)[75]	changing formulation to find semantic and conceptual equivalences (e.g. use session instead of lesson) and to fit the reading-level of the target group (use plain words and avoid complex formulations; avoid long sentences)	use of jargon, colloquialisms; use an informal and straightforward verbal style	adapt any potentially stigmatising expression or term
Titov (2018)[76]			
Ünlü Ince (2013)[77]			describe psychological problems in terms of idioms of distress, e.g. using symptoms of depression instead of the term depression
Wang (2013)[79]			
Wasil (2020)[81]	simplified the wording		
Yokomitsu (2020)[83]			not speaking of homework but of work presentation

**7. Visualisation of language**	**Use of metaphors**	**Use of verbal expressions (sayings, quotes)**	**Use of symbols**
Abi Ramia (2018)[1]<br>Harper Shehadeh (2020)[2]	metaphors that appeal better and depict distress and recovery		
Abuwalla (2017)[4]		include verses of the Quran that refer to religious figures who struggled with the same doubts to promote seeking help	religious-neutral cultural symbols e.g. Dallah, dhow, palm trees, camels, sand dunes to increase identification
Arjadi (2018a+b)[6,7]			
Bolinski (2018)[9]		sayings have been adapted throughout the intervention	
Brooks (2013)[11]			
Burchert (2018)[12]			
Campbell (2015)[13]	include humour		
Chen (2019)[15]<br>Lin (2020)[16]			sayings were adapted to the Chinese culture
Choi (2012)[18]			
DaPonte (2018)[20]			
Eylem (2020)[22]	Include well-known idioms and metaphors describing psychological distress and suicide (e.g. in the name of the intervention: ‘Kiyma Canina-Don`t crush your life energy’ instead of ’suicide’)		
Garabiles (2019)[24]			shared concept: "utang-na-loob" (depth of gratitude), e.g. character reaches out to a friend who helped them before; "bayanihan" (working together to help someone)
Gorman (2013)[25]			
Hiratsuka (2019)[27]			
Imamura (2019)[29]			

7. Visualisation of language	Use of metaphors	Use of verbal expressions (sayings, quotes)	Use of symbols
Ip (2016)[31]			
Sobowale (2013)[32]			
Juniar (2019)[33]	changing metaphors: e.g. courier of valuables instead of post man		
Kaal (2020)[35]			the local name selected for the program - Selge - has two meanings: clear and sober
Kanuri (2020)[37]			
Kayrouz (2015/ 2016a+b)[39–41]			
Knaevelsrud (2015)[42]<br>Vöhringer (2020)[43]		include quotes and metaphors from the Koran to emphasise change of perspective and challenge dysfunctional thoughts	
Wagner (2012)[44]			
Lal (2020)[46]			
Lindegaard (2020)[48]			
Nygren (2018)[49]			
Luo (2020)[50]			
Muroff (2017/ 2019)[52,53]		culturally relevant sayings, e.g. Daily Thoughts, and inspirational quotes	
Nygren (2019)[55]			
Okujava (2019)[57]		sayings were adapted to Georgian realities in order to make them more familiar to the Georgian patients	symbols were used to facilitate the contents
Paris (2018)[59]<br>Silva (in prep)[60]		dichos (folk sayings or proverbs) to capture main message of each module	
Silva (2020)[61]			
Patel (2016)[63]			
Pinto-Bruno (2019)[64]			
Rahmadiana (2019)[66]			

**7. Visualisation of language**			
	**Use of metaphors**	**Use of verbal expressions (sayings, quotes)**	**Use of symbols**
Salamanca-Sanabria (2019/ 2020)[67,68]		add typical Colombian expressions in texts, e.g. "guayabo" (hung over), "ciclovía" (bike rides on Sundays); add quotes of Latin-American writers	
Saulsberry (2013)[70]			
Shala (2020)[71]	include culturally specific metaphors, proverbs, stories, analogies, and idioms (e.g. to address distress); translate metaphorical expressions by adopting a suitable equivalent		
Sit (2020)[72]	added metaphors as suggested by participants and used plain expressions	use local idioms/ phrases	
Spanhel (2019)[73]		exclude ambiguous terms, metaphors, sayings, puns	
Teles (2020)[75]		use of idioms or vernacular terms	
Titov (2018)[76]			
Ünlü Ince (2013)[77]			
Wang (2013)[79]			
Wasil (2020)[81]			
Yokomitsu (2020)[83]			

**8. Concepts of mental health (treatment)**					
	**Related to religion/ supernatural powers/ spirituality**	**Poor knowledge**	**Stigmatisation of mental health problems**	**Handling of mental health problems (traditional healing, treatment, expression of symptoms)**	**Distrust in treatment/ confidentiality**
Abi Ramia (2018)[1] Harper Shehadeh (2020)[2]	religion could be added as a solution to help against distress	lack of awareness of seriousness of mental distress /of benefits of interventions; reduce	high stigma related to depression	depressed people maintain necessary activities and still go working while becoming irritable and withdraw from social	Privacy, e.g. fear of spies; Syrian women share phone with husband (privacy is a barrier); credible

8. Concepts of mental health (treatment)	Related to religion/ supernatural powers/ spirituality	Poor knowledge	Stigmatisation of mental health problems	Handling of mental health problems (traditional healing, treatment, expression of symptoms)	Distrust in treatment/ confidentiality
		elements in interventions that encourage misconceptions, e.g. hot coffee or tea as treatment for depression		situations/ decrease of enjoyable activities; caused by external factors, e.g. wife’s behaviour; doctor is prescriptive and direct in advice and suggestions and patient should follow doctors’ advice	source of information to add credibility
Abuwalla (2017)[4]	focusing on the hereafter; mental illness as a result of the supernatural powers of demons/ religious conflicts, esp. aggressive behaviour; traditional healer can help get rid of the demon; include religion and spirituality in therapy: relying on Allah, thus include belief that medicine is a tool provided by God		high stigma of depression and treatment of depression	include Arab concepts of depression and acknowledge the understanding of depression (e.g. somatic symptoms); try to create a new, positive understanding	promote privacy/ anonymity of online therapy; use scientific evidence in order to accelerate recovery and enhance prevention; no costs, availability of internet
Arjadi (2018a+b)[6,7]		generally poor knowledge about depression	being depressed is taboo and stigmatised → present diverse figures to show that depression can happen to anyone		
Bolinski (2018)[9]					

8. Concepts of mental health (treatment)					
	**Related to religion/ supernatural powers/ spirituality**	**Poor knowledge**	**Stigmatisation of mental health problems**	**Handling of mental health problems (traditional healing, treatment, expression of symptoms)**	**Distrust in treatment/ confidentiality**
Brooks (2013)[11]	traditional healing as important factor: spirituality dialogue and assessments			more focus on behavioural assessments	high-level feedback on individual symptoms
Burchert (2018)[12]		low mental health literacy	lack of acceptance for mental health problems and treatment via online-interventions	consider differing health beliefs	increase trust in the app: lessen worries about data protection: explain privacy settings, use passwords; reduce doubts whether the program would actually help
Campbell (2015)[13]	include native spirituality as coping strategies	low mental health literacy	high stigma related to substance abuse and sexual behaviour	discomfort in a "westernised" treatment delivery system	distrust to education and information delivered by "Westerns"
Chen (2019)[15]					
Lin (2020)[16]					
Choi (2012)[18]		lack of knowledge about mental disorders and treatment	depression comes with high stigma and "loss of face"	somatisation of symptoms	
DaPonte (2018)[20]					
Eylem (2020)[22]	Use of religiosity		shame, especially related humiliating the family due to mental problems	information and exercises about self-harm have been removed	
Garabiles (2019)[24]			high stigma of mental health problems		

**8. Concepts of mental health (treatment)**					
	**Related to religion/ supernatural powers/ spirituality**	**Poor knowledge**	**Stigmatisation of mental health problems**	**Handling of mental health problems (traditional healing, treatment, expression of symptoms)**	**Distrust in treatment/ confidentiality**
Gorman (2013)[25]		high use of alcohol in general community and among pregnant women is partly seen as normal → reduce misconceptions			distrust and scepticism towards research; emphasise confidentiality of all data and independence of medical care; offer intervention in specific space within clinic
Hiratsuka (2019)[27]			focus on false beliefs and shame messages common among American Indian/ Alaska Native people who have experienced trauma		
Imamura (2019)[29]					
Ip (2016)[31]		rising risk of substance use and gambling: include questions about frequency and amount used	stigma associated with mental health issues: offer intervention in a non-stigmatising way		high distrust in treatment methods of arrival country and in mobile health interventions; emphasise consistent use
Sobowale (2013)[32]					
Juniar (2019)[33]			high stigma for mental health issues and for seeking mental health treatment	include biological symptoms of distress, e.g. easily irritated, feeling low, headache, loss of appetite	

**8. Concepts of mental health (treatment)**					
	**Related to religion/ supernatural powers/ spirituality**	**Poor knowledge**	**Stigmatisation of mental health problems**	**Handling of mental health problems (traditional healing, treatment, expression of symptoms)**	**Distrust in treatment/ confidentiality**
Kaal (2020)[35]					
Kanuri (2020)[37]					
Kayrouz (2015/ 2016a+b)[39–41]	high importance of religion in mental health and treatment: include monotheistic definition of spiritual health, e.g. trusting relationship with God	low mental health literacy	mental health problems as sign of weakness for individual and family (jeopar-dises their standing in community); shame associated with seeking mental health treatment		
Knaevelsrud (2015)[42] Vöhringer (2020)[43] Wagner (2012)[44]			many traumata are seen as weakness, are highly stigmatised and can lead to rejection by family → shame	health care professional as authoritative and highly respected figure: more directive therapeutic instructions	emphasise high standards of privacy and anonymity
Lal (2020)[46]					
Lindegaard (2020)[48] Nygren (2018)[49]			high stigma associated with mental health issues; normalise psychological problems in context of having re-cently gone through stressful changes and upheavals and help seeking	more emphasis on biological explanatory models; address concentration problems	increase anonymity

8. Concepts of mental health (treatment)

	Related to religion/ supernatural powers/ spirituality	Poor knowledge	Stigmatisation of mental health problems	Handling of mental health problems (traditional healing, treatment, expression of symptoms)	Distrust in treatment/ confidentiality
Luo (2020)[50]					
Muroff (2017/ 2019)[52,53]			high stigma associated to mental health problems and substance abuse		
Nygren (2019)[55]		unfamiliarity with western psychological concepts or treatment models	high stigma associated to seeking professional help and to mental health issues	common use of informal healers, e.g. family, friends; emphasis more on behavioural than cognitive interventions	fear of dependency on medication
Okujava (2019)[57]					
Paris (2018)[59]		low literacy in mental health		more of a mutual relationship between narrator and participant as hybrid of authority and facilitator	
Silva (in prep)[60]					
Silva (2020)[61]					
Patel (2016)[63]					
Pinto-Bruno (2019)[64]					
Rahmadiana (2019)[66]				changing/excluding parts related to drugs, anti-depressants, sleep medication, inpatient care	
Salamanca-Sanabria (2019/ 2020)[67,68]			stigma associated to mental health issues	expression of depression via somatic symptoms rather than using cognitive factors, e.g. pain, digestive problems	

8. Concepts of mental health (treatment)					
	**Related to religion/ supernatural powers/ spirituality**	**Poor knowledge**	**Stigmatisation of mental health problems**	**Handling of mental health problems (traditional healing, treatment, expression of symptoms)**	**Distrust in treatment/ confidentiality**
Saulsberry (2013)[70]			stigma associated with mental health problems and help seeking, e.g. psychiatric illness is perceived as social construct rather than medical disorder		historical mistrust in medical profession
Shala (2020)[71]	fatalistic assumption that suffering is given by fate or by God and has to be endured (external control attributions hindered help-seeking and treatment motivation)	participants did not differentiate between causes and symptoms	address stigmata by including persons who are going through similar experiences	include the concepts of distress and mental health: refer to fate and endurance; suffering concerns the interdependent self, not only the individual	experiences with the intervention can be shared with another person of trust; password-protected access to the intervention
Sit (2020)[72]			framing the goal of the intervention as a stress management tool or academic performance tool instead of mental health intervention in order to reduce the stigma	changed symptoms of not feeling well to fit the Chinese student's experience (e.g. dizziness instead of headache: feeble limbs instead of feeling tired)	

8. Concepts of mental health (treatment)

	Related to religion/ supernatural powers/ spirituality	Poor knowledge	Stigmatisation of mental health problems	Handling of mental health problems (traditional healing, treatment, expression of symptoms)	Distrust in treatment/ confidentiality
Spanhel (2019)[73]	high influence of religion and spirituality/ body and soul are closely related; inclusion of spiritual elements/appreciation of traditional concepts of mental disorders and explanation of western treatment	low mental health literacy and low experience with (mental) health professionals	high stigma or taboo associated with mental health issues and help seeking: e.g. low acceptance of mental disorders and their treatment	diverging concepts of disease and treatment among refugees; low expectation of self-efficacy and high rate of medication	emphasise privacy and data security issues
Teles (2020)[75]					
Titov (2018)[76]					
Ünlü Ince (2013)[77]			high stigma associated with mental health issues and seeking professional help		
Wang (2013)[79]					
Wasil (2020)[81]					
Yokomitsu (2020)[83]					

9. Goals of treatment

	Increase understanding/ acceptance of mental disorders	Increase understanding on treatment possibilities	Enhance coping strategies	other goals
Abi Ramia (2018)[1]			focus on relaxing and enjoyable activities and on gaining social support and resting without feeling guilty	
Harper Shehadeh (2020)[2]				

9. Goals of treatment

	Increase understanding/ acceptance of mental disorders	Increase understanding on treatment possibilities	Enhance coping strategies	other goals
Abuwalla (2017)[4]	encourage recognition of symptoms of depression and expression of psychiatric symptoms; reduce stigma about mental health		promote healthy relationships: focus on family wellbeing, e.g. restore the family order	focus on boosting academic performance
Arjadi (2018a+b)[6,7]			support good integration into the community by being more active	
Bolinski (2018)[9]				
Brooks (2013)[11]		informing on traumatisation and its management in stressful environments		
Burchert (2018)[12]				increase motivation to do the training
Campbell (2015)[13]				
Chen (2019)[15]				
Lin (2020)[16]				
Choi (2012)[18]	addressing myths about depression and its treatment			
DaPonte (2018)[20]				
Eylem (2020)[22]	Respecting users` possible concern about sharing their feelings with someone by encouraging them to imagine themselves sharing their feelings with someone in “an experiment in sharing your feelings”			change the goal of an exercise to be more specific ("Is my life worth living" instead of “Does life have any meaning?”)

9. Goals of treatment

	Increase understanding/ acceptance of mental disorders	Increase understanding on treatment possibilities	Enhance coping strategies	other goals
Garabiles (2019)[24]				less focus on mental health, more focus on positive goals & outcomes, focus on common & integral workers experience
Gorman (2013)[25]	enhance intrinsic motivation to reduce alcohol consumption in general and to avoid alcohol during pregnancy for the health of a future baby; resolving myths and misconceptions that alcohol use is widespread among you women			discuss the importance of addressing the problems also in regard to the family or friends
Hiratsuka (2019)[27]	additional content on acceptance and sharing of one's story			
Imamura (2019)[29]				
Ip (2016)[31] Sobowale (2013)[32]				goal of building resiliency and being a well-balanced, successful adult (monetarily, socially, academically)
Juniar (2019)[33]				
Kaal (2020)[35]				
Kanuri (2020)[37]			facilitate skills rehearsal and acquisition	
Kayrouz (2015/ 2016a+b)[39–41]		differentiating between unhelpful and helpful religious thoughts		handling the actual living situation
Knaevelsrud (2015)[42] Vöhringer (2020)[43]		possibility to share experiences without aversive consequences		
Wagner (2012)[44]				

9. Goals of treatment

	Increase understanding/ acceptance of mental disorders	Increase understanding on treatment possibilities	Enhance coping strategies	other goals
Lal (2020)[46]				
Lindegaard (2020)[48]	dispel common myths and misunderstandings about mental health			
Nygren (2018)[49]				
Luo (2020)[50]				
Muroff (2017/ 2019)[52,53]	inclusion of content addressing discrimination, stigma, acculturation			
Nygren (2019)[55]				
Okujava (2019)[57]				
Paris (2018)[59]				attend to social and environmental factors that increase vulnerability to substance use (limited social support, experiences of discrimination, unmet basic needs)
Silva (in prep)[60]				
Silva (2020)[61]				
Patel (2016)[63]	increase awareness of depression in China and will to deal with it			
Pinto-Bruno (2019)[64]				
Rahmadiana (2019)[66]				
Salamanca-Sanabria (2019/ 2020)[67,68]	psychoeducation and reduction of stigma			
Saulsberry (2013)[70]	concept of building resiliency rather than focus on illness; decrease stigma associated with depression		improvement of parent-adolescent communication to enhance protective factor of family	

9. Goals of treatment

	Increase understanding/ acceptance of mental disorders	Increase understanding on treatment possibilities	Enhance coping strategies	other goals
Shala (2020)[71]	explanatory model builder: provide a convincing treatment rationale to help to disentangle causes from symptoms and better understand how behaviour and mood are connected	convey that it is not a bad thing to seek help: there is no shame in seeking professional help → enhance treatment motivation		
Sit (2020)[72]				
Spanhel (2019)[73]	psychoeducational elements on various mental disorders	enhance self-efficacy and active approach of problems: e.g. including information on adequate handling and quitting of medication; information on the healthcare system and treatment possibilities	increase part for imagination or mindfulness exercises (also to address rumination), addressing resources	
Teles (2020)[75]				
Titov (2018)[76]	understanding how experiences of Indigenous people affect their mental health			
Ünlü Ince (2013)[77]				
Wang (2013)[79]				
Wasil (2020)[81]				
Yokomitsu (2020)[83]				

10. Methods of treatment

	Comprehensive psychoeducation	Information/ links to other helpful addresses	Emphasis on positive outcomes/ recovery	Other treatment methods
Abi Ramia (2018)[1]			More emphasis on solutions	
Harper Shehadeh (2020)[2]				

10. Methods of treatment

	Comprehensive psychoeducation	Information/ links to other helpful addresses	Emphasis on positive outcomes/ recovery	Other treatment methods
Abuwalla (2017)[4]			recall the purpose and effects of distress and afflictions	
Arjadi (2018a+b)[6,7]	include psychoeducation on depression to decrease stigma			
Bolinski (2018)[9]				
Brooks (2013)[11]	psychoeducation on traumatisation in American Indian population	add contact information for health and resources in the local area		
Burchert (2018)[12]		include links to external sources of information and support such as websites and to provide additional information regarding local health systems; implement a referral system to ensure that severe cases see a therapist		
Campbell (2015)[13]				skills for substance use
Chen (2019)[15]				
Lin (2020)[16]				
Choi (2012)[18]				
DaPonte (2018)[20]				
Eylem (2020)[22]	psychoeducation: explaining theoretical background			inclusion of the mindfulness exercise "Safe place"; more mentalisation techniques
Garabiles (2019)[24]			offer encourage-ment; increase emphasis on positive goals & outcomes (e.g. to have a good sleep instead of coping with sleep problems)	

10. Methods of treatment	Comprehensive psychoeducation	Information/ links to other helpful addresses	Emphasis on positive outcomes/ recovery	Other treatment methods
Gorman (2013)[25]	psychoeducation about risk factors for alcohol independence and foetal alcohol spectrum disorder and about importance of healthy lifestyle choices			
Hiratsuka (2019)[27]	include educational videos by members of the American Indian/ Alaska Native community; emphasise the impact of trauma on overall health			
Imamura (2019)[29]				include one module on self-compassion instead of physical activity for stress management
Ip (2016)[31] Sobowale (2013)[32]			include testimonials of successful people to motivate adherence	exclude interpersonal psychotherapy modules and motivational interview
Juniar (2019)[33]				
Kaal (2020)[35]		added a link to the program to the alcohol unit calculator		
Kanuri (2020)[37]				
Kayrouz (2015/ 2016a+b)[39–41]				
Knaevelsrud (2015)[42] Vöhringer (2020)[43] Wagner (2012)[44]				describe traumatic events without mentioning specific places or names of persons

10. Methods of treatment	Comprehensive psychoeducation	Information/ links to other helpful addresses	Emphasis on positive outcomes/ recovery	Other treatment methods
Lal (2020)[46]	add more specific and detailed information; add contents to physical health (e.g. sleep hygiene), mediation use and side effects	change contents of health service links to fit the local context (e.g.: need help now page; local job-seeking sites; government agencies; employment centres)	include stories about successful recovery	
Lindegaard (2020)[48]				use a transdiagnostic approach rather than a disorder-specific program
Nygren (2018)[49]				
Luo (2020)[50]				set up of an online community forum
Muroff (2017/ 2019)[52,53]	add educational recovery and trauma-informed materials		add recovery stories and resource articles	
Nygren (2019)[55]	explain behavioural model of depression with an example they can identify with; more video presentations due to unfamiliarity with Western psychological concepts or treatment models			
Okujava (2019)[57]	some medical information about sleep was updated according to recent data			
Paris (2018)[59]				
Silva (in prep)[60]				
Silva (2020)[61]				
Patel (2016)[63]				
Pinto-Bruno (2019)[64]				
Rahmadiana (2019)[66]		counselling services within the university vicinity instead of partial hospital programs and inpatient programs		

10. Methods of treatment	Comprehensive psychoeducation	Information/ links to other helpful addresses	Emphasis on positive outcomes/ recovery	Other treatment methods
Salamanca-Sanabria (2019/ 2020)[67,68]	include psychoeducation; increasing conceptual clarity between different parts of the programme		emphasise improvement of mood	
Saulsberry (2013)[70]				
Shala (2020)[71]	increase acceptance for meditation to reduce stress with an appropriate description and rationale; disentangle symptoms and causes by psychoeducation (address fatalistic beliefs); explain how behaviour and mood are linked		explain that the programme has helped many people	more personal and welcoming
Sit (2020)[72]		include a list of local services (mental healthcare; career development)	show that the leading characters feel better after doing the programme	changed the positive feedback for activities to emphasise their strength
Spanhel (2019)[73]	explanation of western treatment to increase understanding and legitimation; explanations of links of sleeping problems and other mental disorders	provide links to other places to find help	focus on peoples' resources and goals to increase positive expectancy (have overcome their sleeping problems)	increase part for imagination or mindfulness exercises
Teles (2020)[75]		adapt resources (information materials, services available, technologies, e.g. links to webpages or informative manuals)		
Titov (2018)[76]				
Ünlü Ince (2013)[77]				
Wang (2013)[79]				
Wasil (2020)[81]	more information on specific research on concepts; include information on the benefits of each concept on physical and emotional health			more concrete tasks in writing exercises

**10. Methods of treatment**				
	**Comprehensive psychoeducation**	**Information/ links to other helpful addresses**	**Emphasis on positive outcomes/ recovery**	**Other treatment methods**
Yokomitsu (2020)[83]	the user can obtain information related to mental health once a week			

**11. Structure**					
	**Shorten intervention**	**Changes in texts**	**More/ less repetitions**	**Simplify introduction**	**Add optional intervention elements**
Abi Ramia (2018)[1]<br>Harper Shehadeh (2020)[2]	total intervention is ca. 30% shorter (each module); more structured and organized sessions; longer time between sessions	less text and story as a video to watch	less repetitions		
Abuwalla (2017)[4]		shortened text			
Arjadi (2018a+b)[6,7]		shortened text		clear and precise instructions	
Bolinski (2018)[9]	considerably shortened the sessions (on average 10%, max. 40%)	shortened text			
Brooks (2013)[11]	shorten intervention, shorten educational components (1 year to 90 days)	shortened text	eliminate trivial questions and repeating content (redundancy)		add optional substance use assessments; including traditional and spiritual healing as optional content; add ability to individually select components and possibility to repeat certain contents
Burchert (2018)[12]	shorter but more frequent sessions	shortened text	less repetitions of information		
Campbell (2015)[13]<br>Chen (2019)[15]<br>Lin (2020)[16]		shortened text			

11. Structure	Shorten intervention	Changes in texts	More/ less repetitions	Simplify introduction	Add optional intervention elements
Choi (2012)[18]					
DaPonte (2018)[20]					
Eylem (2020)[22]		shortened text			
Garabiles (2019)[24]		shortened text			
Gorman (2013)[25]		shortened text			
Hiratsuka (2019)[27]					patient education on the website and communication of weekly tips via text messaging
Imamura (2019)[29]					fixed-order versus free-choice programme
Ip (2016)[31] Sobowale (2013)[32]	shorten content by 50%	shortened text	split stories to keep interest; less repetitions		
Juniar (2019)[33]	shorten intervention (6 instead of 7 modules)	shortened text			
Kaal (2020)[35]					added 2 mental health modules from depression prevention programme
Kanuri (2020)[37]	content was restructured to 20 (instead of 40) short sessions		techniques like breathing exercises and mindfulness were repeated		
Kayrouz (2015/ 2016a+b)[39–41]					
Knaevelsrud (2015)[42]					
Vöhringer (2020)[43]					
Wagner (2012)[44]					

**11. Structure**	**Shorten intervention**	**Changes in texts**	**More/ less repetitions**	**Simplify introduction**	**Add optional intervention elements**
Lal (2020)[46]		texts with more information			
Lindegaard (2020)[48]<br>Nygren (2018)[49]	shorten intervention	shorter versions of material		introductory video clip to introduce the web page	
Luo (2020)[50]					
Muroff (2017/ 2019)[52,53]		shortened text			
Nygren (2019)[55]		shortened text			
Okujava (2019)[57]					
Paris (2018)[59]<br>Silva (in prep)[60]<br>Silva (2020)[61]				introduce each session with a "check-in": opportunity to do the homework and get credit for completion	change presentation of modules: no tailored sequence for each participant but the same order for all participants
Patel (2016)[63]	eventually add time limit to prevent internet addiction	shortened text			
Pinto-Bruno (2019)[64]					
Rahmadiana (2019)[66]		shortened text			
Salamanca-Sanabria (2019/ 2020)[67,68]		shortened text		add introductory videos for the training/ for some exercises	
Saulsberry (2013)[70]		shortened text			
Shala (2020)[71]	content was found to be too long: too generic	use audio recordings instead of texts as they are better understood		add components to the start of the training	
Sit (2020)[72]		use illustrations instead of text to offer	skip redundant contents/		

11. Structure	Shorten intervention	Changes in texts	More/ less repetitions	Simplify introduction	Add optional intervention elements
		encouragement or positive feedback	combine similar expressions		
Spanhel (2019)[73]	shortening length of sessions (4 shorter instead of 3 longer sessions)	shortened text		shorten introductions and overview pages	make exercises optional
Teles (2020)[75]				Visual aids to introduce the programme structure (modules and lessons) were added to reduce the burden of reading the explanatory text	
Titov (2018)[76]		shortened text			
Ünlü Ince (2013)[77]		shortened text			
Wang (2013)[79]		shortened text			
Wasil (2020)[81]	a break in the middle of the session was included	more reading material			
Yokomitsu (2020)[83]				explanations on how to use the programme are included	

12. Functionality	Provide more explanations/ examples	Access with low internet quality	Simplify navigation / computer vs. phone	Include interactive elements	Versions for specific target groups/ additional modules	Implement in a specific context
Abi Ramia (2018)[1] Harper Shehadeh (2020)[2]		low quality of internet connection as barrier for usage	user friendliness; (electronic device) illiteracy		considerations of making a version of the story for single users (single and working females)	
Abuwalla (2017)[4]					eventually adapting the parent-targeted version for Arab countries to address major stakeholders	patriarchal and religious societies as entry for implementation
Arjadi (2018a+b)[6,7]	precise examples for demanded homework exercises	pictures and comics instead of videos	use clear repetitive instructions on the program			
Bolinski (2018)[9]						
Brooks (2013)[11]					eventually develop specialised family educational and support services for family members; developed three additional, add-on modules to meet the varying needs	
Burchert (2018)[12]	provide a more practical explanation of the app's	app functions offline after initial download	still (technical) literacy limitations: simplify	increase motivation by 1) motivating messages, 2) entertaining		

12. Functionality

	Provide more explanations/ examples	Access with low internet quality	Simplify navigation / computer vs. phone	Include interactive elements	Versions for specific target groups/ additional modules	Implement in a specific context
	features and objectives	and uses minimum of mobile bandwidth	navigation with a tab bar; session displayed as a sequence of steps; increasing flexibility of the app: possibility to play the text as audio & to record audio instead of text input; mobile & web version	elements such as quizzes, 3) reminders		
Campbell (2015)[13]						
Chen (2019)[15]						
Lin (2020)[16]						
Choi (2012)[18]						
DaPonte (2018)[20]						
Eylem (2020)[22]	case examples explain how to do the exercises		can also be accessed via app			
Garabiles (2019)[24]					different designs of male and female version of intervention	
Gorman (2013)[25]				add quiz format with psychoeducative answers about different topics		hierarchic community: importance to gain tribal leaders support for intervention

12. Functionality	Provide more explanations/ examples	Access with low internet quality	Simplify navigation / computer vs. phone	Include interactive elements	Versions for specific target groups/ additional modules	Implement in a specific context
Hiratsuka (2019)[27]	information on duration was included before guided relaxation exercises		changed drag and drop functions; website rather than exclusively mobile technology			
Imamura (2019)[29]			use of smartphone only			
Ip (2016)[31]						
Sobowale (2013)[32]			low technical literacy: add page numbers for easy navigation	interactive elements to enhance engagement		social worker or peers as recommenders for intervention instead of primary health provider / parents / teacher / religious figure
Juniar (2019)[33]			change and exclude some inputs due to technical and practical issues: e.g. slide show instead of videos; mobile friendly	more interactive parts		
Kaal (2020)[35]						

12. Functionality

	Provide more explanations/ examples	Access with low internet quality	Simplify navigation / computer vs. phone	Include interactive elements	Versions for specific target groups/ additional modules	Implement in a specific context
Kanuri (2020)[37]		brief relaxation exercises were designed for limited internet connectivity environments				
Kayrouz (2015/ 2016a+b)[39–41]						
Knaevelsrud (2015)[42] Vöhringer (2020)[43]						
Wagner (2012)[44]						
Lal (2020)[46]	more audio tracks		use clear headings to structure the texts; adapt the visual display of information to make it easy to navigate (e.g. illustrate contents in tables, flowcharts)			
Lindegaard (2020)[48] Nygren (2018)[49]			technical illiteracy: eventually add possibility to have the text read out loud	eventually add free-text fields to reflect own progress; remove more interactive elements that require ongoing therapist support		

12. Functionality						
	**Provide more explanations/ examples**	**Access with low internet quality**	**Simplify navigation / computer vs. phone**	**Include interactive elements**	**Versions for specific target groups/ additional modules**	**Implement in a specific context**
Luo (2020)[50]						
Muroff (2017/ 2019)[52,53]			low literacy	increasing audio-content		
Nygren (2019)[55]	more video presentations providing explanations, e.g. of simulated client-therapist interactions					
Okujava (2019)[57]			interface was simplified (e.g. sign in)	include a chat board		
Paris (2018)[59] Silva (in prep)[60] Silva (2020)[61]			modifications to make the program accessible for people of varied educational backgrounds, computer familiarity, and literacy, e.g. audio buttons to listen to all contents and graphics; using a telenovela approach	exercises in the end of each module with combination of written or behavioural assignment		
Patel (2016)[63]				interactive features		eventually school setting for delivery of intervention; eventually adding involvement of family

12. Functionality	Provide more explanations/ examples	Access with low internet quality	Simplify navigation / computer vs. phone	Include interactive elements	Versions for specific target groups/ additional modules	Implement in a specific context
Pinto-Bruno (2019)[64]						
Rahmadiana (2019)[66]		illustrated pictures instead of videos	mobile app in addition to website			
Salamanca-Sanabria (2019/ 2020)[67,68]						
Saulsberry (2013)[70]					videos for each target group (adolescents, parents, physician/ medical staff) featuring African American and Latino persons; separate parent program	
Shala (2020)[71]				add a goal setting task/ component; add exercises (list symptoms and causes; write down activities that make feel better/ worse)		
Sit (2020)[72]						

12. Functionality	Provide more explanations/ examples	Access with low internet quality	Simplify navigation / computer vs. phone	Include interactive elements	Versions for specific target groups/ additional modules	Implement in a specific context
Spanhel (2019)[73]	explanations and illustrations for exercises and their purposes		include many audio-visual elements rather than text; link text to pictures; mobile version rather than computer based	more imagination exercises; set reminders	include module informing on where to get help	
Teles (2020)[75]			link mood assessment scores to specific lessons; add more navigational pathways to produce the same outcome; clickable items to improve navigation	introducing audio support		
Titov (2018)[76]						
Ünlü Ince (2013)[77]						
Wang (2013)[79]						
Wasil (2020)[81]						
Yokomitsu (2020)[83]			login and menu screens were slightly changed to facilitate use; use as an app			

13. Design and aesthetics

	Changes in videos/ pictures	User interface (colours)
Abi Ramia (2018)[1] Harper Shehadeh (2020)[2]	less text and story, more videos to watch; illustrations should not include cues targeting one community group over another	colour changed because of the association with a Lebanese political party
Abuwalla (2017)[4]	videos and pictures are added; include religion-neutral cultural symbols in the pictures	appealing for adolescents coloration and Arab symbols
Arjadi (2018a+b)[6,7]	characters in black and white illustrations instead of videos; add illustrations to accompany texts; culturally relevant themes for illustrations	
Bolinski (2018)[9]	Videos and pictures adapted to be more modern and culturally diverse	
Brooks (2013)[11]		
Burchert (2018)[12]	use real photos or videos instead of illustrations	narrative content screens resemble prototypical messaging apps to maximize the learnability and familiarity of the app; adjust colours (colours more attractive and suitable; reduce background colours), make the design more modern and professional
Campbell (2015)[13]	changes in pictures and videos	make the interface more relevant for native communities
Chen (2019)[15] Lin (2020)[16]	edit material to fit Asian features	
Choi (2012)[18]	culturally appropriate illustrations	
DaPonte (2018)[20]		
Eylem (2020)[22]	more visual content	
Garabiles (2019)[24]	adaptation of texts and illustrations to clearly link them, e.g. illustration about isolation: sitting alone on the bed, instead of standing away from others	bright and warm colours surrounding of main character (sun, grass, blue sky)
Gorman (2013)[25]	include pictures of Native women, babies and children to enhance identification; add a logo designed by Natives to appeal to more women in the community; modify graphics to make content more understandable; more videos	incorporating earth-tone colours
Hiratsuka (2019)[27]	some illustrations were changed to depict American Indian/ Alaska Native heritage; videos with relatable testimonials	design of the website was adapted
Imamura (2019)[29]	changed mangas	
Ip (2016)[31] Sobowale (2013)[32]	add pictures, graphics to enhance engagement	more appropriate interface to adolescents

13. Design and aesthetics		
	**Changes in videos/ pictures**	**User interface (colours)**
Juniar (2019)[33]	slide shows instead of videos; pictures should relate to Indonesia (e.g. landscape)	
Kaal (2020)[35]	design of the program logo; customized the opening image	adaptation of its graphic design
Kanuri (2020)[37]		
Kayrouz (2015/ 2016a+b)[39–41]	Arab portraits used in case examples and educational stories	
Knaevelsrud (2015)[42] Vöhringer (2020)[43]		
Wagner (2012)[44]		
Lal (2020)[46]	adapting to Canadian imagery and symbolism	more colourful design
Lindegaard (2020)[48]	use video clips to make the content more user-friendly	
Nygren (2018)[49]		
Luo (2020)[50]	images were replaced with similar depictions of Asian women; other images were changed; videos were redone with Chinese women to fit the Chinese style	
Muroff (2017/ 2019)[52,53]		
Nygren (2019)[55]	video presentations of simulated client-therapist interactions; adaptations in pictures and videos	
Okujava (2019)[57]	new videos, pictures in Georgian language and with Georgian characters	a more contemporary minimalistic design was used
Paris (2018)[59] Silva (in prep)[60]	adaptations of the videos used in the telenovela to fit the context	
Silva (2020)[61]		
Patel (2016)[63]	add visual features and animations	make it appealing to young Chinese, e.g. modern design
Pinto-Bruno (2019)[64]		
Rahmadiana (2019)[66]	changing for Indonesian pictures; illustrated pictures instead of videos	
Salamanca-Sanabria (2019/ 2020)[67,68]	increasing quality of presentation, e.g. animation instead of video	more attiring for college students
Saulsberry (2013)[70]	pictures of urban African American and Latino adolescents; avoid appearance of a "school-like" experience	increase visually appealing aspects; add thematic elements for youth; hip-hop theme and earth tone colours

**13. Design and aesthetics**		
	**Changes in videos/ pictures**	**User interface (colours)**
Shala (2020)[71]	original illustrations were minimally adapted, new illustrations were drawn for the new treatment components	
Sit (2020)[72]	change of the illustrations to fit the Chinese design (e.g. facial expressions, dress, and elements of the illustration)	
Spanhel (2019)[73]	exclude pictures linked to flight experiences, e.g. sea or boats are associated with flight rather than with relaxation; illustrate diverse people: e.g. different skin colour, nationality, names, clothes; many pictures; link text to pictures	use bright colours
Teles (2020)[75]	changing the layout for the registration page to make it more intuitive for less experienced users; changing text fonts to better distinguish the intervention elements; change pictures to be relatable to the target group	colour scheme in exercises was changed to better illustrate more appropriate/less appropriate answers; i.e. to improve feedback
Titov (2018)[76]		
Ünlü Ince (2013)[77]		
Wang (2013)[79]	pictures of Chinese figures	
Wasil (2020)[81]		add colours to emphasise certain text
Yokomitsu (2020)[83]		sky colour becomes brighter as the user proceeds in the programme (reward); colour schemes were made brighter to be more reminiscent of the Japanese anime aesthetic

**14. Guidance**			
	**Format of guidance**	**Amount of guidance**	**Person used as guide**
Abi Ramia (2018)[1]<br>Harper Shehadeh (2020)[2]	introductory call instead of email; notification before call from e-helper; choice of way of contact: phone calls, chat, email	choice of time of contact	
Abuwalla (2017)[4]	eventually add one-on-one live mentor chat or webcam session features on website		
Arjadi (2018a+b)[6,7]			guidance via phone call by non-face-to-face lay counsellors
Bolinski (2018)[9]			

14. Guidance			
	**Format of guidance**	**Amount of guidance**	**Person used as guide**
Brooks (2013)[11]	clinicians dedicated one-third of usual individual care session to reviewing the program content with the patients: reinforce educational material, review patient responses, answer questions	biweekly calls in addition to weekly videoconferencing with clinicians	
Burchert (2018)[12]		optional contact to e-helper instead of different levels of guidance; more reminders	
Campbell (2015)[13]			
Chen (2019)[15]			Chinese therapists
Lin (2020)[16]			
Choi (2012)[18]			
DaPonte (2018)[20]		automatic weekly messages (reminders, reinforcement) instead of clinician-guided	
Eylem (2020)[22]	trained e-coaches (English-Turkish or Dutch-Turkish) provide personalised feedback after each module; in case of high suicidal thoughts: phone calls to assess suicide risk and contact		
Garabiles (2019)[24]			
Gorman (2013)[25]	computer-generated, individually tailored feedback messages depending on status of women (e.g. contraception use, pregnancy status, reported alcohol use in past 2 weeks) about different topic around alcohol use and pregnancy		
Hiratsuka (2019)[27]			
Imamura (2019)[29]			
Ip (2016)[31]		more guidance requested, e.g. for exercises	
Sobowale (2013)[32]			
Juniar (2019)[33]			eCoach (Indonesian psychologist) gives feedback, appreciation and reminders
Kaal (2020)[35]			

**14. Guidance**	**Format of guidance**	**Amount of guidance**	**Person used as guide**
Kanuri (2020)[37]			
Kayrouz (2015/ 2016a+b)[39–41]	clinician-guided		
Knaevelsrud (2015)[42]<br>Vöhringer (2020)[43]			Arabic-speaking guidance by psychotherapist or psychiatrist living in different countries
Wagner (2012)[44]			
Lal (2020)[46]			
Lindegaard (2020)[48]	self-guided instead of clinician-guided		
Nygren (2018)[49]			
Luo (2020)[50]		text messages encouraging completion of online exercises and participation in the forum were sent twice per week	
Muroff (2017/ 2019)[52,53]			
Nygren (2019)[55]			guided by Kurdish psychologists living in Sweden
Okujava (2019)[57]	a more personal approach was used		physicians instead of nurses
Paris (2018)[59]			
Silva (in prep)[60]			
Silva (2020)[61]			
Patel (2016)[63]			
Pinto-Bruno (2019)[64]	participants can ask for personal contact in addition to automatic feedback		
Rahmadiana (2019)[66]			
Salamanca-Sanabria (2019/ 2020)[67,68]	personal guide (Guía personal) instead of "supporter"	23 weekly feedbacks	supporter, who was a postgraduate student in clinical psychology with experience delivering CBT for adults

14. Guidance

	Format of guidance	Amount of guidance	Person used as guide
Saulsberry (2013)[70]	motivational interview via phone by primary care provider (10-15min, baseline, after 3 months) and by research study staff (5 min, 4 times, encourage completion of intervention, behaviour change)		inclusion of local primary care provider
Shala (2020)[71]		contact on demand	
Sit (2020)[72]			
Spanhel (2019)[73]	no regular guidance but reminders		
Teles (2020)[75]		weekly reminders	
Titov (2018)[76]			
Ünlü Ince (2013)[77]			
Wang (2013)[79]			
Wasil (2020)[81]			
Yokomitsu (2020)[83]			

15. Methods used to obtain information

	Pilot/ feasibility studies	Personal interaction (focus groups, interviews, …)	Surveys/ questionnaires	Language translation procedure	Literature review
Abi Ramia (2018)[1] Harper Shehadeh (2020)[2]	feasibility study (interviews); pilot study on the adapted intervention	focus groups and interviews with experts and target group	questionnaires in the pilot study	translation into classical Arabic by a professional translator, into dialects by the author, reviewed by lay persons	
Abuwalla (2017)[4]		expert panel of physicians (practiced or raised in Arab countries)	questionnaire around the PEN-3 model [87]		on depression in Arab youths and in Arab nations
Arjadi (2018a+b)[7,6]	feasibility: feedback from health providers and patients in various steps		questionnaires on the appropriateness of the intervention	translated by the authors	on internet interventions in low-and middle-income countries
Bolinski (2018)[9]					

15. Methods used to obtain information

	Pilot/ feasibility studies	Personal interaction (focus groups, interviews, …)	Surveys/ questionnaires	Language translation procedure	Literature review
Brooks (2013)[11]	pilot study on the adapted intervention (adherence, effectiveness)	discussions with health providers and patients; interviews after usage (patients and health providers)	questionnaire		
Burchert (2018)[12]	pilot study with the adapted intervention planned	focus groups and interviews with the target group, before and after testing the app			
Campbell (2015)[13]	feasibility study with the target group (adherence and feasibility)	interviews conducted in the follow-up assessment	questionnaires (satisfaction, understandability, …)		
Chen (2019)[15]<br>Lin (2020)[16]				translation	on CBT interventions in China
Choi (2012)[18]		reviews of all materials with Chinese health providers and consumers			
DaPonte (2018)[20]	feasibility study (adherence, acceptance, effectiveness)		questionnaires on acceptance and effectiveness	2 researchers translated the material, a professional translator reviewed the translation	
Eylem (2020)[22]	pilot study on the adapted intervention (adherence, acceptance, effectiveness)	focus groups and interviews with Turkish-speaking lay people and professionals living in the Netherlands or the UK	questionnaires	forward-backward translation and checked by bilingual speakers	

**15. Methods used to obtain information**					
	**Pilot/ feasibility studies**	**Personal interaction (focus groups, interviews, …)**	**Surveys/ questionnaires**	**Language translation procedure**	**Literature review**
Garabiles (2019)[24]		focus groups with target group after a preliminary adaptation, interviews with Filipino psychologists working with the target group (2 steps)			
Gorman (2013)[25]		focus groups/ interviews with target group and health providers before and after adaptation, informal discussions with clinic staff and consultants			
Hiratsuka (2019)[27]	user testing and developmental feasibility study	focus groups with providers and patients; interviews with administrators (at various steps during the adaptation)			On potential website models
Imamura (2019)[29]	feasibility study among senior nurses	qualitative data for the process evaluation and further implementation of the program			
Ip (2016)[31] Sobowale (2013)[32]	pilot studies, also to test generalisability	focus groups with the target group and community stakeholders after looking at the intervention; expert panel on the first version of the adapted intervention	questionnaire on usability, structure, content and cultural appropriateness		

15. Methods used to obtain information

	Pilot/ feasibility studies	Personal interaction (focus groups, interviews, …)	Surveys/ questionnaires	Language translation procedure	Literature review
Juniar (2019)[33]	feasibility study planned to test satisfaction, usability, adherence and effectiveness	focus groups with target group on the unadapted intervention content, interviews with Indonesian psychologists on the first version of the adapted intervention		translation into English, then to Indonesia by a professional translator (back translation is planned)	in order to find a term that represents stress in Indonesian culture
Kaal (2020)[35]	limited pilot trial for potential users, including family doctors (written feedback)		web-survey with alcohol users to find a program name	translation and edition of text by native speakers	on the opinions, attitudes and behaviour of the target group; on a potential programme to adapt
Kanuri (2020)[37]	usability, acceptability, and feasibility trial		online survey to assess students' mental health		
Kayrouz (2015/ 2016a+b)[39–41]	pilot studies (3)	focus groups	questionnaires (recommendation, worth doing the course); online survey (key themes for case examples and educational stories)	process of translation 1) 5 professional translators translated English into Arabic, 2) review of an independent translation team (professional translator, community bilingual speaker), 3) review of the lead author, 4) changes were discussed by all stakeholders to reach consensus.	on acculturation and Arabs

15. Methods used to obtain information

	Pilot/ feasibility studies	Personal interaction (focus groups, interviews, …)	Surveys/ questionnaires	Language translation procedure	Literature review
Knaevelsrud (2015)[42] Vöhringer (2020)[43] Wagner (2012)[44]	pilot study			Translations were conducted by different native-speaking psychotherapists following the guidelines for cross-cultural translations	
Lal (2020)[46]	planned pilot test of the adapted platform with a small sample of mental health service users	focus groups, meetings with target group and providers before the adaptation	surveys and feedback forms during various stages		on frameworks on cultural adaptation of interventions
Lindegaard (2020)[48] Nygren (2018)[49]	pilot study (target group)	focus groups (professionals and target group)	questionnaire (content understandable, helpful, and planned to continue using)		
Luo (2020)[50]	pilot test to evaluate whether the intervention was clear and understandable			forward-backward translation by two bilingual Mandarin-English-speakers	
Muroff (2017/ 2019)[53,52]		review of the adapted material by Latino staff and program clients; adaptation in discussion of a team of bilingual Spanish speakers under supervision of a cultural expert			
Nygren (2019)[55]				validation of language translation by consultation with and proof-reading by a	followed a qualitative description of important stressors for

15. Methods used to obtain information

	Pilot/ feasibility studies	Personal interaction (focus groups, interviews, …)	Surveys/ questionnaires	Language translation procedure	Literature review
				Kurdish-speaking author and high-school teacher	Kurds living in Sweden [88]
Okujava (2019)[57]		interviews		forward-backward translation	
Paris (2018)[59] Silva (in prep)[60] Silva (2020)[61]		various discussions among bilingual investigators and clinical program leadership serving the target group	questionnaires on satisfaction with the adapted intervention		
Patel (2016)[63]	pilot study (cultural relevance and effectiveness)	expert panel (interview)	feedback surveys via mail (format and socio-cultural relevance of the intervention); effectiveness measures		
Pinto-Bruno (2019)[64]		review of the adaptation of staff		translation by an official translator	
Rahmadiana (2019)[66]	feasibility study to test user's satisfaction, usability and uptake as well as effectiveness of the adapted intervention	online focus groups with students to gather information on the cultural appropriateness of the intervention's content (before the adaptation)		forward-backward translation procedure	
Salamanca-Sanabria (2019/ 2020)[67,68]	feasibility study with Columbian college students; feedback of experts in the target country after completing the intervention		cultural relevance questionnaire	Translated by professional translators and reviewed several times	on frameworks for culturally adapting face to face psychotherapy

15. Methods used to obtain information	Pilot/ feasibility studies	Personal interaction (focus groups, interviews, …)	Surveys/ questionnaires	Language translation procedure	Literature review
Saulsberry (2013)[70]		focus groups with target group, interviews with parents and healthcare provider			on vulnerability and protective factors for depressive episodes of Latino and Afro-American adolescents as compared to Caucasian adolescents
Shala (2020)[71]	feasibility trial	focus groups, interviews to examine the populations' cultural concepts of distress, usability trial		use of different translation methods	on how to address fatalism and enhance treatment motivation among culturally diverse groups
Sit (2020)[72]	planned	focus groups, interviews		backward-forward-translation	
Spanhel (2019)[73]	pilot study to test the culturally adapted internet intervention	interviews with healthcare providers, usability study (think aloud) with target group			on conducted cultural adaptation of internet interventions
Teles (2020)[75]	planned	fidelity check after adaptation by program authors; focus groups, interviews, usability trial		translation from English to Portuguese by authorised translator which was checked by health professionals	review of empirical studies on characteristics and psychosocial needs of dementia caregivers
Titov (2018)[76]		consultation with Indigenous people and reference groups			
Ünlü Ince (2013)[77]		interview with 2 Turkish persons			

15. Methods used to obtain information					
	**Pilot/ feasibility studies**	**Personal interaction (focus groups, interviews, …)**	**Surveys/ questionnaires**	**Language translation procedure**	**Literature review**
Wang (2013)[79]				forward-backward translation by bilingual authors	
Wasil (2020)[81]	pilot test	focus groups with Indian high school students and school administrators			
Yokomitsu (2020)[83]					

16. Persons involved in the adaptation process			
	**Target group and associated people**	**Professionals working with the target group**	**Professionals not working with the target group**
Abi Ramia (2018)[1]<br>Harper Shehadeh (2020)[2]	✓	✓	✓ (translator)
Abuwalla (2017)[4]		✓	
Arjadi (2018a+b)[6,7]	✓	✓	✓ (technologist)
Bolinski (2018)[9]	✓		
Brooks (2013)[11]	✓	✓	✓ (technologist)
Burchert (2018)[12]	✓		
Campbell (2015)[13]	✓		
Chen (2019)[15]<br>Lin (2020)[16]	✓	✓	
Choi (2012)[18]	✓	✓	
DaPonte (2018)[20]	✓		✓ (professional translator)
Eylem (2020)[22]	✓	✓	✓ (bilingual speakers)
Garabiles (2019)[24]	✓	✓	
Gorman (2013)[25]	✓	✓	
Hiratsuka (2019)[27]	✓	✓	✓ (administrative leader)
Imamura (2019)[29]	✓	✓	
Ip (2016)[31]<br>Sobowale (2013)[32]	✓	✓	
Juniar (2019)[33]	✓	✓	✓ (professional translator)
Kaal (2020)[35]	✓	✓	✓ (text and visual material experts)
Kanuri (2020)[37]	✓	✓	✓ (software engineers)
Kayrouz (2015/ 2016a+b)[39–41]	✓	✓	✓ (professional translators)
Knaevelsrud (2015)[42]	✓		✓ (translator)

16. Persons involved in the adaptation process

	Target group and associated people	Professionals working with the target group	Professionals not working with the target group
Vöhringer (2020)[43]			
Wagner (2012)[44]			
Lal (2020)[46]	✓	✓	
Lindegaard (2020)[48]<br>Nygren (2018)[49]	✓	✓	
Luo (2020)[50]		✓	✓ (Chinese game development team)
Muroff (2017/ 2019)[52,53]	✓	✓	✓ (professional translator)
Nygren (2019)[55]		✓	
Okujava (2019)[57]	✓	✓	
Paris (2018)[59]<br>Silva (in prep)[60]<br>Silva (2020)[61]	✓	✓	✓ (actors and film director)
Patel (2016)[63]	✓	✓	
Pinto-Bruno (2019)[64]		✓	✓ (professional translator)
Rahmadiana (2019)[66]	✓	✓	✓ (translation team)
Salamanca-Sanabria (2019/ 2020)[67,68]	✓	✓	✓ (professional translators)
Saulsberry (2013)[70]	✓	✓	
Shala (2020)[71]	✓	✓	✓ (independent translator)
Sit (2020)[72]	✓	✓	✓ (translators, professional illustrator)
Spanhel (2019)[73]	✓	✓	
Teles (2020)[75]	✓	✓	✓ (professional translators)
Titov (2018)[76]	✓	✓	
Ünlü Ince (2013)[77]	✓	✓	
Wang (2013)[79]			✓ (authors)
Wasil (2020)[81]	✓	✓	
Yokomitsu (2020)[83]		✓	

17. Theoretical framework

	Guideline for cultural adaptation of face-to-face treatment
Abi Ramia (2018)[1]<br>Harper Shehadeh (2020)[2]	Hwang, W. C. (2009). The formative method for adapting psychotherapy (FMAP): a community-based developmental approach to culturally adapting therapy. Professional Psychology: Research and Practice, 40(4), 369–377.
Abuwalla (2017)[4]	Airhihenbuwa, C. O. (1990). A conceptual model for culturally appropriate health education programs in developing countries. International Quarterly of Community Health Education, 11(1), 53–62. PEN-3 cultural model

17. Theoretical framework	
	**Guideline for cultural adaptation of face-to-face treatment**
Arjadi (2018a+b)[6,7]	Hwang, W. C. (2009). The formative method for adapting psychotherapy (FMAP): a community-based developmental approach to culturally adapting therapy. Professional Psychology: Research and Practice, 40(4), 369–377 ; Bernal, G., Bonilla, J., & Bellido, C. (1995). Ecological validity and cultural sensitivity for outcome research - issues for the cultural-adaptation and development of psychosocial treatments with hispanics. Journal of Abnormal Child Psychology, 23(1), 67–82. Cultural Sensitivity Framework for Interventions
Bolinski (2018)[9]	
Brooks (2013)[11]	Barrera, M., & Castro, F. G. (2006). A heuristic framework for the cultural adaptation of interventions. Clinical Psychology: Science and Practice, 13(4), 311–316.
Burchert (2018)[12]	
Campbell (2015)[13]	
Chen (2019)[15]	
Lin (2020)[16]	
Choi (2012)[18]	
DaPonte (2018)[20]	
Eylem (2020)[22]	Bernal, G., Bonilla, J., & Bellido, C. (1995). Ecological validity and cultural sensitivity for outcome research - issues for the cultural-adaptation and development of psychosocial treatments with hispanics. Journal of Abnormal Child Psychology, 23(1), 67–82. Cultural Sensitivity Framework for Interventions
Garabiles (2019)[24]	Manson, S. M. (1997). Cross-cultural and multiethnic assessment of trauma. In J. P. Wilson & T. M. Keane (Eds.), Assessing psychological trauma and PTSD (pp. 239–266). New York, USA: Guildford Press ; van Ommeren, M., Sharma, B., Thapa, S., Makaju, R., Prasain, D., Bhattarai, R., & de Jong, J. (1999). Preparing instruments for transcultural research: use of the translation monitoring form with Nepali-speaking Bhutanese refugees. Transcultural Psychiatry, 36(3), 285–301.
Gorman (2013)[25]	
Hiratsuka (2019)[27]	
Imamura (2019)[29]	
Ip (2016)[31]<br>Sobowale (2013)[32]	Wingood, G. M., & Diclemente, R. J. (2008). The ADAPT-ITT model: A novel method of adapting evidence-based HIV interventions. Journal of Acquired Immune Deficiency Syndromes, 47(SUPPL. 1), 40–46 ; McKleroy, V. S., Galbraith, J. S., Cummings, B., Jones, P., Harshbarger, C., Collins, C., … Carey, J. W. (2006). Adapting evidence-based behavioral interventions for new settings and target populations. AIDS Education and Prevention, 18(SUPPL. A), 59–73
Juniar (2019)[33]	Barrera, M., Castro, F. G., Strycker, L. A., & Toobert, D. J. (2013). Cultural adaptations of behavioral health interventions: a progress report. Journal of Consulting and Clinical Psychology, 81(2), 196–205.
Kaal (2020)[35]	
Kanuri (2020)[37]	
Kayrouz (2015/ 2016a+b)[39–41]	
Knaevelsrud (2015)[42]	
Vöhringer (2020)[43]	
Wagner (2012)[44]	

17. Theoretical framework	
	**Guideline for cultural adaptation of face-to-face treatment**
Lal (2020)[46]	created an own approach (eHealth adaptation framework) based on various previous frameworks (combination of cultural adaptation and internet-based adaptations): Castro, F. G., Barrera, M., & Steiker, L. K. H. (2010). Issues and challenges in the design of culturally adapted evidence-based interventions. Annual Reviews of Clinical Psychology, 6, 213–239 ; Bernal, G., Bonilla, J., & Bellido, C. (1995). Ecological validity and cultural sensitivity for outcome research - issues for the cultural-adaptation and development of psychosocial treatments with hispanics. Journal of Abnormal Child Psychology, 23(1), 67–82. Cultural Sensitivity Framework for Interventions
Lindegaard (2020)[48]<br>Nygren (2018)[49]<br>Luo (2020)[50]<br>Muroff (2017/ 2019)[52,53]<br>Nygren (2019)[55]<br>Okujava (2019)[57]	Chu, J., & Leino, A. (2017). Advancement in the maturing science of cultural adaptations of evidence-based interventions. Journal of Consulting and Clinical Psychology, 85(1), 45–57. Cultural Treatment Adaptation Framework (CTAF)
Paris (2018)[59]<br>Silva (in prep)[60]<br>Silva (2020)[61]	Castro, F. G., Barrera, M., & Steiker, L. K. H. (2010). Issues and challenges in the design of culturally adapted evidence-based interventions. Annual Reviews of Clinical Psychology, 6, 213–239
Patel (2016)[63]	Kreuter, M. W., Lukwago, S. N., Bucholtz, D. C., Clark, E. M., & Sanders-Thompson, V. (2003). Achieving cultural appropriateness in health promotion programs: targeted and tailored approaches. Health Education and Behavior, 30(2), 133–146
Pinto-Bruno (2019)[64]	WHO: Standardized guide for translation and adaptation of the WHO (mentioned in Pot, A. M., Gallagher-Thompson, D., Xiao, L. D., Willemse, B. M., Rosier, I., Mehta, K. M., … iSupport development team. (2019). iSupport: a WHO global online intervention for informal caregivers of people with dementia. World Psychiatry, 18(3), 365–366)
Rahmadiana (2019)[66]	Barrera, M., Castro, F. G., Strycker, L. A., & Toobert, D. J. (2013). Cultural adaptations of behavioral health interventions: a progress report. Journal of Consulting and Clinical Psychology, 81(2), 196–205.
Salamanca-Sanabria (2019/ 2020)[67,68]	Resnicow, K., Soler, R., Braithwaite, R. L., Ahluwalia, J. S., & Butler, J. (2000). Cultural sensitivity in substance use prevention. Journal of Community Psychology, 28(3), 271–290 ; Helms, J. E. (2015). An examination of the evidence in culturally adapted evidence-based or empirically supported interventions. Transcultural Psychiatry, 52(2), 174–197 ; Bernal, G., Bonilla, J., & Bellido, C. (1995). Ecological validity and cultural sensitivity for outcome research - issues for the cultural-adaptation and development of psychosocial treatments with hispanics. Journal of Abnormal Child Psychology, 23(1), 67–82. Cultural Sensitivity Framework for Interventions
Saulsberry (2013)[70]	Airhihenbuwa, C. O. (1990). A conceptual model for culturally appropriate health education programs in developing countries. International Quarterly of Community Health Education, 11(1), 53–62. PEN-3 cultural model ; Kreuter, M. W., Lukwago, S. N., Bucholtz, D. C., Clark, E. M., & Sanders-Thompson, V. (2003). Achieving cultural appropriateness in health promotion programs: targeted and tailored approaches. Health Education and Behavior, 30(2), 133–146
Shala (2020)[71]	Heim, E., & Kohrt, B. A. (2019). Cultural adaptation of scalable psychological interventions: a new conceptual framework. Clinical Psychology in Europe, 1(4), e37679. ; Resnicow, K., Baranowski, T., Ahluwalia, J. S., & Braithwaite, R. (1999). Cultural sensitivity in public health: defined and demystified. Ethnicity & Disease, 9(1), 10–21.
Sit (2020)[72]	Bernal, G., Bonilla, J., & Bellido, C. (1995). Ecological validity and cultural sensitivity for outcome research - issues for the cultural-adaptation and development of

17. Theoretical framework

	**Guideline for cultural adaptation of face-to-face treatment**
	psychosocial treatments with hispanics. Journal of Abnormal Child Psychology, 23(1), 67–82. Cultural Sensitivity Framework for Interventions
Spanhel (2019)[73]	Barrera, M., & Castro, F. G. (2006). A heuristic framework for the cultural adaptation of interventions. Clinical Psychology: Science and Practice, 13(4), 311–316
Teles (2020)[75]	WHO: Standardized guide for translation and adaptation of the WHO (mentioned in Pot, A. M., Gallagher-Thompson, D., Xiao, L. D., Willemse, B. M., Rosier, I., Mehta, K. M., … iSupport development team. (2019). iSupport: a WHO global online intervention for informal caregivers of people with dementia. World Psychiatry, 18(3), 365–366)
Titov (2018)[76]	
Ünlü Ince (2013)[77]	
Wang (2013)[79]	
Wasil (2020)[81]	
Yokomitsu (2020)[83]	

Supplementary Table 6: Results of the post hoc conducted analyses

The following post hoc analyses were conducted to investigate potential links between characteristics of the internet- and mobile-based interventions (IMI) and the extent of cultural adaptation:

1) Pearson's correlation coefficient: duration of the IMI/ guidance of the IMI and extent of overall/ content/ methodological/ procedural cultural adaptation (Table 6.1)
2) Analysis of variance: target group of the IMI and extent of overall/ content/ method/ procedural cultural adaptation (Table 6.2)

Table 6.1: Pearson's correlation coefficients of selected characteristics of the 42 internet- and mobile-based interventions (IMI) and the extent of (specific components of) cultural adaptation (CA).

	Extent of CA	Extent of content CA	Extent of method CA	Extent of procedure CA
IMI: number of modules	*r*(40)=-.08	*r*(40)=-.11	*r*(40)=-.06	*r*(40)=.00
IMI: guidance (yes/ no)	*r*(40)=.09	*r*(40)=.12	*r*(40)=.08	*r*(40)=.00

All correlations are non-significant with p>.05.

Table 6.2: Means (standard deviations) of the conducted extent of cultural adaptation, separated by the target group of the used internet- and mobile-based intervention.

	Extent of CA (Max: 17)	Extent of content CA (Max: 10)	Extent of method CA (Max: 4)	Extent of procedure CA (Max: 3)
LMIC (n=10)	12.0 (3.3)	6.9 (2.0) [2]	2.9 (1.0)	2.2 (0.9)
Immigrants (n=14)	13.6 (3.2) [1]	8.4 (1.6) [3]	3.1 (1.3)	2.1 (1.1)
Indigenous (n=5)	11.2 (2.8)	6.6 (1.7)	2.6 (1.1)	2.0 (0.7)
HIC (n=13)	9.5 (4.1) [1]	5.2 (2.2) [2, 3]	2.4 (1.0)	1.7 (1.3)
	F(3,38)=3.32, p=.033	F(3,38)=6.09, p=.002	F(3,38)=0.97, p=.419	F(3,38)=0.53, p=.667

CA cultural adaptation, *LMIC* low and middle income country, *HIC* high income country

[1] pairwise comparison, p<.05, [2] pairwise comparison, p<.05, [3] pairwise comparison, p<.001

The following post hoc analyses were conducted to investigate potential links between characteristics of the IMI/ extent of cultural adaptation and the effectiveness/ adherence of the IMI:

1) Pearson’s correlation coefficient: duration of the IMI/ guidance of the IMI/ extent of overall cultural adaptation and effectiveness/ adherence (percentage of completers) of the IMI (Table 6.3)
2) Analysis of variance: target group of the IMI and effectiveness/ adherence (percentage of completers) of the IMI (Table 6.4)

Table 6.3: Pearson’s correlation coefficients of selected characteristics of the internet- and mobile-based interventions (IMI), the extent of cultural adaptation, and outcome measures of the culturally adapted IMI.

	Effect size (Hedges’s g) n=15	Adherence (% completers) n=11
Extent of cultural adaptation	*r*(13)=-.17	*r*(9)=-.32
IMI: amount of modules	*r*(13)=.20	*r*(9)=-.35
IMI: guidance (yes/ no)	*r*(13)=-.07	*r*(9)=-.20

All correlations are non-significant with p>.05.

Table 6.4: Means (standard deviations) of the effectiveness and adherence of the culturally adapted internet- and mobile-based interventions examined in randomised controlled trials included in the review, separated by the respective target group.

	Effect size (Hedges’ g)	Adherence (% completers)
LMIC	0.74 (0.30), n=3	34.40 (35.50), n=2
Immigrants	0.67 (0.45), n=7	43.78 (27.82), n=6
HIC	1.00 (1.00), n=5	46.37 (35.81), n=3
	F(2,12)=0.39, p=.683	F(2,8)=0.10, p=.909

LMIC people living in low and middle income country, *HIC* people living in high income country

Supplementary References

1. Abi Ramia, J. *et al.* Community cognitive interviewing to inform local adaptations of an e-mental health intervention in Lebanon. *Glob. Ment. Heal.* **5,** e39 (2018).
2. Harper Shehadeh, M. J. *et al.* Step-by-Step, an e-mental health intervention for depression: a mixed methods pilot study from Lebanon. *Front. Psychiatry* **10,** 986 (2020).
3. Carswell, K. *et al.* Step-by-Step: a new WHO digital mental health intervention for depression. *Mhealth* **4,** 1–7 (2018).
4. Abuwalla, Z. *et al.* Proposed model for the cultural adaptation of an Internet-based depression prevention intervention (CATCH-IT) for Arab adolescents. *Int. J. Adolesc. Med. Heal.* **31,** 1–24 (2017).
5. Voorhees, B. W. Van *et al.* Randomized clinical trial of an Internet-based depression prevention program for adolescents (Project CATCH-IT) in primary care: twelve-week outcomes. *J. Dev. Behav. Pediatr.* **30,** 23–37 (2009).
6. Arjadi, R., Nauta, M. H., Suryani, A. O. & Bockting, C. L. H. Guided Act and Feel Indonesia - Internet-based behavioral activation intervention for depression in Indonesia: a systematic cultural adaptation. *Makara Hubs-Asia* **22,** 3–11 (2018).
7. Arjadi, R. *et al.* Internet-based behavioural activation with lay counsellor support versus online minimal psychoeducation without support for treatment of depression: a randomised controlled trial in Indonesia. *The Lancet Psychiatry* **5,** 707–716 (2018).
8. Bockting, C. L. H. & van Valen, E. Act and feel online: Internet-based behavioral activation in Dutch. *unpublished*
9. Bolinski, F. *et al.* Effectiveness of a transdiagnostic individually tailored Internet-based and mobile-supported intervention for the indicated prevention of depression and anxiety (ICare Prevent) in Dutch college students: study protocol for a randomised controlled trial. *Trials* **19,** 1–13 (2018).
10. Buntrock, C. *et al.* Effectiveness of a web-based cognitive behavioural intervention for subthreshold depression: pragmatic randomised controlled trial. *Psychother. Psychosom.* **84,** 348–358 (2015).
11. Brooks, L. A., Bloomer, M. J. & Manias, E. Culturally sensitive communication at the end-of-life in the intensive care unit: a systematic review. *Aust. Crit. Care* **32,** 516–523 (2019).
12. Burchert, S. *et al.* User-centered app adaptation of a low-intensity e-mental health intervention for Syrian refugees. *Front. Psychiatry* **9,** 663 (2019).
13. Campbell, A. N. C. *et al.* Acceptability of a web-based community reinforcement approach for substance use disorders with treatment-seeking American Indians/Alaska Natives. *Community Ment. Health J.* **51,** 393–403 (2015).
14. Bickel, W. K., Marsch, L. A., Buchhalter, A. R. & Badger, G. J. Computerized behavior therapy for opioid-dependent outpatients: a randomized controlled trial. *Exp. Clin. Psychopharmacol.* **16,** 132–143 (2008).
15. Chen, H. *et al.* Predictors of treatment outcomes and adherence in internet-based cognitive behavioral therapy for social anxiety in China. *Behav. Cogn. Psychother.* 1–13 (2019). doi:10.1017/S1352465819000730
16. Lin, L. Y. *et al.* An internet-based intervention for individuals with social anxiety and different levels of Taijin Kyofusho in China. *J. Cross. Cult. Psychol.* **51,** 387–402 (2020).
17. Berger, T. *et al.* Internet-based treatment of social phobia: a randomized controlled trial comparing unguided with two types of guided self-help. *Behav. Res. Ther.* **49,** 158–169 (2011).
18. Choi, I. *et al.* Culturally attuned internet treatment for depression amongst Chinese Australians: a randomised controlled trial. *J. Affect. Disord.* **136,** 459–468 (2012).
19. Perini, S., Titov, N. & Andrews, G. Clinician-assisted Internet-based treatment is effective for depression: randomized controlled trial. *Aust. N. Z. J. Psychiatry* **43,** 571–578 (2009).
20. Daponte, D. *et al.* Facilitating the dissemination of iCBT for the treatment of anxiety and depression: a feasibility study. *Behav. Chang.* **35,** 139–151 (2018).
21. Titov, N. *et al.* Transdiagnostic internet treatment for anxiety and depression: a randomised controlled trial. *Behav. Res. Ther.* **49,** 441–452 (2011).
22. Eylem, O. *et al.* Reducing suicidal ideation among Turkish migrants in the Netherlands and in the UK: a pilot RCT of a guided online intervention. Preprint at https://www.researchsquare.com/article (2020).
23. Van Spijker, B. A. J., Van Straten, A. & Kerkhof, A. J. F. M. Effectiveness of online self-help for suicidal thoughts: results of a randomised controlled trial. *PLoS One* **9,** e90118 (2014).
24. Garabiles, M. R., Harper Shehadeh, M. & Hall, B. J. Cultural adaptation of a scalable World Health Organization e-mental health program for overseas Filipino workers. *JMIR Form. Res.* **3,** e11600 (2019).
25. Gorman, J. R. *et al.* Creating a culturally appropriate web-based behavioral intervention for American Indian/ Alaska native women in Southern California: the healthy women healthy native nation study. *Am. Indian Alaska Nativ. Ment. Heal. Res.* **20,** 1–15 (2013).
26. Delrahim-Howlett, K. *et al.* Web-based assessment and brief intervention for alcohol use in women of childbearing potential: a report of the primary findings. *Alcohol. Clin. Exp. Res.* **35,** 1331–1338 (2011).
27. Hiratsuka, V. Y. *et al.* An internet-based therapeutic tool for American Indian/Alaska native adults with posttraumatic stress disorder: user testing and developmental feasibility study. *J. Med. Internet Res.* **21,** (2019).
28. Kuhn, E. *et al.* Preliminary evaluation of PTSD Coach, a smartphone app for post-traumatic stress symptoms. *Mil.*

Med. **179,** 12–18 (2014).
29. Imamura, K. *et al.* Effects of two types of smartphone-based stress management programmes on depressive and anxiety symptoms among hospital nurses in Vietnam: a protocol for three-arm randomised controlled trial. *BMJ Open* **9,** e025138 (2019).
30. Sakuraya, A., Imamura, K. & Kagami, H. Which e-learning stress management program improve psychological distress most?: a multi-arm randomized controlled trial. in *The 90th Annual Meeting of Japan Society for Occupational Health* 327 (Japan Society for Occupational Health, 2017).
31. Ip, P. *et al.* Effectiveness of a culturally attuned Internet-based depression prevention program for Chinese adolescents: a randomized controlled trial. *Depress. Anxiety* **33,** 1123–1131 (2016).
32. Sobowale, K. *et al.* Adaptation of an internet-based depression prevention intervention for Chinese adolescents: From ‘ CATCH-IT’ to ‘grasp the opportunity’. *Int. J. Adolesc. Med. Health* **25,** 127–137 (2013).
33. Juniar, D. *et al.* Web-based stress management program for university students in Indonesia: systematic cultural adaptation and protocol for a feasibility study. *JMIR Res. Protoc.* **8,** e11493 (2019).
34. Heber, E., Lehr, D., Ebert, D. D., Berking, M. & Riper, H. Web-based and mobile stress management intervention for employees: a randomized controlled trial. *J. Med. Internet Res.* **18,** e21 (2016).
35. Kaal, E. *et al.* Testing the efficacy of a minimal-guidance online self-help intervention for alcohol misuse in Estonia: study protocol of a randomized controlled trial. *BMC Public Health* **20,** 790 (2020).
36. Schaub, M. P. *et al.* Efficacy of an internet-based self-help intervention to reduce co-occurring alcohol misuse and depression symptoms in adults: study protocol of a three-arm randomised controlled trial. *BMJ Open* **6,** 1–13 (2016).
37. Kanuri, N. *et al.* Examining the initial usability, acceptability and feasibility of a digital mental health intervention for college students in India. *Int. J. Psychol.* **55,** 657–673 (2020).
38. Kanuri, N. *et al.* The feasibility, acceptability, and efficacy of delivering internet-based self-help and guided self-help interventions for generalized anxiety disorder to Indian university students: design of a randomized controlled trial. *JMIR Res. Protoc.* **4,** e136 (2015).
39. Kayrouz, R. *et al.* A pilot study of self-guided internet-delivered cognitive behavioural therapy for anxiety and depression among Arabs. *Internet Interv.* **3,** 18–24 (2016).
40. Kayrouz, R., Dear, B. F., Karin, E., Fogliati, V. J. & Titov, N. A pilot study of a clinician-guided internet-delivered cognitive behavioural therapy for anxiety and depression among Arabs in Australia, presented in both English and Arabic languages. *Internet Interv.* **5,** 5–11 (2016).
41. Kayrouz, R. *et al.* A feasibility open trial of guided Internet-delivered cognitive behavioural therapy for anxiety and depression amongst Arab Australians. *Internet Interv.* **2,** 32–38 (2015).
42. Knaevelsrud, C., Brand, J., Lange, A., Ruwaard, J. & Wagner, B. Web-based psychotherapy for posttraumatic stress disorder in war-traumatized Arab patients: randomized controlled trial. *J. Med. Internet Res.* **17,** e71 (2015).
43. Vöhringer, M. *et al.* Should I stay or must I go? Predictors of dropout in an internet-based psychotherapy programme for posttraumatic stress disorder in Arabic. *Eur. J. Psychotraumatol.* **11,** (2020).
44. Wagner, B., Schulz, W. & Knaevelsrud, C. Efficacy of an Internet-based intervention for posttraumatic stress disorder in Iraq: a pilot study. *Psychiatry Res.* **195,** 85–88 (2012).
45. Lange, A. *et al.* Interapy: a controlled randomized trial of the standardized treatment of posttraumatic stress through the internet. *J. Consult. Clin. Psychol.* **71,** 901–909 (2003).
46. Lal, S. *et al.* Adaptation of a digital health innovation to prevent relapse and support recovery in youth receiving services for first-episode psychosis: results from the Horyzons-Canada phase 1 study. *JMIR Form. Res.* **4,** e19887 (2020).
47. Alvarez-Jimenez, M. *et al.* On the HORYZON: moderated online social therapy for long-term recovery in first episode psychosis. *Schizophr. Res.* **143,** 143–149 (2013).
48. Lindegaard, T. *et al.* Internet-based cognitive behavioural therapy for depression and anxiety among Arabic-speaking individuals in Sweden: a pilot randomized controlled trial. *Cogn. Behav. Ther.* **50,** 47–66 (2020).
49. Nygren, T., Berg, M., Sarkohi, A. & Andersson, G. Development of an Internet-based cognitive behavioral therapy self-help program for arabic-speaking immigrants: mixed-methods study. *JMIR Res. Protoc.* **7,** e11872 (2018).
50. Luo, Y. J., Jackson, T., Stice, E. & Chen, H. Effectiveness of an internet dissonance-based eating disorder prevention intervention among body-dissatisfied young Chinese women. *Behav. Ther.* (2020). doi:10.1016/j.beth.2020.04.007
51. Stice, E., Rohde, P., Durant, S. & Shaw, H. A preliminary trial of a prototype internet dissonance-based eating disorder prevention program for young women with body image concerns. *J. Consult. Clin. Psychol.* **80,** 907–916 (2012).
52. Muroff, J. *et al.* Use of a smartphone recovery tool for Latinos with co-occurring alcohol and other drug disorders and mental disorders. *J. Dual Diagn.* **13,** 280–290 (2017).
53. Muroff, J. *et al.* An outcome study of the CASA-CHESS smartphone relapse prevention tool for Latinx Spanish-speakers with substance use disorders. *Subst. Use Misuse* **54,** 1438–1449 (2019).
54. McTavish, F. M., Chih, M. Y., Shah, D. & Gustafson, D. H. How patients recovering from alcoholism use a smartphone intervention. *J. Dual Diagn.* **8,** 294–304 (2008).
55. Nygren, T. *et al.* Internet-based treatment of depressive symptoms in a Kurdish population: a randomized

controlled trial. *J. Clin. Psychol.* **75,** 985–998 (2019).
56. Andersson, G. *et al.* Internet-based self-help for depression: randomised controlled trial. *Br. J. Psychiatry* **187,** 456–461 (2005).
57. Okujava, N. *et al.* Digital cognitive behavioral therapy for insomnia – The first Georgian version. Can we use it in practice? *Internet Interv.* **17,** 100244 (2019).
58. Van Straten, A. *et al.* Guided Internet-delivered cognitive behavioural treatment for insomnia: a randomized trial. *Psychol. Med.* **44,** 1521–1532 (2014).
59. Paris, M. *et al.* Culturally adapted, web-based cognitive behavioral therapy for Spanish-speaking individuals with substance use disorders: a randomized clinical trial. *Am. J. Public Health* **108,** 1535–1542 (2018).
60. Silva, M. A. *et al.* Computer-based training for cognitive behavioral therapy for Spanish-speaking substance users: adaptation and satisfaction.
61. Silva, M. A. *et al.* Changes in DSM criteria following a culturally-adapted computerized CBT for Spanish-speaking individuals with substance use disorders. *J. Subst. Abuse Treat.* **110,** 42–48 (2020).
62. Carroll, K. M. *et al.* Computer-assisted delivery of cognitive-behavioral therapy for addiction: a randomized trial of CBT4CBT. *Am. J. Psychiatry* **165,** 881–888 (2008).
63. Patel, U. *et al.* Cultural considerations for the adaptation of an Internet-based intervention for depression prevention in Mainland China. *Int. J. Adolesc. Med. Health* **29,** 20150099 (2017).
64. Pinto-Bruno, Á. C., Pot, A. M., Kleiboer, A., Droes, R.-M. & van Straten, A. An online minimally guided intervention to support family and other unpaid carers of people with dementia: protocol for a randomized controlled trial. *JMIR Res. Protoc.* **8,** e14106 (2019).
65. Pot, A. M. *et al.* iSupport: a WHO global online intervention for informal caregivers of people with dementia. *World Psychiatry* **18,** 365–366 (2019).
66. Rahmadiana, M. *et al.* Guided internet-based transdiagnostic intervention for Indonesian university students with symptoms of anxiety and depression: a pilot study protocol. *Internet Interv.* **15,** 28–34 (2019).
67. Salamanca-Sanabria, A., Richards, D. & Timulak, L. Adapting an internet-delivered intervention for depression for a Colombian college student population: an illustration of an integrative empirical approach. *Internet Interv.* **15,** 76–86 (2019).
68. Salamanca-Sanabria, A. *et al.* A culturally adapted cognitive behavioral internet-delivered intervention for depressive symptoms: randomized controlled trial. *JMIR Ment. Heal.* **7,** e13392 (2020).
69. Richards, D. *et al.* A randomized controlled trial of an internet-delivered treatment: its potential as a low-intensity community intervention for adults with symptoms of depression. *Behav. Res. Ther.* **75,** 20–31 (2015).
70. Saulsberry, A. *et al.* Chicago urban resiliency building (CURB): an internet-based depression-prevention intervention for urban African-American and Latino adolescents. *J. Child Fam. Stud.* **22,** 150–160 (2013).
71. Shala, M. *et al.* Cultural adaptation of Hap-pas-Hapi, an internet and mobile-based intervention for the treatment of psychological distress among Albanian migrants in Switzerland and Germany. *Internet Interv.* **21,** 100339 (2020).
72. Sit, H. F. *et al.* The cultural adaptation of Step-by-Step: an intervention to address depression among Chinese young adults. *Front. Psychiatry* **11,** 650 (2020).
73. Spanhel, K. *et al.* Cultural adaptation of internet interventions for refugees: results from a user experience study in Germany. *Internet Interv.* **18,** 100252 (2019).
74. Thiart, H., Lehr, D., Ebert, D. D., Berking, M. & Riper, H. Log in and breathe out: Internet-based recovery training for sleepless employees with work-related strain – results of a randomized controlled trial. *Scand. J. Work. Environ. Heal.* **41,** 164–174 (2015).
75. Teles, S., Napolskij, M. S., Paúl, C., Ferreira, A. & Seeher, K. Training and support for caregivers of people with dementia: The process of culturally adapting the World Health Organization iSupport programme to Portugal. *Dementia* (2020). doi:10.1177/1471301220910333
76. Titov, N., Schofield, C., Staples, L., Dear, B. F. & Nielssen, O. A comparison of Indigenous and non-Indigenous users of MindSpot: an Australian digital mental health service. *Australas. Psychiatry* **27,** 352–357 (2018).
77. Ünlü Ince, B. *et al.* Internet-based, culturally sensitive, problem-solving therapy for Turkish migrants with depression: randomized controlled trial. *J. Med. Internet Res.* **15,** e227 (2013).
78. Van Straten, A., Cuijpers, P. & Smits, N. Effectiveness of a web-based self-help intervention for symptoms of depression, anxiety, and stress: randomized controlled trial. *J. Med. Internet Res.* **10,** 1–11 (2008).
79. Wang, Z., Wang, J. & Maercker, A. Chinese My Trauma Recovery, a web-based intervention for traumatized persons in two parallel samples: randomized controlled trial. *J. Med. Internet Res.* **15,** 112–125 (2013).
80. Steinmetz, S. E., Benight, C. C., Bishop, S. L. & James, L. E. My Disaster Recovery: a pilot randomized controlled trial of an Internet intervention. *Anxiety, Stress Coping* **25,** 593–600 (2012).
81. Wasil, A. R. *et al.* Harnessing single-session interventions to improve adolescent mental health and well-being in India: development, adaptation, and pilot testing of online single-session interventions in Indian secondary schools. *Asian J. Psychiatr.* **50,** 101980 (2020).
82. Schleider, J. L. & Weisz, J. R. Reducing risk for anxiety and depression in adolescents: effects of a single-session intervention teaching that personality can change. *Behav. Res. Ther.* **87,** 170–181 (2016).
83. Yokomitsu, K. *et al.* Gamified mobile computerized cognitive behavioral therapy for Japanese university students with depressive symptoms: protocol for a randomized controlled trial. *JMIR Res. Protoc.* **9,** 1–10 (2020).

84. Merry, S. N. *et al.* The effectiveness of SPARX, a computerised self help intervention for adolescents seeking help for depression: randomised controlled non-inferiority trial. *BMJ* **344,** 1–16 (2012).
85. Sirriyeh, R., Lawton, R., Gardner, P. & Armitage, G. Reviewing studies with diverse designs: the development and evaluation of a new tool. *J. Eval. Clin. Pract.* **18,** 746–752 (2012).
86. Arjadi, R., Nauta, M. H. & Bockting, C. L. H. Acceptability of internet-based interventions for depression in Indonesia. *Internet Interv.* **13,** 8–15 (2018).
87. Airhihenbuwa, C. O. A conceptual model for culturally appropriate health education programs in developing countries. *Int. Q. Community Health Educ.* **11,** 53–62 (1990).
88. Taloyan, M., Johansson, S. E., Sundquist, J., Koctürk, T. O. & Johansson, L. M. Psychological distress among Kurdish immigrants in Sweden. *Scand. J. Public Health* **36,** 190–196 (2008).
